# The Microbial and Metabolic Signatures of Patients with Stable Coronary Artery Disease

**DOI:** 10.1128/spectrum.02467-22

**Published:** 2022-11-10

**Authors:** Jing Zhong, Dingfeng Wu, Yuanyuan Zeng, Gaosong Wu, Ningning Zheng, Wenjin Huang, Yan Li, Xin Tao, Weize Zhu, Lili Sheng, Xiaoxu Shen, Weidong Zhang, Ruixin Zhu, Houkai Li

**Affiliations:** a School of Pharmacy, Shanghai University of Traditional Chinese Medicine, Shanghai, China; b Huzhou Key Laboratory of Molecular Medicine, Huzhou Central Hospital, Affiliated Central Hospital Huzhou University, Huzhou, China; c National Clinical Research Center for Child Health, the Children's Hospital, Zhejiang University School of Medicine, Hangzhou, Zhejiang, People’s Republic of China; d Cardiology Department of Dongzhimen Hospital, Beijing University of Chinese Medicine, Beijing, China; e The First Clinical Medical College, Beijing University of Chinese Medicine, Beijing, China; f Institute of Interdisciplinary Integrative Medicine Research, Shanghai University of Traditional Chinese Medicine, Shanghai, China; g Department of Phytochemistry, School of Pharmacy, Second Military Medical University, Shanghai, China; h The Shanghai Tenth People's Hospital, School of Life Sciences and Technology, Tongji University, Shanghai, People’s Republic of China; Lerner Research Institute

**Keywords:** gut microbiome, *Ralstonia pickettii*, serum metabolome, stable coronary artery disease, unsaturated fatty acid

## Abstract

Growing evidence indicates an association between gut dysbiosis and coronary artery disease (CAD). However, the underlying mechanisms relevant to stable CAD (SCAD) pathogenesis, based on microbe-host metabolism interactions, are poorly explored. Here, we constructed a quasi-paired cohort based on the metabolic background of metagenomic samples by the propensity score matching (PSM) principle. Compared to healthy controls (HCs), gut microbiome disturbances were observed in SCAD patients, accompanied by differences in serum metabolome, mainly including elevated acylcarnitine and decreased unsaturated fatty acids in SCAD patients, which implicated the reduced cardiac fatty acid oxidation. Moreover, we identified Ralstonia pickettii as the core strain responsible for impaired microbial homeostasis in SCAD patientsm and may be partly responsible for the decrease of host unsaturated fatty acid levels. These findings highlight the importance of unsaturated fatty acids, R. pickettii, and their interaction in the pathogenesis of SCAD.

**IMPORTANCE** Stable coronary artery disease (SCAD) is an early stage of CAD development. It is important to understand the pathogenesis of SCAD and find out the possible prevention and control targets for delaying the progression of CAD. We observed reduced levels of unsaturated fatty acids (USFAs) in SCAD patients. However, the reduced USFAs may be related to Ralstonia Pickettii, which was the core strain responsible for the impaired gut microbial function in SCAD patients, and further affected the host's cardiovascular health by altering amino acids, vitamin B metabolism, and LPS biosynthesis. These findings not only emphasized the importance of USFAs for cardiovascular health, but also R. Pickettii for maintaining microbial function homeostasis. More importantly, our study revealed, for the first time, that enriched R. Pickettii might be responsible for the reduced USFAs in SCAD patients, which adds new evidence on the role of altered gut microbiota for SCAD formation.

## INTRODUCTION

Cardiovascular diseases are the leading cause of death worldwide (1). Coronary artery disease (CAD) is the most common cardiovascular disease and is divided into 3 categories, according to its clinical symptoms and the degrees of arterial obstruction and myocardial damage: stable CAD (SCAD), unstable angina (UA), and myocardial infarction (MI), which also represent the different stages of CAD progression (2). CAD is mainly driven by atherosclerosis, involving complex and diverse causes in formation and progression. As an early stage of the disease, SCAD only occurs with obvious chest pain symptoms when the cardiac load suddenly increases, but it is a key node in the progression of the disease. Hence, understanding the pathogenesis of SCAD is particularly critical to improve clinical outcomes.

Environmental factors are more active than genetic factors in the pathogenesis of CAD. As a main environmental factor, diet exerts a profound influence on the susceptibility to CAD. Gut microbiota, the “metabolic organ” of diet, convert nutrients in foods into metabolites, subsequently interacting with the host to affect host metabolism. With the major advances in microbiome metagenomics and targeted metabolomics technologies, there is a need to discover microbes and their derived metabolites related to cardiovascular disease phenotypes, and to conduct corresponding research on the mechanism. The diet-derived microbial metabolites, such as trimethylamine-N-oxide (TMAO) (3), sphenylacetylglutamine (PAGln) (4), short-chain fatty acids (SCFA) (5), indole derivatives (6), and 5-hydroxytryptophane (7), were recently discovered to be closely related to CAD progression. However, the underling mechanisms of SCAD, based on microbe-host interactions, are largely unknown.

Microbial composition is affected by many factors, with large variation among individuals that is sometimes even greater than disease-related changes, and that profoundly affects the identification of disease-related microbial characteristics (8–11). Matching the host variables of comparison groups is the common method for human microbiota studies to increase robustness and reproducibility. Previous studies have shown that microbial components and abundance are strictly constrained by the entire metabolic network in the microbiome (12, 13), and the core metabolic functions of microbiome are stable among different individuals (functional redundancy) (14, 15). Thus, based on the microbial metabolic background, propensity score matching (PSM) is considered a good technique for adjusting inherent known confounder differences, and to help achieve a better balance between the disease groups and control groups (16–18).

In this study, stool and serum samples were collected from 42 patients with SCAD and 46 healthy individuals for whole-genome shotgun metagenomic analyses and targeted metabolomics. PSM was first used to pair the SCAD samples with control samples with a similar metabolic background in order to minimize the bias of individual diversity on the results of metagenomics. Based on this strategy, we were able to re-delineate changes in the composition and function of gut microbes and the profile of serum metabolites in SCAD patients to further reveal the interaction between gut microbes and host metabolism, and to discover more about the involvement of gut microbes in the pathogenesis of SCAD.

## RESULTS

### The characteristics of the study population based on the PSM strategy.

We performed shotgun metagenomic sequencing of fecal samples from 42 SCAD patients and 46 healthy controls (HCs). The demographics and clinical characteristics of the SCAD patients and HCs are all presented in Table S1. A total of 81% of the SCAD patients were concomitant with one or more of the conditions of hypertension, diabetes, and hyperlipemia, whereas only 57% of the HCs were concomitant with these diseases. Indeed, all these diseases are risk factors for coronary heart disease. Meanwhile, age, high-density lipoprotein (HDL), Triglyceride (TG), and fasting blood glucose (FBG) were the main differences between the 2 groups.

**FIG 1:**
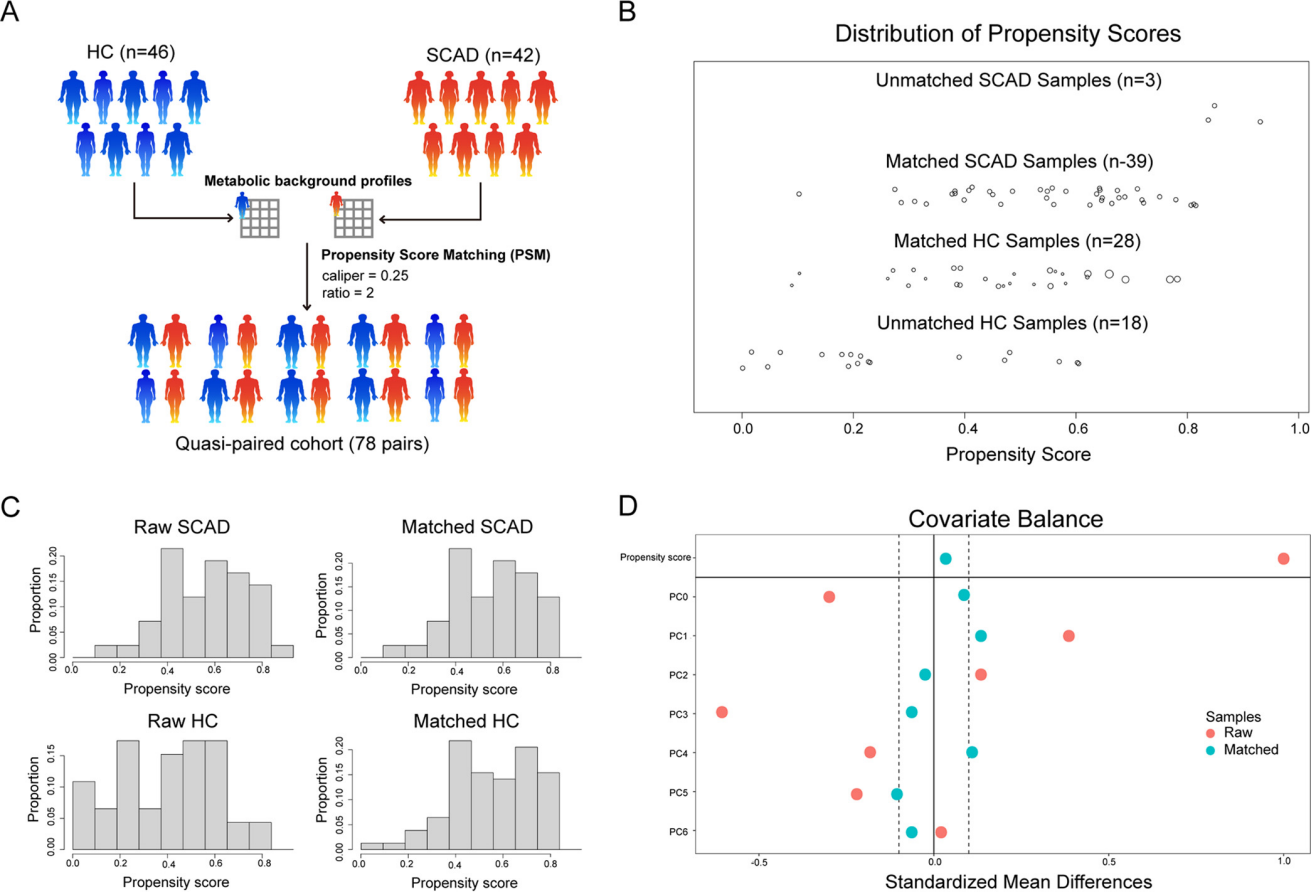
Construction of quasi-paired cohort by propensity score matching. (A) Samples were matched by propensity score matching with caliper = 0.25 and ratio = 2 based on their metabolic background profiles. (B) The distribution of propensity score before/after matching. (C) Sample proportion based on propensity score before/after matching. (D) Covariate balance of the metabolic background principal components before/after matching.

Due to the limited sample size of the cohort and large individual differences in gut microbes, gut microbiomes showed no significant differences between SCAD patients and HCs by conventional analysis, including α-diversity, β-diversity, and metabolic pathways(*P*_FDR_ > 0.05) (Fig. S1A and B, Table S2). We next conducted re-analysis based on the PSM strategy on metabolic profiles of gut microbes. In doing so, we constructed a cohort of 78 SCAD-control pairs (for details, please see the materials and methods), which ultimately included 39 SCAD patients and 28 HCs from the original groups based on the metabolic background of the samples (Fig. 1). All subsequent analyses were based on these matching samples, and the new demographics and clinical characteristics of 78 SCAD-control pairs are presented in Table S3. Meanwhile, the influence of age and FBG difference on the microbial metabolic background were weakened after matching, fully demonstrating the power of the PSM strategy (Fig. S1C).

### Reduced alpha diversity and altered microbial composition in SCAD.

The sequences were analyzed using MetaPhlAn3 to profile the composition of microbial communities. In this cohort, most bacterial read counts were dominated by *Bacteroidetes* (66.15%), *Firmicutes* (16.70%) and *Proteobacteria* (11.70%), followed by *Actinobacteria* (4.11%) and *Verrucomicrobia* (1.18%), which covered 99% of gut microbes in SCAD and HCs. The phyla did not differ significantly in abundance between the 2 groups (Fig. S2A). The ratio of Bacteroidetes to *Firmicutes* had no obvious difference between the 2 groups (Fig. S2B). Furthermore, about 96% of the microbes were accounted for the 30 most abundant genera. Statistically, all the differential genera between the 2 groups were decreased in SCAD compared with HCs, including Paraprevotella, Barnesiella, Phascolarctobacterium, Faecalibacterium, Lachnospira, and Clostridium (Fig. S2C). Besides, there were 21 differential genera between the 2 groups, of which only 3 were more abundant in the SCAD patients (Table S4), including Ralstonia, Enterococcus, and Megasphaera. Collectively, most of the differential genera exhibited a declining variation in the SCAD group.

**FIG 2:**
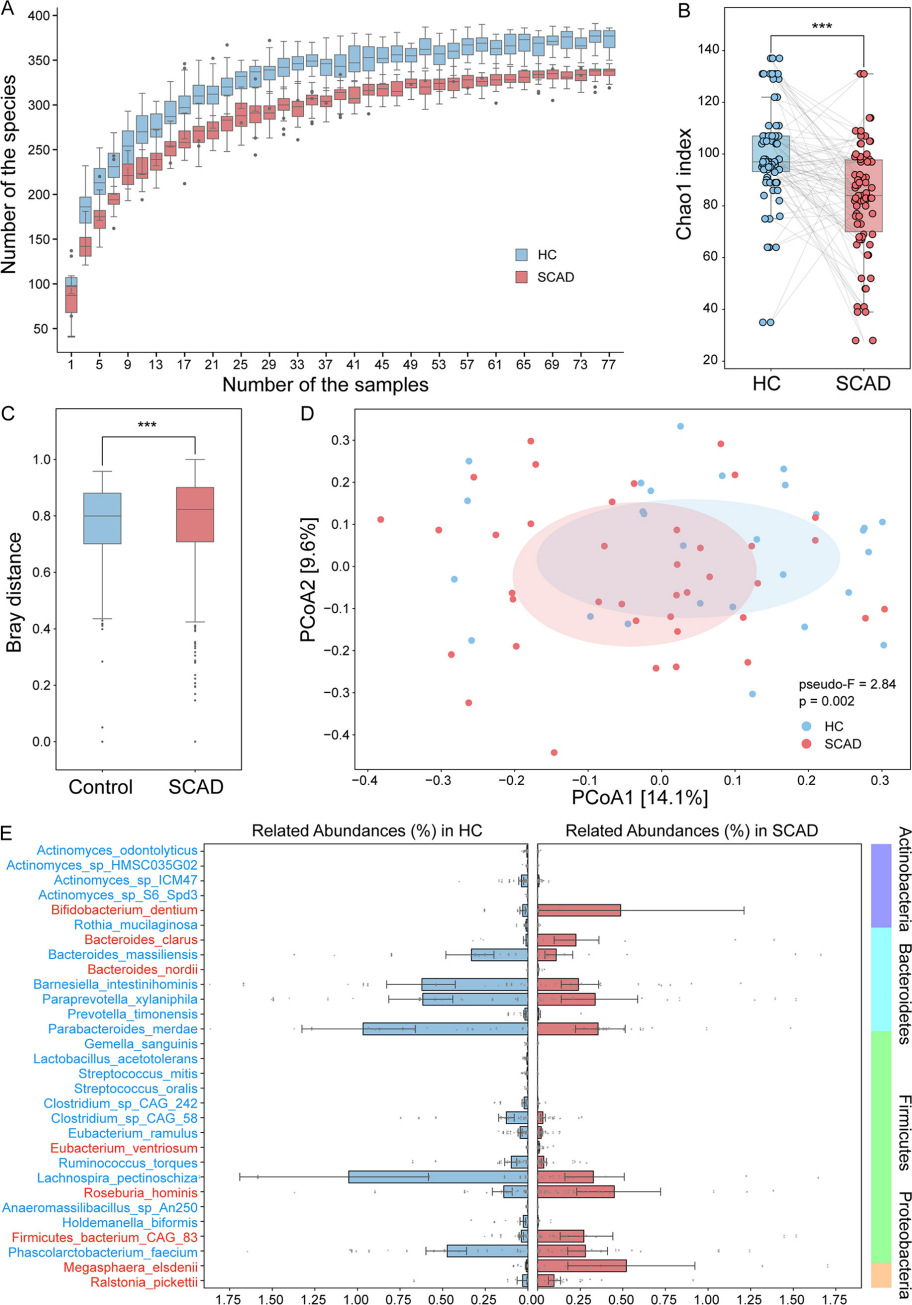
Gut microbial alterations in SCAD patients. (A) The species number rarefaction curves. (B) Alpha diversity measured by Chao1 index at species level. Wilcoxon signed-rank test was used to determine the significance. ***, *P < *0.001. (C) and (D) Beta diversity based on Bray-Curtis distance and unweighted unifrac distance at species level. Wilcoxon rank-sum test and PERMANOVA test were used to determine the significance. ***, *P < *0.001. (E) Relative abundances of 29 differential bacterial species between SCAD patients and HCs (Wilcoxon signed-rank test, *P*_FDR_ < 0.05).

As shotgun metagenomic sequence can pinpoint the species level of gut microbiota. Thus, we performed the species rarefaction curve for each sample and found that the sequencing depth was adequate. Regardless of the number of samples, SCAD patients always exhibited fewer species richness than HCs (Fig. 2A). Further, a reduced α-diversity in SCAD in comparison with HCs was confirmed by the less Chao1 Index at the species level (*P = *1.005e-06, Wilcoxon signed-rank test) (Fig. 2B). Then, the principal coordinated analysis (PCoA), based on Bray-Curtis distance and unweighted_unifrac distance, was performed to evaluate the variation in community composition. The SCAD groups significantly deviated in overall composition structure from HCs (Wilcoxon rank-sum test, *P = *3.091e-05; PERMANOVA test, pseudo-F:2.84, and *P = *0.001) (Fig. 2C and D).

Moreover, we identified the taxonomic abundances at the species level. A total of 29 species differed significantly between the 2 groups, mainly belonging to *Actinobacteria* (5 species), *Bacteroidetes* (7 species), *Firmicutes* (16 species), and *Proteobacteria* (1 species) (Wilcoxon signed-rank test, *P*_FDR_ < 0.05), including 8 SCAD-enriched species and 21 HC-enriched species (Fig. 2E and Table S5). Of note, among these significantly altered species, Megasphaera elsdenii was 55 times more abundant in SCAD patients than in HCs. Moreover, another interesting thing was that a significant proportion of these differential bacteria were odontogenic bacteria, such as Bifidobacterium dentium, Streptococcus mitis, Streptococcus oralis, Rothia mucilaginosa, and *Actinomyces* spp. (4 species).

### Functional alterations of gut microbiome in SCAD.

**FIG 3:**
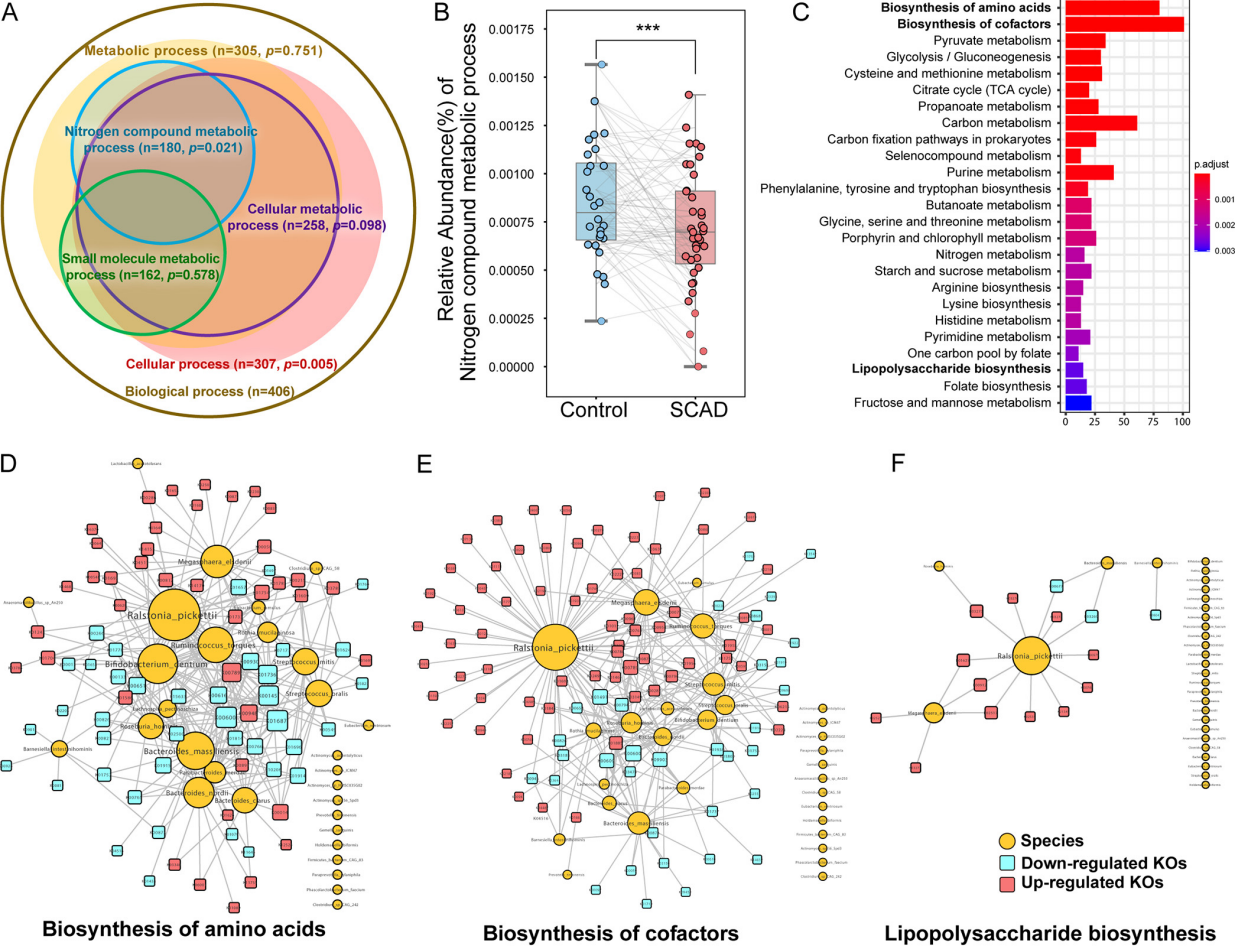
Functional alteration of gut microbes in SCAD patients. (A) Key GO groups enriched by differential GO terms between the 2 groups by Fisher’s exact test. (B) Relative abundance of nitrogen compound metabolic process by GO enrichment analysis. Wilcoxon signed-rank test was used to determine the significance. ***, *P < *0.001. (C) Metabolic pathways enrichment of 1371 differential KO genes (Wilcoxon signed-rank test, *P*_FDR_ < 0.05) between the 2 groups based on KEGG database by clusterProfiler. (D) to (F) Associated networks constructed from differential gut microbes and their KO genes in amino acid biosynthesis, cofactors biosynthesis, and LPS biosynthesis. Yellow circulars represent differential gut microbes, and the size denotes the connected numbers of KO genes. Red squares represent upregulated differential KO genes, and blue squares represent downregulated differential KO genes.

The function of gut microbes is a key point to elucidate the relationship between intestinal flora and disease, nor is the function of many specific species not well understood. However, metagenomic analysis is useful for revealing the microbial functions. After enrichment of differential gene oncology (GO) terms between the 2 groups, we found that most of the nitrogen compound metabolic process-related GO terms were differentially enriched in the SCAD group (*n* = 180, *P = *0.021) (Fig. 3A). We further calculated the relative abundance of nitrogen metabolic process between the 2 groups, which showed that the SCAD group was significantly reduced compared to the HCs (*P < *0.001) (Fig. 3B). These results suggest that the intestinal flora involved in nitrogen compound metabolism were disturbed in SCAD patients.

Meanwhile, a total of 1371 differential KEGG Ontology genes (KOs) of 1990, were identified between the 2 groups when we aligned the clean sequences to KEGG (431 and 940 KOs enriched in HCs and SCAD patients; *P*_FDR_ < 0.05) (Table S6). These differential KOs were involved in 37 metabolic pathways (*P*_FDR_ < 0.05) (Fig. 3C and Table S7). Mainly SCAD patients had disturbed amino acid biosynthesis (10 pathways), cofactor biosynthesis (8 pathways), and carbohydrate metabolism (10 pathways).

Amino acid is a class of important substrates for bacterial energy metabolism. In this study, we observed that the disturbance of amino acid metabolism in SCAD patients were the most apparent (Fig. 3C) (Qvalue = 3.79e-24). The metagenomic data of SCAD patients showed higher abundance for genes involved in phenylalanine, tyrosine, and tryptophan biosynthesis (*P*_FDR_ = 3.01e-06), while lower abundance for genes in glycine, serine and threonine metabolism, lysine biosynthesis, cysteine and methionine metabolism, arginine biosynthesis, valine, leucine, and isoleucine biosynthesis (*P*_FDR_ < 0.01) (Fig. S3 and 4). The major microbial contributors were *Betaproteobacteria* spp. (Ralstonia pickettii), *Actinobacteria* spp. (R. mucilaginosa, B. dentium), *Bacteroidia* spp. (Bacteroides clarus, Bacteroides massiliensis, Bacteroides nordii, Parabacteroides merdae), *Bacilli* spp. (S. mitis, S. oralis), *Clostridia* spp. (Ruminococcus torques), and *Negativicutes* spp. (M. elsdenii), which contained a relatively large number of amino acids related to KO genes (Fig. 3D). These results showed the altered intestinal bacteria functions involved in amino acid metabolism in SCAD patients compared to HCs.

Coenzyme is a general term for a large group of organic cofactors, which are essential factors for enzyme-catalyzed REDOX reactions, group transfer, and isomerization reactions (19). In addition to dietary supplementation, intestinal bacteria can also participate in the metabolism of these compounds (20). Similar to amino acid metabolism, coenzyme metabolism disorder was another significantly changed microbial function in SCAD patients, with the most altered KO genes, and mainly referring to B vitamins metabolism (Fig. 3C) (Qvalue = 1.96e-22), although the specific pathways were not statistically significant between SCAD patients and HCs (Fig. S5). The major microbial contributors were *Betaproteobacteria* spp. (R. pickettii), *Actinobacteria* spp. (R. mucilaginosa, B. dentium), *Bacteroidia* spp. (B. clarus, B. massiliensis, B. nordii, P. merdae), *Bacilli* spp. (S. mitis, S. oralis, Lactobacillus acetotolerans), *Clostridia* spp. (R. torques), and *Negativicutes* spp. (M. elsdenii), which were nearly the same as the amino acid metabolism related microbes (Fig. 3E). This may be due to the close relationship between amino acids and coenzymes, since amino acid derivatives are always the precursors of coenzymes, which are also indispensable molecules in amino acid metabolism.

CAD patients have been reported to have higher fecal LPS levels than controls (21), and the bowel wall edema and impaired barrier function was often found in heart failure patients, which leads to translocation of LPS into circulation (22). LPS can promote the development of atherosclerosis by inducing endothelial cell injury, stimulating monocyte oxidative metabolism and LDL oxidation (23). We observed enrichment of genes of LPS synthesis in SCAD patients (Fig. 3C) (Qvalue = 1.78e-03). The major contributors were *Betaproteobacteria* spp. (R. pickettii) and *Negativicutes* spp. (M. elsdenii) (Fig. 3F).

Overall, the biosynthesis of amino acid and cofactors was the main functional change of differential flora in SCAD patients. The R. pickettii and M. elsdenii appeared to be the most important contributors. Further, we also observed abnormal glycometabolism, such as pyruvate metabolism, glycolysis/gluconeogenesis, and the TCA cycle, which may cross talk with amino acid metabolism. The changes of these bacterial derivatives may communicate with the host through different mechanisms, and ultimately have physiological or pathological impacts on the host.

### Serum metabolome alterations in SCAD.

**FIG 4:**
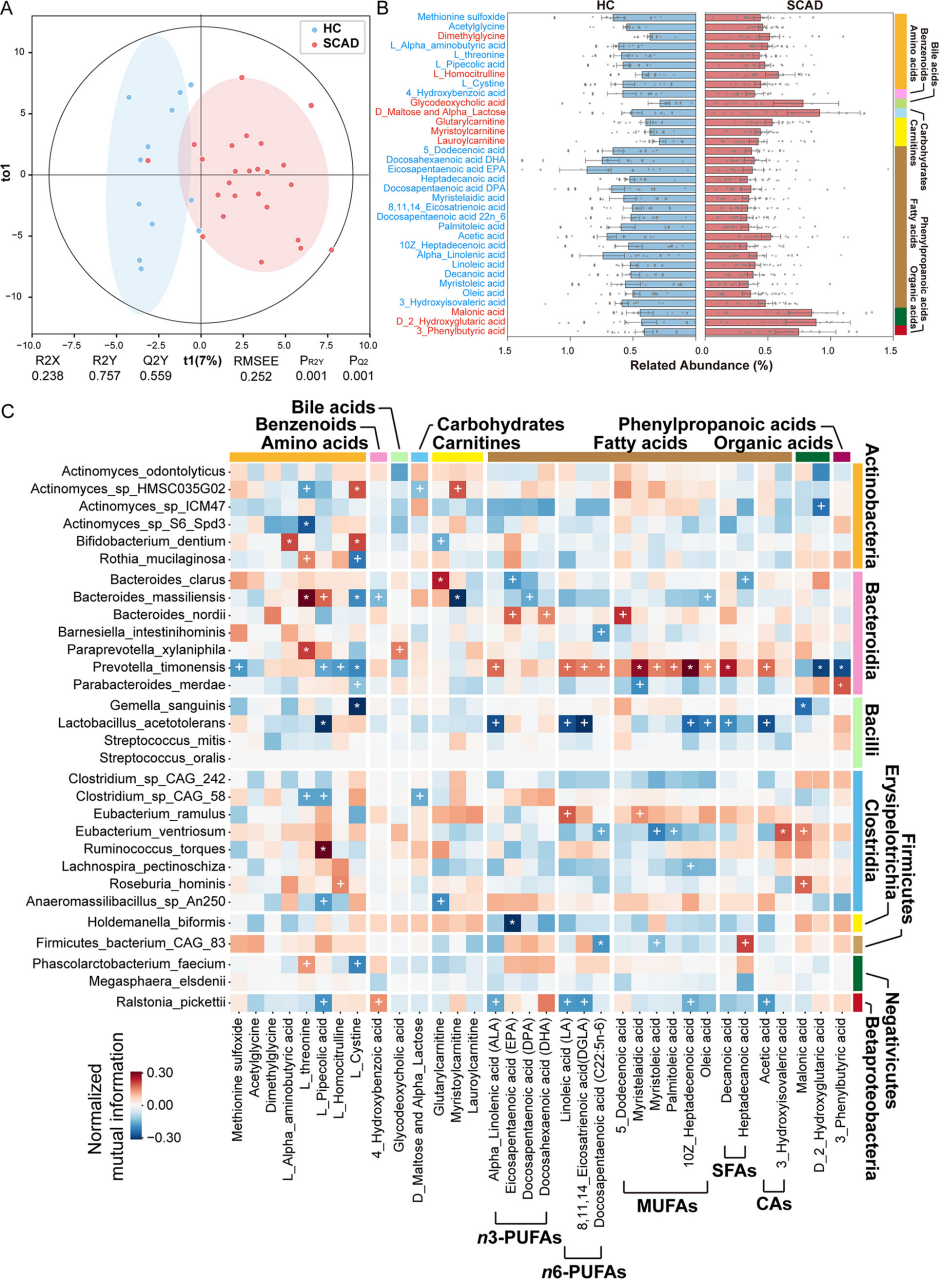
Serum metabolome alterations in SCAD patients. (A) The OrthoPLSDA model of serum metabolome analysis was performed on SCAD patients and HCs (Empirical *P*_Q2_ < 0.001 and *P*_R2Y_ < 0.001). (B) Relative abundances of 34 differential serum metabolites between SCAD patients and HCs (Wilcoxon signed-rank test, *P*_FDR_ < 0.05). (C) The heatmap depicts relationships between the SCAD-related gut microbes and serum metabolites. The signs of change trend for each paired samples in cohort were calculated, and then normalized mutual information score was used to evaluate the association of change trends. Normalized mutual information score ranges from 0 to 1, indicating that the association between the 2 trends ranges from weak to strong. The sign of score was evaluated by the direction consistency of 2 trends. Permutation test (1000 permutations) was performed to determine *P*-value of correlation. * and **^+^** suggest the significance, **^+^**, *P < *0.05; *, *P < *0.01.

Given the interplay between the gut microbiome and host metabolism, we performed targeted metabolomics on serum samples from parts of SCAD patients and HCs (SCAD *n* = 25 and HC *n* = 12), followed by between-group difference analysis with the same matching method as gut metagenome. In this study, we detected a total of 306 metabolites by UPLS-MS/MS, including 60 amino acids, 40 bile acids, 26 carbohydrates, 34 organic acids, 55 fatty acids, 29 benzenoids, 21 carnitines, 9 indoles, and 32 other metabolites. The OrthoPLSDA model revealed that the serum metabolic signatures of SCAD patients significantly deviated from HCs (Empirical *P*-values *P*_Q2_ < 0.001 and *P*_R2Y_ < 0.001) (Fig. 4A).

Compared with HCs, the SCAD patients displayed enrichment in 10 metabolites and depletion in 24 metabolites, which mainly comprised of 8 amino acids, 17 fatty acids, and 3 carnitines (Fig. 4B and Table S8). Notably, almost all altered fatty acids were unsaturated fatty acids, including polyunsaturated fatty acid, such as n-3 PUFAs (alpha_linolenic acid, docosahexaenoic acid DHA, eicosapentaenoic acid EPA, docosapentaenoic acid DPA), n-6 PUFAs (linoleic acid, docosapentaenoic acid 22n_6, 8,11,14_eicosatrienoic acid), and monounsaturated fatty acids (5_dodecenoic acid, myristelaidic acid, myristoleic acid, palmitoleic acid, 10Z_heptadecenoic acid, oleic acid). In addition, among the 8 amino acids altered in SCAD, the majority (*n* = 6) were decreased in SCAD patients, such as acetylglycine, l-threonine, methionine sulfoxide, L-cystine, L-pipecolic acid, and L-alpha-aminobutyric acid (Fig. 4B and Table S8). This feature was consistent with the disorder of amino acid metabolism in intestinal flora. On the contrary, all the acylcarnitines (myristoylcarnitine, lauroylcarnitine, glutarylcarnitine) were elevated in SCAD patients (Fig. 4B and Table S8).

Meanwhile, the KEGG metabolic pathway enrichment showed that fatty acid biosynthesis, especially unsaturated fatty acid synthesis, was the most significant change in the SCAD patients (Fig. S6A). Also, the human disease enrichment analysis showed that these altered metabolites were significantly correlated with hypertension, myocardial injury, and heart failure, suggesting that these altered metabolites may play a role in causing cardiovascular diseases (Fig. S6B).

**FIG 5:**
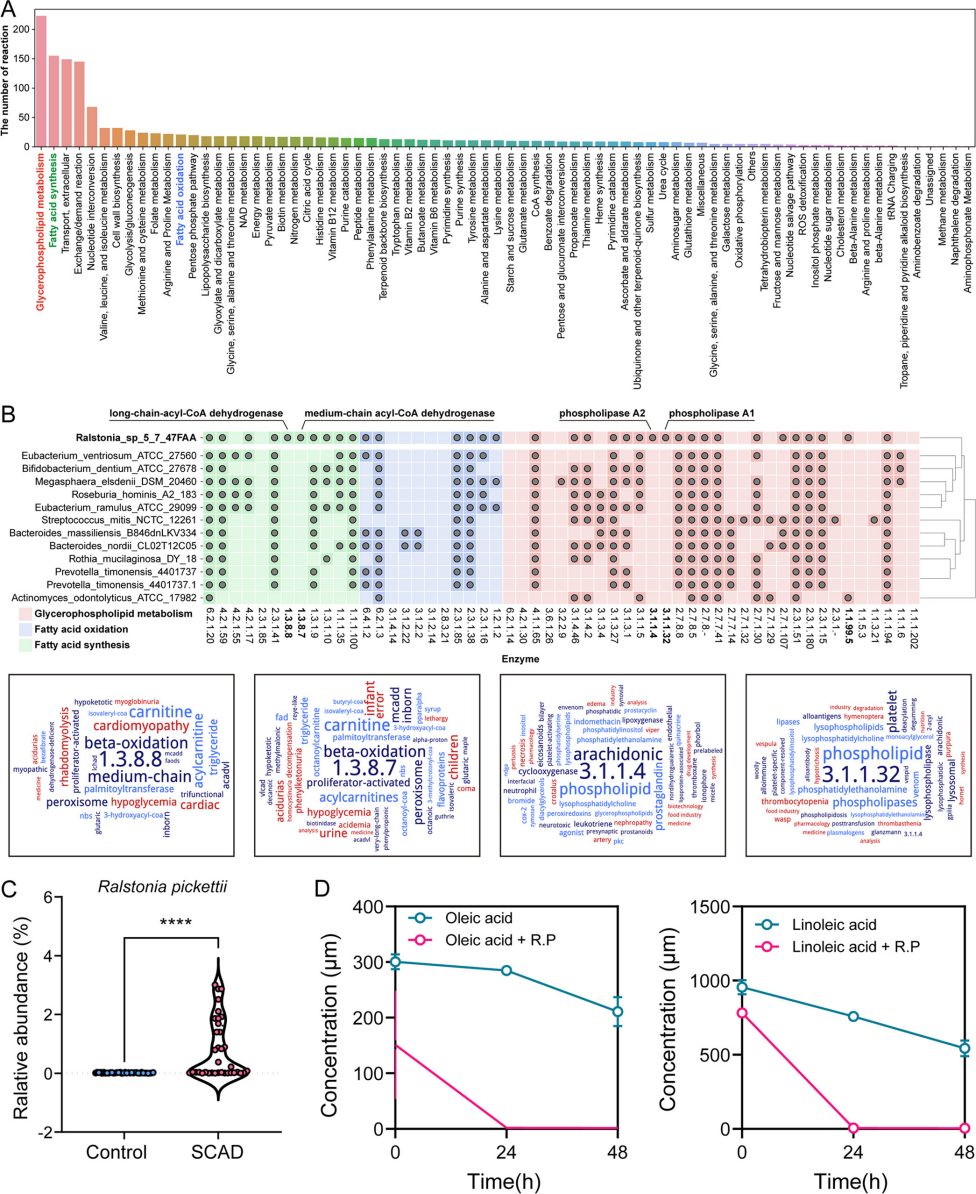
The relationship of Ralstonia Pickettii and unsaturated fatty acids. (A) The biological processes that *R. Pickettii* participated in. (B) Comparative genomic analysis of the fatty acid metabolizing enzymes between *R. Pickettii* and other species. Gray dot indicates that the taxon has the coding gene of the corresponding enzyme. Ward map of fatty acid metabolizing enzymes *R. pickettii* specifically encoded, phospholipase A1 (EC 3.1.1.32), phospholipase A2 (EC 3.1.1.4), medium-chain acyl-CoA dehydrogenase (EC 1.3.8.7), and long-chain acyl-CoA dehydrogenase (EC 1.3.8.8). (C) The relative abundance of *R. pickettii* based on qRT-PCR in the matched cohort. (D) The concentration of sodium oleate and sodium linoleate after cocultured *R. pickettii* (ATCC27511) with sodium oleate (300 μM) and sodium linoleate (800 μM) for 48 h (*n* = 3). Wilcoxon rank-sum test was used to determine the significance. ****, *P < *0.0001.

Next, we performed correlation analysis of each differential metabolite with SCAD-linked microbiota (Fig. 4C). Notably, we observed strong positive associations between Prevotella timonensis and unsaturated fatty acids that were both decreased in SCAD, as well as negative associations between R. pickettii, Eubacterium ventriosum, and unsaturated fatty acids. We first validated that the level of unsaturated fatty acid was significantly decreased in SCAD patients in another independent cohort (Fig. S7), and the relative abundance of R. pickettii was significantly enriched in SCAD patients using qPCR in the matched cohort (Fig. 5C). Considering that R. pickettii was the most important contributor of impaired gut microbial function, we further performed a correlation analysis between the differential fatty acids and microbial functions (GO: biological process) (Fig. S8). We observed that most of the unsaturated fatty acids, such as ALA (alpha linolenic acid), LA (linoleic acid), DGLA (8,11,14_eicosatrienoic acid), 10Z_heptadecenoic acid, oleic acid and SFAs (decanoic acid), and SCFAs (acetic acid) had a robust association with microbial function in amino acid metabolism (i.e., selenocysteine biosynthesis process, proline transport, histidine metabolic process, tryptophan catabolic process, and l-lysine catabolic process) and cofactors metabolism (i.e., tetrahydrobiopterin biosynthetic process, regulation of ubiquinone biosynthetic process, and folic acid-containing compound metabolic process). Further, we analyzed the metabolic process and enzymes of R. pickettii by its genome-scale metabolic model (Fig. 5A, and B), showing that R. pickettii participated in many biological activities, including Fatty acid synthesis and Fatty acid oxidation (Fig. 5A). Comparing the genome with other strains, R. pickettii encoded a relatively complete set of fatty acid metabolizing enzymes (Fig. 5B), such as phospholipase A1 (EC 3.1.1.32), phospholipase A2 (EC 3.1.1.4), medium-chain acyl-CoA dehydrogenase (EC 1.3.8.7), and long-chain acyl-CoA dehydrogenase (EC 1.3.8.8). The ability of R. pickettii to metabolize USFAs was confirmed by our experiments, showing that sodium oleate (300 μM) and sodium linoleate (800 μM) were almost metabolized after co-culturation of R. pickettii (ATCC27511) with sodium oleate and sodium linoleate for 24 h (Fig. 5D).

Moreover, the decreased levels of L-pipecolic acid, one of lysine metabolites, observed in SCAD patients was positively correlated with HCs-enriched B. massiliensis, R. torques, while negatively associated with R. pickettii. In addition, B. massiliensis had strong positive association with l-threonine. The correlation between microbiota abundance and metabolites concentration may be related to the corresponding threonine and lysine metabolism functions of these microbiota (Fig. 3D). Glutarylcarnitine and myristoylcarnitine, a short- or long-chain acylcarnitine, elevated in the SCAD group, had strong correlation with *Bacteroides* spp (Fig. 4C).

### Integrated analysis between gut microbiome and serum metabolic signatures of SCAD.

**FIG 6:**
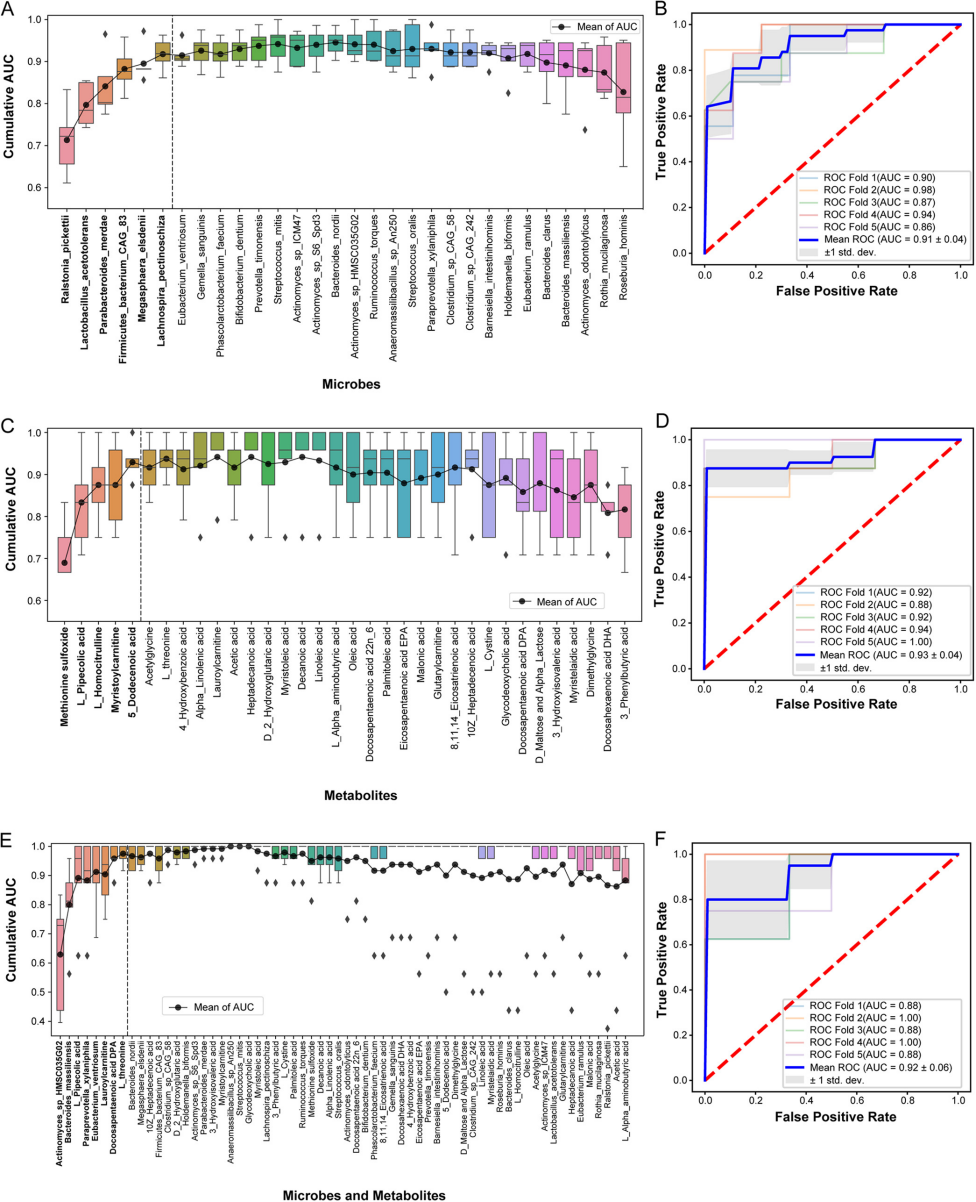
Disease status classification using SCAD-related gut microbes and serum metabolites. (A), (C), and (E) Cumulative AUC of model by recursive feature elimination based on SCAD-related microbial species, serum metabolites and their combination, respectively. Dash line indicated the best features selected for final model. (B), (D), and (F) ROC of the 5-fold cross-validated random forest classifiers composed of the selected microbial species, serum metabolites, and their combination, respectively.

To explore the potential of gut microbiota and metabolic profiles in SCAD prediction, we constructed a random forest classifier based on differential gut taxonomic or metabolic features from this cohort. A 5-fold cross-validated random forest model was used to screen the key discriminatory microbial species or metabolites. We were able to detect SCAD patients accurately based on the 6 differential gut microbial species composed of R. pickettii, M. elsdenii, *Firmicutes bacterium CAG83*, Lachnospira pectinoschiza, L. acetotolerans, and P. merdae, as indicated by an area under the receiver operating curve (AUC) of up to 0.91 (Fig. 6A and B). Besides, the model based on 5 metabolites composed of Methionine sulfoxide, L-pipecolic acid, L-homocitrulline, myristoylcarnitine, and 5-dodecenoic acid had similar performance to microbial features with an AUC of 0.93 (Fig. 6C and D), while the integrated metabolic and microbial features including 4 species and 4 metabolites had comparable diagnostic power with an AUC of 0.92 (Fig. 6E and F). Taken together, these data indicated that the predictive model based on intestinal flora, or the combination with differential metabolites, was sufficient to distinguish SCAD patients from HCs, highlighting the potential of intestinal flora for the noninvasive detection of populations with SCADs.

### Links between the differential gut microbes and serum metabolites with clinical features of SCAD.

**FIG 7:**
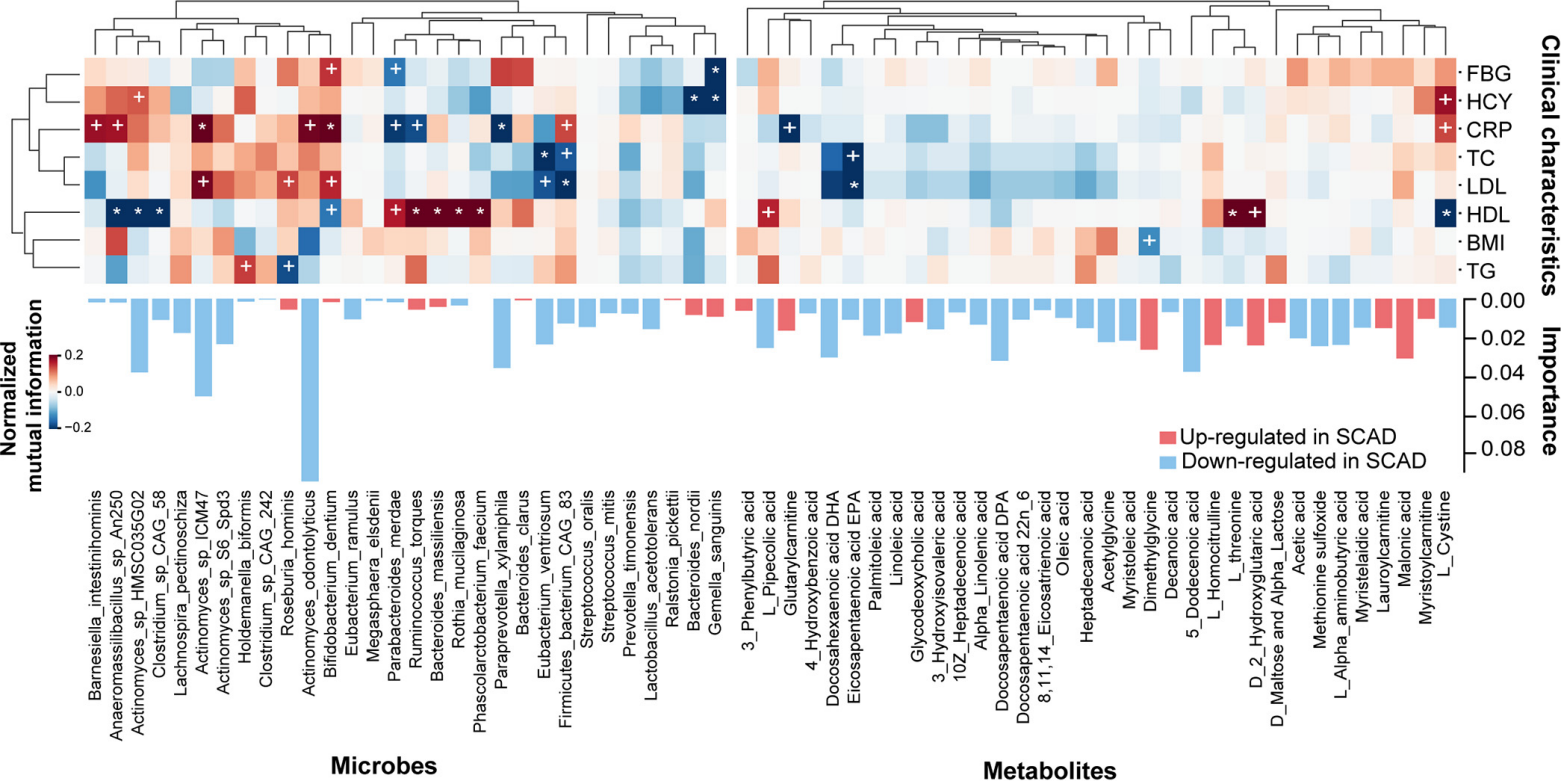
The association heatmap of microbial species, serum metabolites, and clinical parameters. The signs of change trend for each paired samples in cohort were calculated, and then normalized mutual information score was used to evaluate the association of change trends. Normalized mutual information score ranges from 0 to 1, indicating that the association between the 2 trends ranges from weak to strong. The sign of score was evaluated by the direction consistency of 2 trends. Permutation test (1000 permutations) was performed to determine *P*-value of correlation. * and **^+^** suggest the significance, **^+^**, *P < *0.05; *, *P < *0.01.

Besides being able to distinguish between individuals with SCAD or HCs, the differential gut microbes and serum metabolites showed associations with numbers of clinical indices. In general, the number of correlations between serum metabolites and clinical indices was significantly less than that of gut microbiota (Fig. 7).

As we know, HCY, TC, and LDL-C are independent risk factors for coronary heart disease, and CRP, a sensitive marker of chronic low-grade inflammation, is associated with an increased risk of incident coronary heart disease (24). In terms of metabolites, we observed that TC and LDL-C exhibited negative associations with eicosapentaenoic acid (EPA). In addition, we found that CRP was positively linked to B. dentium and *Firmicutes bacterium CAG83*, and inversely correlated with R. torques and P. merdae. Furthermore, HCY had a strong, negative correlation with Gemella sanguinis.

## DISCUSSION

In this study, we investigated the characters and interactions of gut microbes and host metabolism between SCAD patients and HCs, based on metagenomics and serum metabolomics approaches. Our results revealed that: (i) Cardiac fatty acid oxidation was significantly reduced in patients with SCAD, and low unsaturated fatty acid level in the serum further worsened cardiac energy metabolism; (ii) The serum level of unsaturated fatty acid was also negatively correlated with R. pickettii, which was the core species of gut microbe dysfunction in SCAD patients, as well as the most indispensable species for discriminating SCAD patients from HCs; (iii) R. pickettii may be partially responsible for the decrease of unsaturated fatty acid in the host.

Given the relatively small sample size, a PSM strategy was adopted to minimize the impacts of individual heterogeneity and cofounding factors among subjects based on their metabolic profile, as PSM is a well-established practical strategy for data alignment of a small size clinical study (18). Supported by the power of the PSM strategy, the matched cohort obtained more accurate and causal information compared with the cohort affected by confounders, while avoiding the blurring of true metabolic signals as much as possible. Our results showed that PSM strategy reduced the impacts of some cofounding factors of patients on data distribution, and generated 78 SCAD-HCs matching pairs for subsequent data analysis.

The heart is the most energy-demanding organ in the body and must constantly generate a large amount of ATP to maintain its contractile function. Thus, impairment of cardiac energy metabolism is the key cause of most heart diseases. The heart gets energy by metabolizing various fuels, such as fatty acids, glucose, lactate, ketone bodies, and amino acids, in which most of the ATP originate from the oxidation of fatty acids (25). Acylcarnitine plays an important role in fatty acid transportation through the mitochondrial membrane for oxidation. In this study, the serum levels of myristoylcarnitine and lauroylcarnitine of SCAD patients were significantly higher than those of the HCs, which suggests impairment of the mitochondrial fatty acid β-oxidation in the hearts of SCAD patients (26–28). Acylcarnitine has been reported to be a potential biomarker of cardiac metabolic disease in a growing number of studies (29), and are significantly associated with the risk of cardiovascular events, such as myocardial infarction in patients with stable angina (30, 31). Unlike medium- and long-chain acylcarnitines, abnormal levels of short-chain acylcarnitines are mainly attributable to disorders of branched-chain amino acid metabolism (32), while increased serum glutarylcarnitine levels in patients with SCAD suggests abnormal metabolism of branched-chain amino acids. Although branched-chain amino acids make up less than 2% of the total cardiac ATP production (33), recent studies have shown that branched-chain amino acids affect cardiac function by altering the cardiac insulin-signaling and mTOR-signaling pathways (34). All in all, our evidence at least indicates that cardiac energy metabolism of SCAD patients has undergone significant changes, mainly manifesting as a decrease in the utilization of fatty acids.

Fatty acid oxidation is critical for energy supply of cardiomyocytes, which is influenced by the availability of substrates of fatty acids and oxygen supply (35). The fluctuation of serum, endogenous metabolites are markers of metabolism status in peripheral organs like the liver, heart, and muscles (36). In this study, we observed that serum levels of 13 unsaturated fatty acids were significantly reduced in SCAD patients, including monounsaturated and polyunsaturated fatty acids. Unsaturated fatty acids, such as oleic acid, are the main fuels used for cardiomyocytes through oxidation due to its vast abundance and fast oxidation rate (35, 37). Reduced unsaturated fatty acid levels in SCAD patients also lead to a reduction in fatty acids supplied to the heart, which further reduces cardiac fatty acid oxidation, and greatly affects cardiac energy and function.

Polyunsaturated fatty acids are essential fatty acids for the human body. N-3 polyunsaturated fatty acids are negatively associated with occurrence of cardiovascular risk by reducing triglyceride levels, anti-inflammatory and anti-arrhythmic functions, lowering blood pressure, improving arterial and endothelial cell functions, and reducing platelet aggregation (38). Interestingly, our results also showed that the amount of unsaturated fatty acids, such as LA, ALA, DGLA, and 10Z_heptadecenoic acid, were significantly correlated with the abundance of P. timonensis and R. pickettii, which varied between SCAD and HCs groups. These data suggest that the decrease in serum fatty acid levels in SCAD patients may be related to gut microbes, in addition to diet.

L. acetotolerans, that has been published to have metabolic function for unsaturated fatty acids (39), was significantly reduced in SCAD patients (Fig. S9A), which may be related to the decreased levels of unsaturated fatty acids in the host. Meanwhile, the abundance of enterobacterial metabolic enzymes of polyunsaturated fatty acids was significantly increased in this study (Fig. S9B), suggesting that, in addition to *Lactobacillus*, other bacterial species may be involved in the metabolism of polyunsaturated fatty acids in a responsive mode. In particular, R. pickettii was found to be the most closely related with host levels of unsaturated fatty acids in our study, mainly due to its ability to metabolize unsaturated fatty acids, and may be partly responsible for the decrease in host levels of unsaturated fatty acids. In addition, both R. pickettii and L. acetotolerans were the main bacteria that distinguished SCAD patients from HCs, suggesting the importance of interaction between unsaturated fatty acids and gut microbes in SCAD formation.

As the core strain in the functional changes of intestinal microbiota in SCAD patients, R. pickettii is a non-fermenting Gram-negative bacillus present in the human gut (PRJNA375772, PRJNA382889, PRJNA434046, PRJEB11419, https://gmrepo.humangut.info), and is an opportunistic pathogen that often causes nosocomial infections (40). The LPS biosynthesis function of intestinal bacteria in SCAD patients was significantly enhanced, which was mainly related to R. pickettii encoding LPS biosynthesis genes. The previous study showed that R. pickettii contains the lipid A structure (41), which can increase the level of circulating LPS in mice (42), resulting in increased inflammatory reaction (41). Since chronic and systemic LPS-induced low-grade inflammation plays a key role in the onset and progression of cardiovascular diseases (43), the increased LPS biosynthesis induced by R. pickettii may be one of the causes of coronary heart disease. In addition, the gut microbes of SCAD patients showed disordered functions in amino acids and vitamin B biosynthesis, and many bacteria were involved in these functional changes (Fig. 3). However, R. pickettii was the bacterium with the most genes encoding these 2 metabolic pathways, with 42 and 59 KOs, respectively. Meanwhile, we also found a negative correlation between R. pickettii and serum acetate levels in SCAD patients. Acetic acid is the most abundant short-chain fatty acid (SCFA) present in serum, and the vast majority of circulating SCFAs are derived from gut microbial metabolism (44). Studies have shown that acetate induces endothelium-dependent vasodilation, reduces heart rate through the autonomic nervous system (45), and corrects cardiometabolic disturbances by inhibiting cardiac histone deacetylase (46), indicating that it may play a role in preventing hypertension and heart failure (47). R. pickettii may also affect the level of circulating acetic acid in the host by altering the abundance or function of acetogens, thereby affecting overall cardiac health.

**FIG 8:**
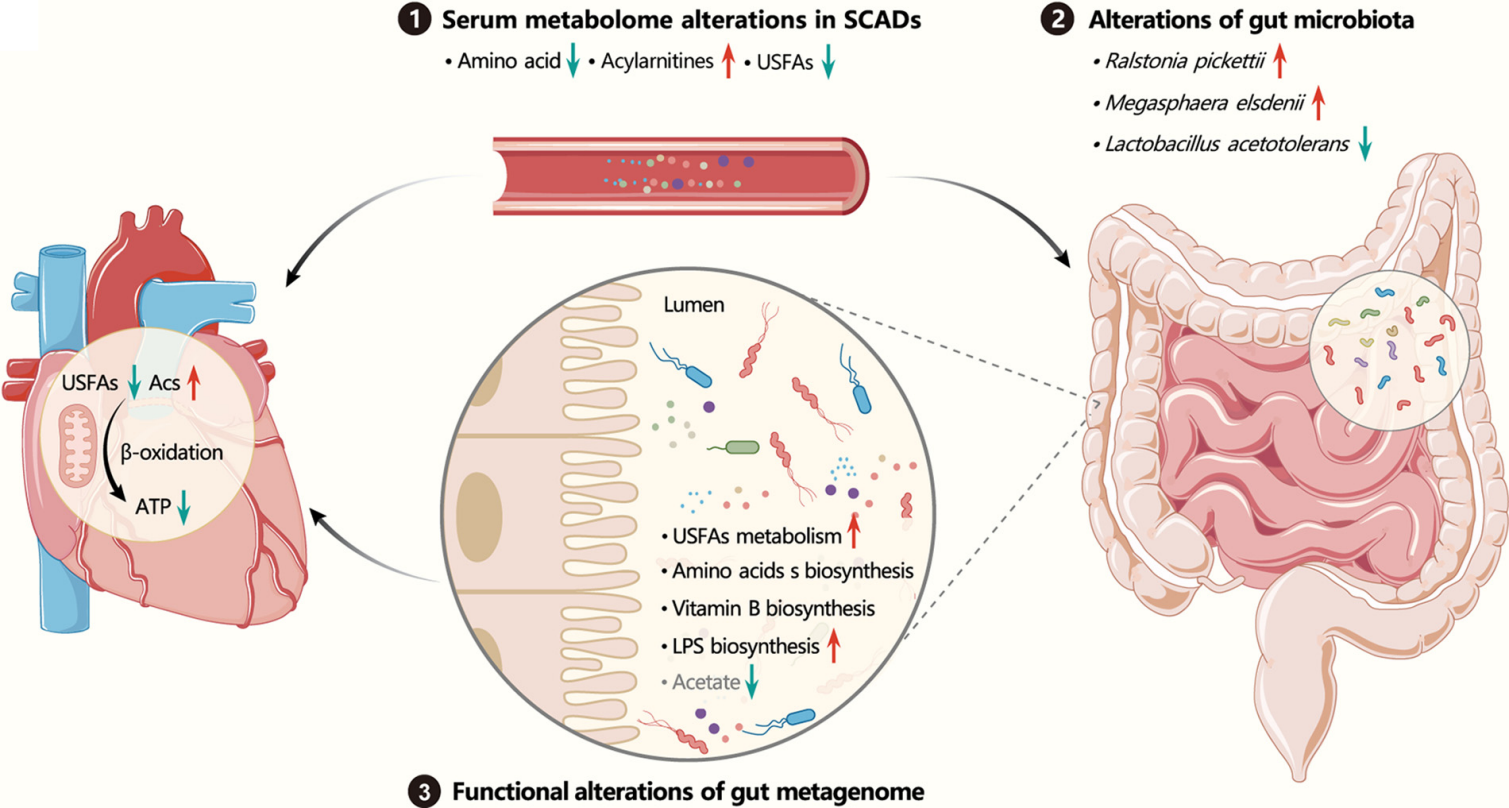
The cross talk between host and gut microbiota in SCAD patients. The increased acylcarnitine (Acs) levels suggests impairment of the mitochondrial fatty acid β-oxidation in SCAD patients, and the reduced unsaturated fatty acid levels would further reduce cardiac fatty acid oxidation and greatly affects cardiac energy and function. More importantly, the reduced unsaturated fatty acid level might be caused by Ralstonia pickettii, which holds a core position in the functional changes of intestinal microbiota. Further, R. pickettii may affect the heart health of host by changing the amino acid biosynthesis, B vitamin biosynthesis, LPS biosynthesis, and acetic acid production of intestinal flora.

In conclusion, based on constructing a quasi-matching cohort with PSM strategy, our study first identified the main changes in cardiac energy metabolism of SCAD patients, namely, the decrease in the utilization of fatty acids, by analyzing the characteristics of changes in serum metabolomics. The reduction in host unsaturated fatty acid levels might cause further deterioration of cardiac function in SCAD patients, not only because it is the main fuel for cardiac energy metabolism, but because it is closely related to R. pickettii, which holds a core position in the functional changes of intestinal microbiota and may affect the heart health of the host by changing the amino acid, vitamin B, LPS biosynthesis, and acetic acid production of intestinal flora (Fig. 8). Meanwhile, in addition to the diet, R. pickettii may be partially responsible for the decrease of unsaturated fatty acid. Taken together, we identified the main changes of microbial and metabolic signatures in SCAD patients and the link between them, highlighting the importance of USFAs-microbes interaction in the development of cardiovascular disease.

Limitations of the study include the sample size of the cohort, as it was relatively small, especially the serum samples for metabolomics that could match the intestinal flora samples. Though PSM based on microbial metabolic background was adopted to minimize the heterogeneity among individuals for clinical samples, further studies with larger sample sizes are still warranted to verify the main findings of this study, especially the diagnostic power of gut microbes or serum metabolites. Further, in line with the previous reports (20, 48–54), the link of identified differential serum metabolites, including amino acids, unsaturated fatty acids, vitamin B, or intestinal bacteria species with cardiovascular disease was reasonable. However, since these metabolites are co-metabolized by both host and gut microbes, the causative roles or the extent of their contributions of differential metabolites for SCAD development need further investigation.

## MATERIALS AND METHODS

### Study design and participants.

The patients with SCAD were from Dongzhimen Hospital affiliated with Beijing University of Chinese Medicine, between August 2018 to December 2019. The study was approved by the Ethics Committee of Dongzhimen Hospital affiliated with Beijing University of Chinese Medicine. All subjects gave written, informed consent.

The inclusion criteria for SCAD patients were: (i) age between 40 and 85 years; (ii) history of angina pectoris; (iii) myocardial ischemia, at rest, detected by electrocardiogram (ECG), or positive exercise stress test, or greater than 50% stenosis in at least 1 main branch detected by coronary angiography/coronary CT.

The exclusion criteria were: (i) history of angina pectoris caused by heart valve disease, coronary artery embolism, cardiomyopathy; (ii) combined with heart diseases, autonomic dysfunction, obvious anemia, obstructive emphysema, or electrolyte disorder; (iii) medication of digitalis.

HCs lacked typical history of angina pectoris or relevant symptoms and signs. Gut microbiota are highly susceptible to external stimuli, the composition and function of which can be influenced by the host’s metabolic changes associated with age, sex, genetic background, diet, living environment, exercise, and drugs. To minimize the influence of confounding factors on the gut microbiota and relevant metabolites, we adopted the strategy of propensity score matching to re-match subjects in the disease group and control group, based on the metabolic background of gut microbiota.

Blood samples were drawn after fasting for 12 h, and serum samples were prepared and immediately frozen at −80°C. The fecal samples were collected by the subjects using the MGIEasy stool sample collection kit (1000003702, MGI). Fecal Genomic DNA Extraction Kit (DP328, TIANGEN Biotech) was used for isolating metagenomic DNA of gut microbes.

### Metagenome sequencing, taxonomical, and functional annotation.

Metagenome sequencing was performed by Shanghai Majorbio Bio-pharm Technology Co. Ltd. DNA extract was fragmented to an average size of about 400 bp using Covaris M220 (Gene Company Limited) for paired-end library construction. Paired-end library was constructed using NEXTflexTM Rapid DNA-Seq (Bioo Scientific). Adapters containing the full complement of sequencing primer hybridization sites were ligated to the blunt-end of fragments. Paired-end sequencing was performed on Illumina NovaSeq (Illumina Inc.) at Majorbio Bio-Pharm Technology Co., Ltd. (Shanghai, China) using NovaSeq Reagent Kits according to the manufacturer’s instructions (www.illumina.com).

The KneadData (http://huttenhower.sph.harvard.edu/kneaddata, V.0.6) tool was used to ensure the data consisting of high-quality microbial reads free from contaminants. Low quality reads were removed using Trimmomatic (SLIDINGWINDOW:4:20 MINLEN:50 LEADING:3 TRAILING:3). The remaining reads were mapped to the human genome (hg38) by bowtie2 (V.2.3.5) (55) and the matching reads that were potentially host-associated and laboratory-associated sequences were removed as contaminant reads. Taxonomic profiling was performed using MetaPhlAn3 (v3.0), and only taxa detected in >10% of the number of samples were kept. Functional profiling (gene families, gene ontology, and pathways) was performed using HUMAnN3 (v3.0) with default settings.

To identify microbial genes of specific enzymes, hidden Markov models (HMMs) were constructed using HMMER (3.1b2) (56). Representative protein sequences of target enzymes were obtained from Uniprot database, and then high-quality sequences were selected and aligned in Clustal Omega (57). HMMs of enzymes were built on the sequences via hmmbuild in HMMER. Seed sequences from HMMs were realigned using hmmalign (default mode), and HMMs were rebuilt based on these alignments until both model length and relative entropy per position were constant. The constructed HMMs were used to screen specific enzymes from all microbial gene sequences via hmmsearch. Gene with e-value >10^−5^ were considered to express the enzymes.

### Targeted metabolomics profiling of serum samples.

A total of 25 SCAD patients and 12 HCs were randomly selected for targeted metabolomics analysis. All the serum samples were stored at −80°C until analyzed. We performed the targeted metabolomics analysis using the Q300 Kit (Metabo-Profile) using UPLC-QTOF-MS system (ACQUITY UPLC-Xevo TQ-S, Waters Corp.). All the standards of targeted metabolites were obtained from Sigma-Aldrich, Steraloids Inc., and TRC Chemicals. All the standards were accurately weighed and prepared in water, methanol, sodium hydroxide solution, or hydrochloric acid solution to obtain individual stock solution at a concentration of 5.0 mg/mL. Appropriate amount of each stock solution was mixed to create stock calibration solutions.

Serum sample aliquots of 25 μL was mixed with 120 μL ice-cold methanol with partial internal standards. After centrifugation, 30 μL supernatant was mixed with 20 μL freshly prepared derivative reagents and incubated at 30°C for 60 min. Then, 330 μL ice-cold 50% methanol solution was added, and the sample was stored for 20 min, followed by centrifugation at 4°C for 30 min. A total of 135 μL supernatant was mixed with 10 μL internal standards in new wells.

A UPLC-QTOF-MS system (ACQUITY UPLC-Xevo TQ-S, Waters Corp.) was used to quantitate the metabolites. ACQUITY UPLC BEH C18 1.7 μM analytical column (2.1 × 100 mm) was used for separation with the column temperature set at 40°C. The elution solvents were water with 0.1% formic acid (A) and acetonitrile/IPA (vol/vol = 70/30, B), with a flow rate of 400 μL/min. The gradient condition: 0 to 1 min (5% B), 1 to 11min (5 to 78% B), 11 to 13.5 min (78 to 95% B), 13.5 to 14 min (95 to 100% B), 14 to 16 min (100% B), 16 to 16.1 min (100 to 5% B), 16.1 to 18 min (5% B). The MS was operated at positive and negative electrospray ionization modes with a capillary voltage of 1.5 and 2.0 kV. The source temperature was set to 150°C, and the desolvation temperature was set to 550°C with a desolvation gas flow rate of 1000 L nitrogen per hour.

The raw data files generated by UPLC-MS/MS were processed using the QuanMET software (v2.0, Metabo-Profile) to perform peak integration, calibration, and quantitation for each metabolite. The relative abundance of metabolites was used for subsequent analysis.

### Construction of a quasi-paired cohort.

Metabolic background matching of metagenomic samples was performed to construct a quasi-paired cohort. The metabolic background of each individual was described by the abundance of microbial metabolic pathways. To extract the main metabolic information, principal-component analysis (PCA) was used to reduce the dimension of metabolic pathways. The principal components with cumulative explained variance >0.85 were retained for the propensity score calculation using logistic regression. Metabolic background was matched based on the propensity score by the nearest neighbor matching algorithm, and the optimal parameters (caliper and ratio) were determined through the covariate balance analysis using the standardized mean difference. “Caliper” defines the maximum distance of the propensity score between 2 samples, and “ratio” defines how many control samples could be matched to each disease sample. Finally, the optimal parameters (caliper = 0.25 and ratio = 2) (Fig. 1) of the PSM were determined through the covariate balance analysis. The above PSM was performed in the MatchIt (v4.3.2) package.

According to the above matching process, a total of 78 pairs of metagenome samples were obtained, including 28 unique healthy samples and 39 unique SCAD samples. A total of 34 pairs of metabolism samples were obtained, including 12 unique healthy samples and 25 unique SCAD samples (Fig. 1).

### Correlation analysis of gut microbial species, metabolites, and clinical characteristics.

For the quasi-paired cohort, a novel algorithm was utilized to evaluate the association of a change trend between gut microbial species, metabolites, and clinical characteristics. The signs of a change trend for each paired samples in the cohort were calculated, and then a normalized, mutual information score was used to evaluate the association of the change trends. Mutual information is the distance between 2 probability distributions based on entropy reduction, which could capture nonlinear relationships compared to linear correlation. Further, mutual information is helpful to mining causality in research, that is, information causality (58). Normalized mutual information score ranges from 0 to 1, indicating that the association between the 2 trends ranges from weak to strong. The sign of score was evaluated by the direction consistency of 2 trends. Permutation test (1000 permutations) was performed to determine the *P*-value of correlation.

### Disease diagnosis model based on metagenome and metabolome.

The machine-learning procedure, random forest classifier in the Scikit-learn package of Python (3.6.0) was used to predict phenotypes based on the metagenome and metabolome data. The AUC of 5-fold cross-validation was utilized to measure the discriminative ability of the model. The hyperparameters of the model were optimized by grid-search over a parameter grid. The hyperparameters with the highest AUC were used in subsequent modeling. The best features were selected by recursive feature elimination. Finally, the best model was determined by the optimal hyperparameters and features.

### qPCR validation.

To validate the relative abundance of R. pickettii between the SCAD patients and HCs, qPCR analysis was performed in the matching cohort. DNA from R. pickettii (ATCC27511, purchased from BeNa Culture Collect) was used to produce standard curves for relative quantification using a forward primer (5′-ATGATCTAGCTTGCTAGATTGAT-3′) and reverse primer (5′-ACTGATCGTCGCCTTGGTG-3′) (59). The adopted qPCR program was as follows: pre-denaturation at 95°C for 2 min; denaturation at 95°C for 15 s and annealing at 60°C for 60 s for 40 cycles, followed by a melt curve analysis. The relative abundance of R. pickettii was obtained by dividing the concentration of R. pickettii by the total DNA concentration of fecal bacteria based on the standard curve and CT values of each sample.

### Targeted metabolomics validation with an independent cohort.

As an external test, we used additional independent data to validate the changes of unsaturated fatty acid in SCAD patients. These clinical samples were collected at the same time as the main cohort. A total of 30 SCAD patients and 30 HCs were randomly selected for targeted metabolomics analysis.

### The relationship of unsaturated fatty acid and R. pickettii validation.

We cocultured R. pickettii (ATCC27511, purchased from BeNa Culture Collection) with sodium oleate (300 μM) and sodium linoleate (800 μM) for 48 h. The bacterial solution was collected at the time points of 0 h, 6 h, 12 h, 24 h, and 48 h, and were centrifuged at 12000 rpm for 2 min (*n* = 3). The OD values of the supernatant were detected at 600 nm to observe the effect of unsaturated fatty acid on the growth of R. pickettii. The other part of the supernatant was used to detect the changes of oleic acid and linoleic acid content by Targeted metabolomics.

### Bioinformatics and statistical analysis.

Statistical significance was determined by the one-sided Fisher's exact test, two-sided Wilcoxon rank-sum test, or permutation test where appropriate. When not specified otherwise, the statistical analyses were performed with Python (3.6.0) and referenced in the description of the analyses. Wilcoxon signed-rank test was applied to compare the difference between paired samples. False-discovery rate (FDR) was calculated according to Benjamini-Hochberg under multiple comparisons. Differences were considered statistically significant when FDR < 0.05. Alpha diversity of metagenomics was measured by Chao1 index, and beta diversity was measured by Bray-Curtis distance and unweighted unifrac distance in the scikit-bio (v0.5.6) package. Distribution of metabolites between different groups was displayed using the opls-da in ropls (v 1.24.0) package. Functional enrichment analysis was performed for microbial genes and metabolites by hypergeometric test in self-build codes, clusterProfiler (v4.2.1), or MetaboAnalyst (v5.0). All networks were constructed and visualized in Cystoscape (v3.9.0). Comparative genomic analysis of the metabolic process and enzymes were analyzed through the genome-scale metabolic models obtained from AGORA (60). Enzymes were annotated by the ENZYME (61) and BRENDA (62) databases.

### Data availability.

All data can be viewed in NODE (https://www.biosino.org/node) by pasting the accession OEP003421 into the text search box or through the URL: https://www.biosino.org/node/project/detail/OEP003421. Data are available upon reasonable request. All the software packages used in this study are open source and publicly available, and the code used in this study is available at GitHub at https://github.com/ddhmed/SCAD2022.
