# Supplementary material for: The Microbial and Metabolic Signatures of Patients with Stable Coronary Artery Disease
Source: Microbiol Spectr. 2022 Nov 10;10(6):e02467-22. doi: 10.1128/spectrum.02467-22 (PMC9769616; doi:10.1128/spectrum.02467-22)
Supplement: Supplemental file 1 — Supplemental material. Download spectrum.02467-22-s0001.pdf, PDF file, 2.3 MB [file spectrum.02467-22-s0001.pdf]

Supplementary materials

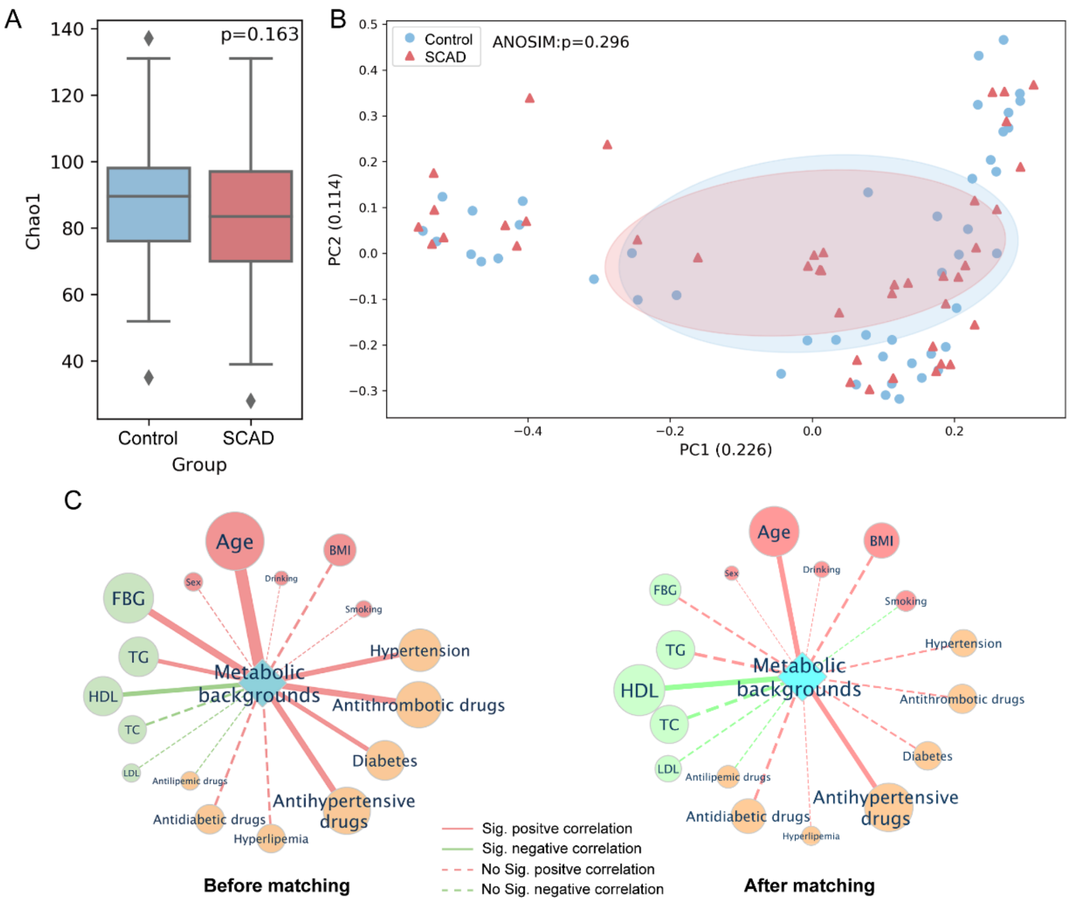

Figure S1 Individual differences confuse the differences between HCs and SCAD patients. (A) (B) Alpha-diversity, beta-diversity of gut microbiome showed no alteration between HCs and SCAD patients before PSM. Wilcoxon rank-sum test and Analysis of Similarities (ANOSIM) was used to determine the significance. (C) Correlation of demographics and clinical characteristics with metabolic background before and after PSM. The size of circles and thickness of links indicated the r value of Spearman correlation.

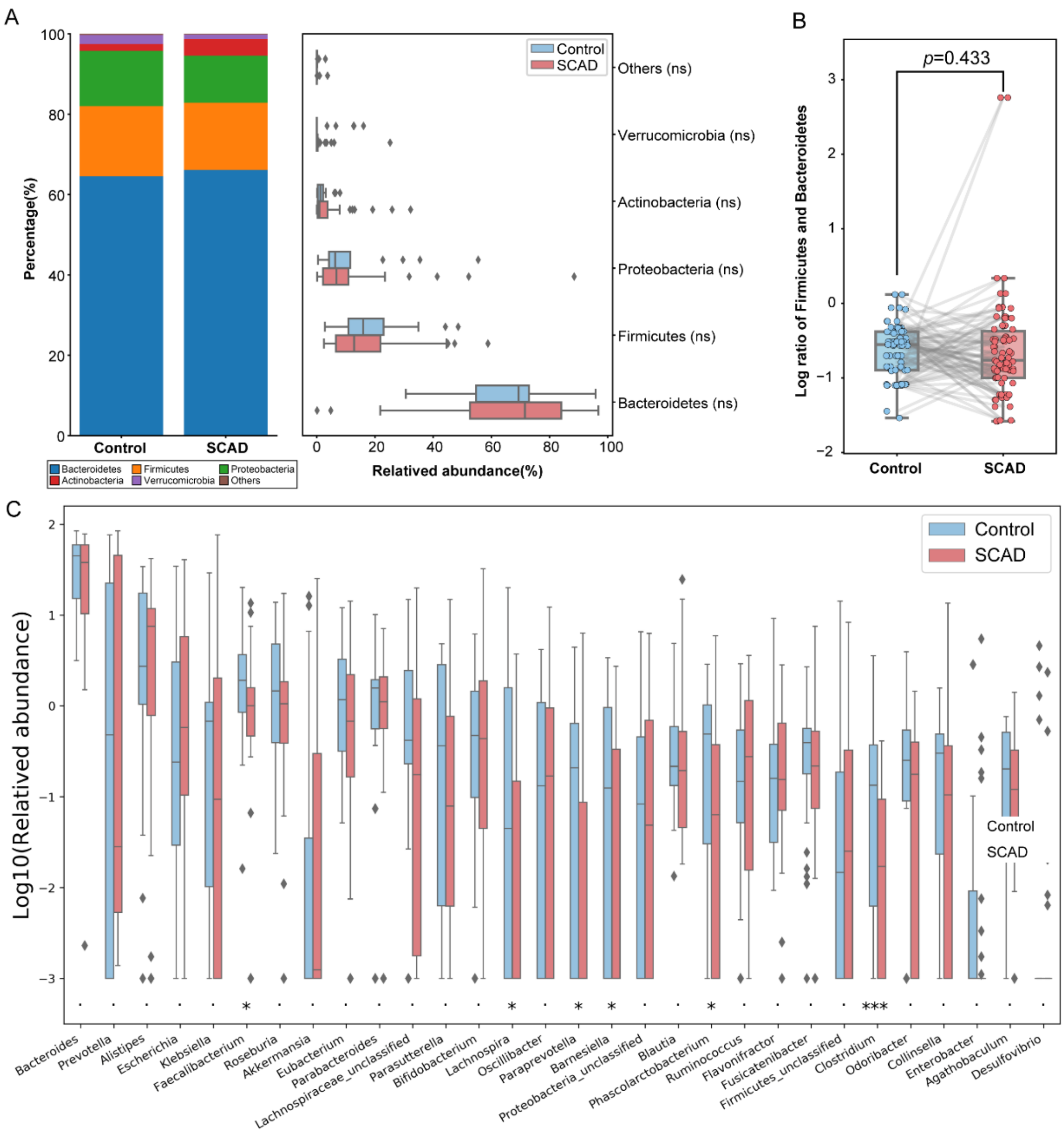

Figure S2 Gut microbiome variations at the phylum and genus level in the cohort. (A) The proportion and relative abundance of the five most abundant phylum in the two groups. (B) The ratio of *Bacteroidetes* to *Firmicutes* between the two groups. (C) The relative abundance of the thirty most abundant genera in the two groups. Wilcoxon signed-rank test was used to determine the significance. **P*<0.05, ****P*<0.001.

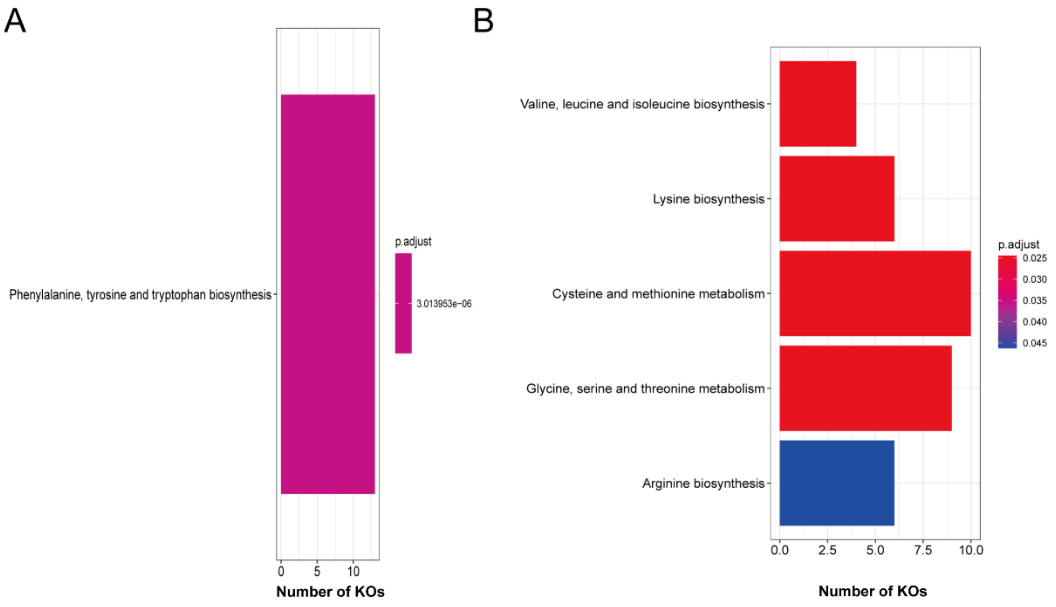

Figure S3 The different amino acid metabolic pathways enriched by up-regulated (A) and down-regulated (B) KOs in SCAD.

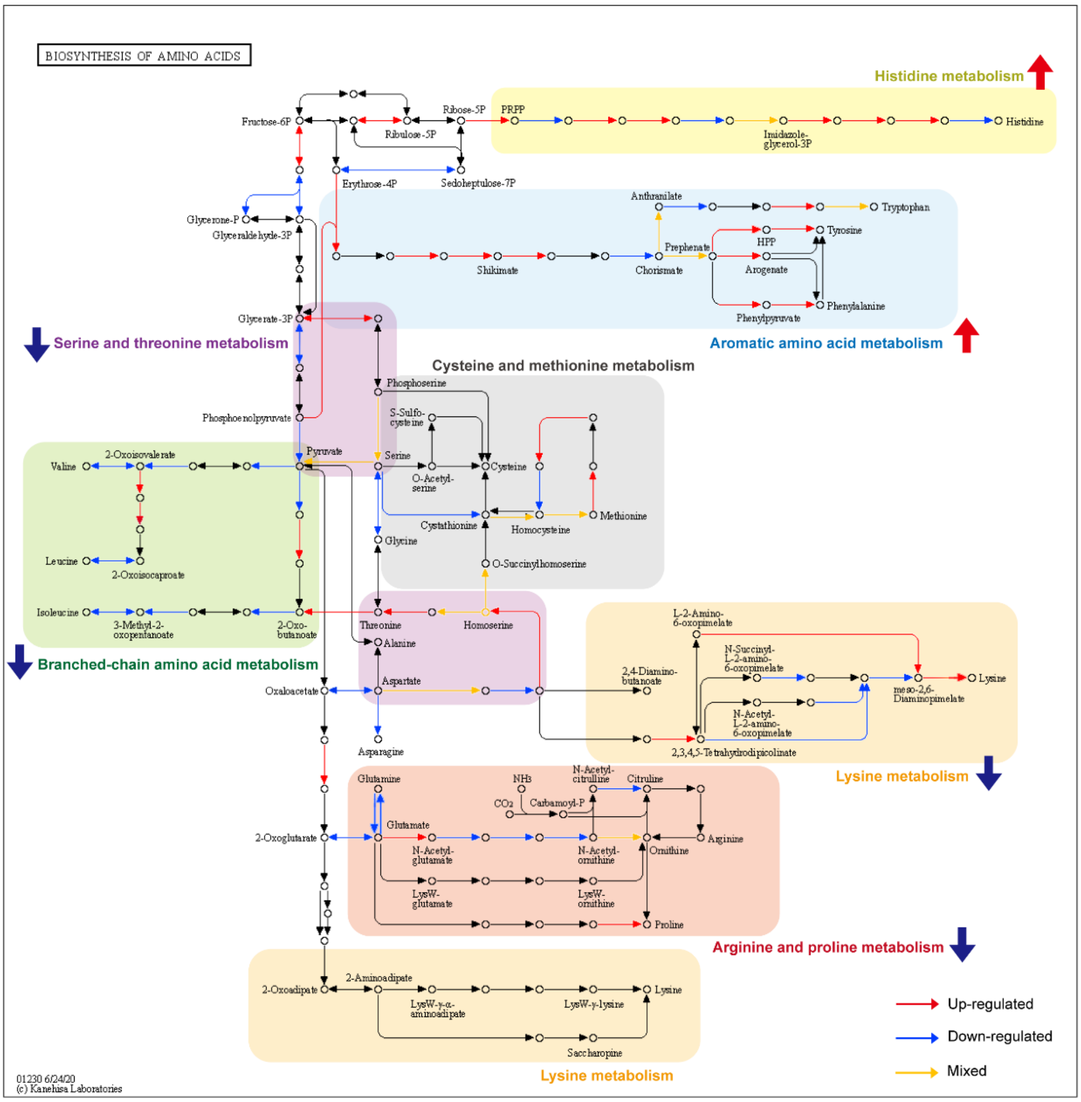

Figure S4 Key amino acid metabolic pathways mapped by microbial differential KO genes. The pathways were generated on the basis of KEGG pathway maps. Blue arrows represent down-regulated KOs or pathways, red arrows represent up-regulated KOs or pathways, yellow arrows indicate the trend is unclear, which mixed up and down regulated KOs in SCAD patients.

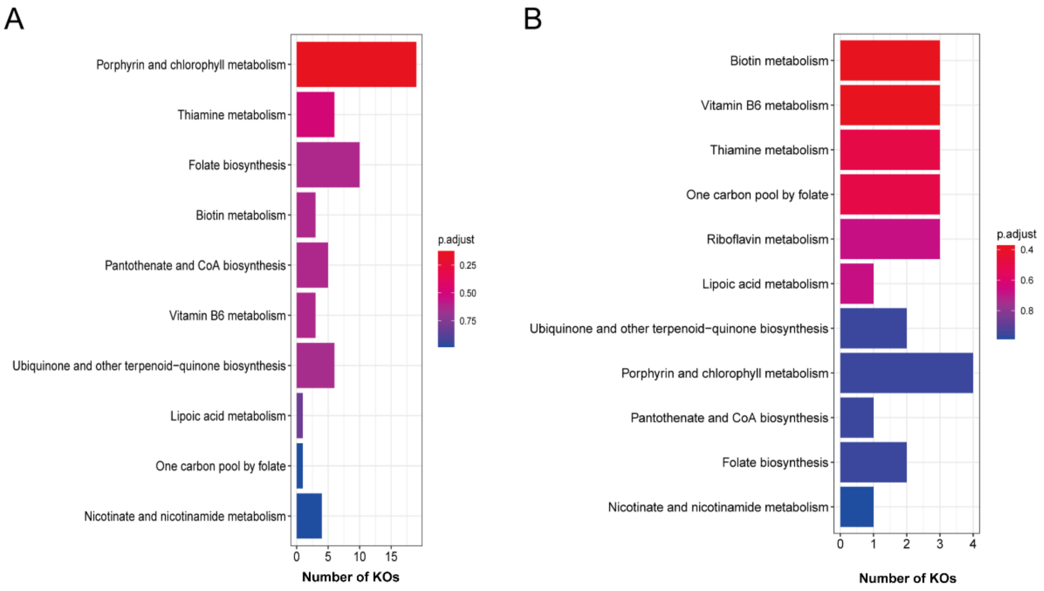

Figure S5 The different cofactor metabolic pathways enriched by up-regulated (A) and down-regulated (B) KOs in SCAD.

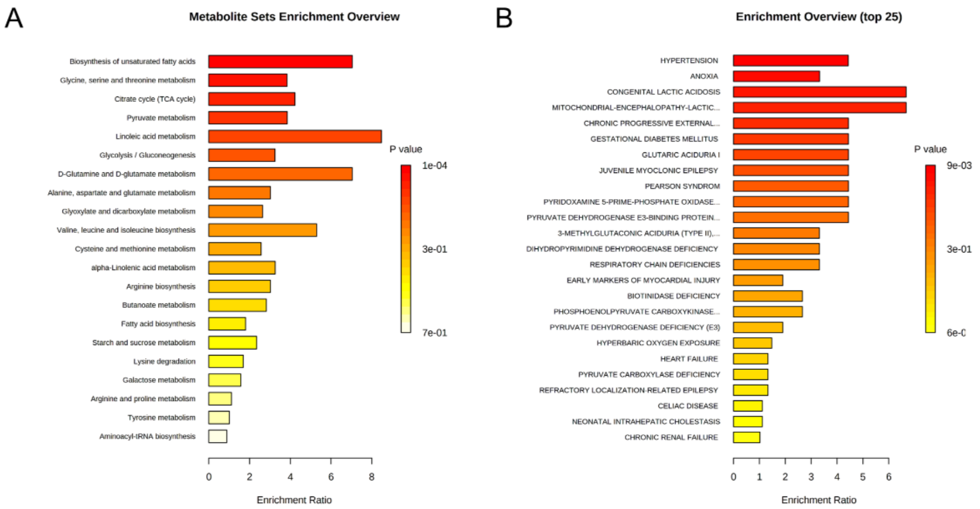

Figure S6 The metabolic pathway (A) and the human disease (B) enrichment analysis of differential serum metabolites in SCAD by MetaboAnalyst.

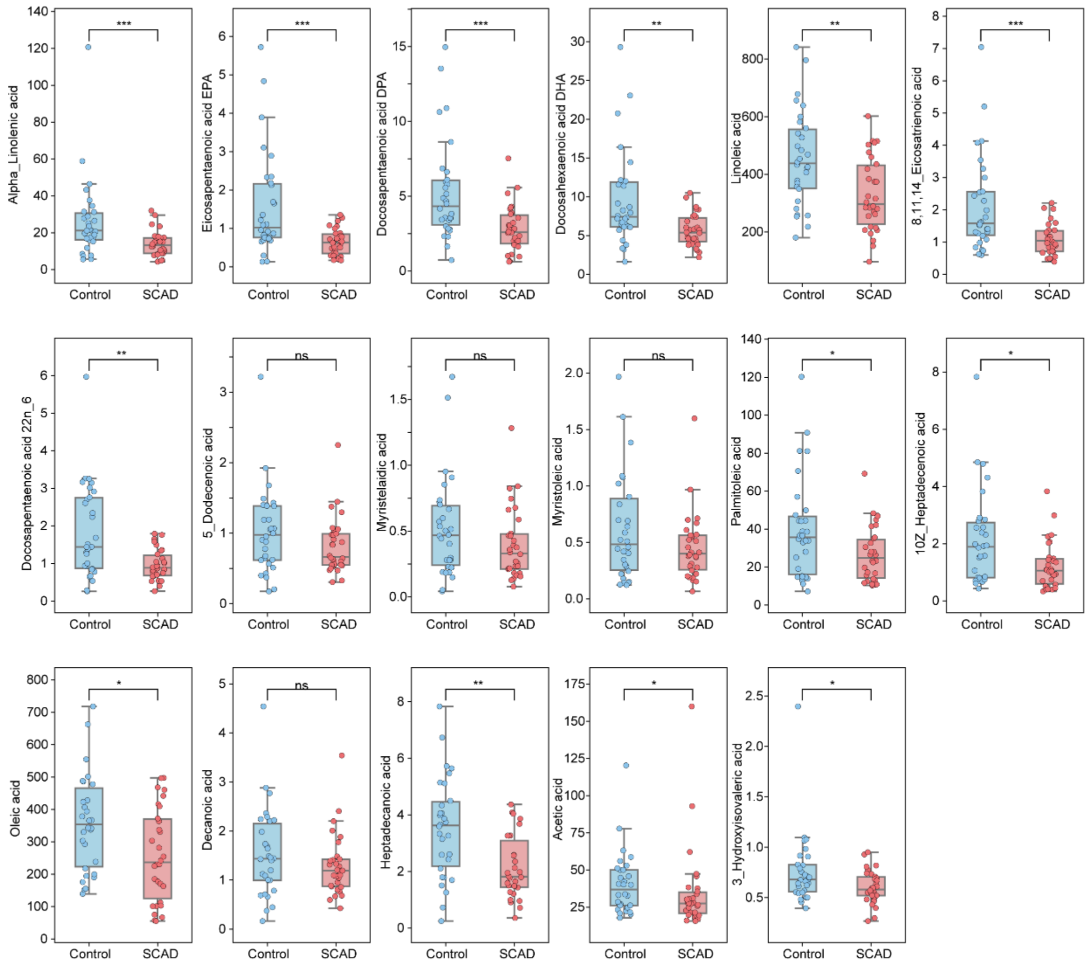

Figure S7 The relative abundances of unsaturated fatty acid between SCAD patients (n=30) and HCs (n=30) in an independent cohort. Wilcoxon ranksum test was used to determine the significance. **P*<0.05, ***P*<0.01 ****P*<0.001.

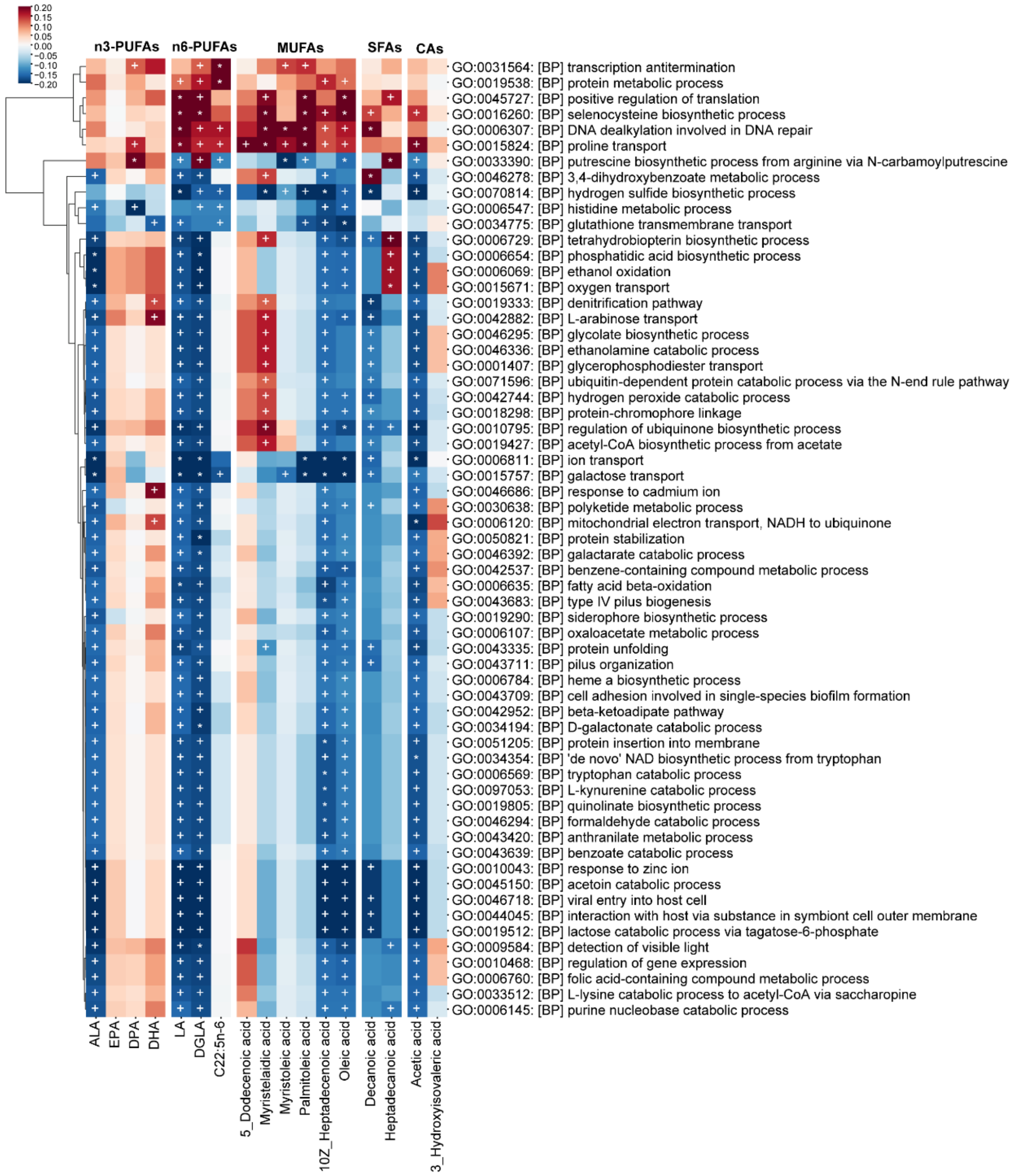

Figure S8 The key GO terms (biological process) correlated to fatty acids. The signs of change trend for each paired samples in cohort were calculated, and then normalized mutual information score was used to evaluate the association of change trends. Normalized mutual information score ranges from 0 to 1, indicating that the association between the two trends ranges from weak to strong. The sign of score was evaluated by the direction consistency of two trends. Permutation test (1000 permutations) was performed to determine p-value of correlation and GO term significant correlated to >5 fatty acids was selected for display. * and + suggest the significance, + *P*<0.05, * *P*<0.01.

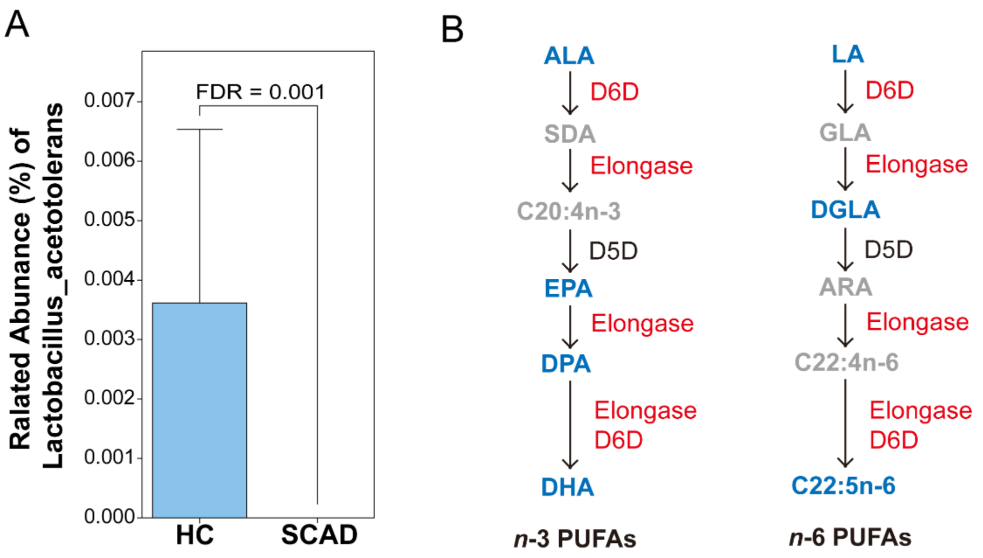

Figure S9 The abundance of microbial species and enzyme for metabolizing unsaturated fatty acid in SCAD. (A) Relative abundance of *Lactobacillus acetotolerans* between the two groups. Wilcoxon signed-rank test, $P_{\text{FDR}}<0.001$. (B) Metabolism of alpha_linolenic acid (ALA) and alpha_linoleic acid (LA) by gut microbes. Blue represents the unsaturated fatty acids decreased significantly in SCAD patients. Red represents the relative abundance of metabolic enzyme for unsaturated fatty acids increased significantly in SCAD. SDA: Stearidonic acid; EPA: Eicosapentaenoic acid; DPA: Docosapentaenoic acid; DHA: Docosahexaenoic acid; GLA: Gamma-linolenic acid; DGLA: Dihomo-gamma-linolenic acid; ARA: Arachidonic acid; D6D: Delta-6 desaturase, D5D: Delta-5 desaturase.

Table S1 The characteristics of the study population before PSM.

Feature	Control(46)	SCAD(42)	p-value
Age,years	58.174 (55.454, 60.894)	66.452 (63.897, 69.007)	0.000137469
Sex	20 (43.478%)	15 (35.714%)	0.516917767
BMI,kg/m^2	23.507 (22.606, 24.408)	24.217 (23.098, 25.336)	0.213221628
Smoking	12 (26.087%)	10 (23.81%)	1
Drinking	14 (30.435%)	9 (21.429%)	0.466816253
Hypertension	13 (28.261%)	28 (66.667%)	0.000543693
Diabetes	6 (13.043%)	17 (40.476%)	0.004008625
Hyperlipemia	10 (21.739%)	22 (52.381%)	0.003879397
Antilipemic drugs	3 (6.522%)	5 (11.905%)	0.471014552
Antihypertensive drugs	6 (13.043%)	13 (30.952%)	0.067868752
Antidiabetic drugs	2 (4.348%)	3 (7.143%)	0.666375772
Antithrombotic drugs	1 (2.174%)	15 (35.714%)	3.94E-05
TG,mmol/l	1.205 (1.0, 1.411)	1.52 (1.303, 1.738)	0.003143386
TC,mmol/l	4.961 (4.663, 5.26)	4.551 (4.232, 4.87)	0.139206085
HDL-C,mmol/l	1.434 (1.33, 1.538)	1.32 (1.228, 1.412)	0.030147511
LDL-C,mmol/l	2.941 (2.673, 3.208)	2.632 (2.373, 2.891)	0.277451658
FBG,mmol/l	5.711 (5.288, 6.134)	6.872 (6.235, 7.51)	0.00022943
HCY	13.774 (11.735, 15.812)	14.948 (13.116, 16.779)	0.354341742
CRP	1.411 (0.918, 1.904)	1.926 (1.263, 2.589)	0.49821985

Table S2 The metabolic pathways of gut microbiome showed no alteration between HCs and SCAD patients before PSM.

Pathway	Normal_mean	CAD_mean	FC	p_value	FDR
PWY-6703: preQ0 biosynthesis	0.007232951	0.005477955	0.757361046	0.0014583	0.250224435
HSERMETANA-PWY: L-methionine biosynthesis III	0.005051828	0.003583221	0.709291971	0.002170152	0.250224435
PWY-6163: chorismate biosynthesis from 3-dehydroquinate	0.010256084	0.008630511	0.841501593	0.00229457	0.250224435
PWY-3781: aerobic respiration I (cytochrome c)	0.000232386	0.000259634	1.117255747	0.002708057	0.250224435
1CMET2-PWY: N10-formyl-tetrahydrofolate biosynthesis	0.009680658	0.008401525	0.867867208	0.010327583	0.328487829
PWY-5973: cis-vaccenate biosynthesis	0.007778353	0.006815503	0.876214162	0.011918777	0.328487829
PWY-6531: mannitol cycle	0.000489463	0.000651055	1.330141076	0.012793077	0.328487829
PWY-7234: inosine-5'-phosphate biosynthesis III	0.002914636	0.003750303	1.286713753	0.014046878	0.328487829
PWY0-1261: anhydromuropeptides recycling	0.00302615	0.003575844	1.181647902	0.014376795	0.328487829
PANTOSYN-PWY: pantothenate and coenzyme A biosynthesis I	0.00922876	0.008127397	0.880659668	0.017271488	0.328487829
PWY-7229: superpathway of adenosine nucleotides de novo biosynthesis I	0.008229967	0.007544152	0.916668563	0.020211456	0.328487829
TCA: TCA cycle I (prokaryotic)	0.001454751	0.002034833	1.398749962	0.021127894	0.328487829
PWY-6126: superpathway of adenosine nucleotides de novo biosynthesis II	0.007425054	0.006715047	0.904376881	0.021599539	0.328487829
PWY-5690: TCA cycle II (plants and fungi)	0.000752812	0.001067326	1.417786034	0.025165055	0.328487829
COA-PWY-1: coenzyme A biosynthesis II (mammalian)	0.010839875	0.009491298	0.875591141	0.026842698	0.328487829
COA-PWY: coenzyme A biosynthesis I	0.010487931	0.009063179	0.86415322	0.026842698	0.328487829
PWY-6125: superpathway of guanosine nucleotides de novo biosynthesis II	0.006514189	0.005375971	0.825270898	0.026842698	0.328487829
PWY-7221: guanosine ribonucleotides de novo biosynthesis	0.011907983	0.010331961	0.867649991	0.026842698	0.328487829
FERMENTATION-PWY: mixed acid fermentation	0.001018946	0.001514003	1.485851534	0.028014034	0.328487829
PWY-7228: superpathway of guanosine nucleotides de novo biosynthesis I	0.006872096	0.005656385	0.823094548	0.028616045	0.328487829
PWY-7663: gondoate biosynthesis (anaerobic)	0.007138291	0.006420566	0.899454202	0.029229165	0.328487829
FASYN-INITIAL-PWY: superpathway of fatty acid biosynthesis initiation (E. coli)	0.002525776	0.003237428	1.281755997	0.031795983	0.328487829
PWY-7222: guanosine deoxyribonucleotides de novo biosynthesis II	0.006148659	0.005351731	0.870389932	0.036007533	0.328487829
PWY-7220: adenosine deoxyribonucleotides de novo biosynthesis II	0.006148659	0.005351731	0.870389932	0.036007533	0.328487829

PWY-6282: palmitoleate biosynthesis I (from (5Z)-dodec-5-enoate)	0.002649117	0.003295799	1.24411236	0.036753801	0.328487829
PWY-5695: urate biosynthesis/inosine 5'-phosphate degradation	0.010805133	0.00963035	0.891275445	0.037513203	0.328487829
PWY-6969: TCA cycle V (2-oxoglutarate:ferredoxin oxidoreductase)	0.000851151	0.001143597	1.343588898	0.037513203	0.328487829
PWY0-1479: tRNA processing	0.001542104	0.001946264	1.262083331	0.038285917	0.328487829
P461-PWY: hexitol fermentation to lactate, formate, ethanol and acetate	0.001013992	0.001424673	1.405013181	0.039871994	0.328487829
FASYN-ELONG-PWY: fatty acid elongation -- saturated	0.003058435	0.003711046	1.213380653	0.039871994	0.328487829
PWY-5989: stearate biosynthesis II (bacteria and plants)	0.002555086	0.003117938	1.220286878	0.039871994	0.328487829
PWY-5676: acetyl-CoA fermentation to butanoate II	0.000594567	0.000769594	1.294375922	0.041513476	0.328487829
PWY-5188: tetrapyrrole biosynthesis I (from glutamate)	0.001636502	0.002027769	1.239087235	0.042355449	0.328487829
PWY-7664: oleate biosynthesis IV (anaerobic)	0.002863673	0.003499989	1.2222027	0.042355449	0.328487829
PWYG-321: mycolate biosynthesis	0.00295927	0.003633113	1.227705908	0.042355449	0.328487829
DENOVOPURINE2-PWY: superpathway of purine nucleotides de novo biosynthesis II	0.006201834	0.005595924	0.902301494	0.043211823	0.328487829
PWY0-862: (5Z)-dodec-5-enoate biosynthesis	0.002645592	0.003257484	1.231287403	0.043211823	0.328487829
PWY-4041: γ-glutamyl cycle	0.001328639	0.001679782	1.264287214	0.044082782	0.328487829
PWY-6823: molybdenum cofactor biosynthesis	0.00013027	0.000176581	1.355504583	0.046325221	0.331088229
PWY-7388: octanoyl-[acyl-carrier protein] biosynthesis (mitochondria, yeast)	0.00262626	0.003278302	1.248277928	0.046785047	0.331088229
PWY-6215: 4-chlorobenzoate degradation	1.72203E-05	1.77603E-05	1.031359919	0.047248708	0.331088229
PWY-6519: 8-amino-7-oxononanoate biosynthesis I	0.002735855	0.003294625	1.204239652	0.049625367	0.331088229
P441-PWY: superpathway of N-acetylneuraminate degradation	0.001183629	0.001638798	1.384553603	0.049625367	0.331088229

Table S3 The characteristics of the study population after PSM.

Feature	Control(78)	SCAD(78)	p-value
Age,years	62.103 (59.914, 64.291)	66.462 (64.601, 68.322)	0.005073852
Sex	37 (47.436%)	26 (33.333%)	0.102389684
BMI,kg/m2	23.312 (22.681, 23.942)	24.387 (23.585, 25.189)	0.04716341
Smoking	22 (28.205%)	18 (23.077%)	0.582606188
Drinking	31 (39.744%)	18 (23.077%)	0.037901121
Hypertension	23 (29.487%)	54 (69.231%)	1.15E-06
Diabetes	9 (11.538%)	32 (41.026%)	4.52E-05
Hyperlipemia	15 (19.231%)	42 (53.846%)	1.18E-05
Antilipemic drugs	9 (11.538%)	10 (12.821%)	1
Antihypertensive drugs	9 (11.538%)	24 (30.769%)	0.00551813
Antidiabetic drugs	0 (0.0%)	6 (7.692%)	0.028284973
Antithrombotic drugs	5 (6.41%)	26 (33.333%)	3.48E-05
TG,mmol/l	1.137 (1.036, 1.238)	1.527 (1.366, 1.688)	0.000232484
TC,mmol/l	5.139 (4.893, 5.384)	4.574 (4.351, 4.796)	0.006537585
HDL-C,mmol/l	1.447 (1.364, 1.531)	1.337 (1.272, 1.402)	0.016207389
LDL-C,mmol/l	3.114 (2.892, 3.336)	2.636 (2.451, 2.821)	0.01528136
FBG,mmol/l	5.997 (5.683, 6.311)	6.944 (6.473, 7.415)	0.003866317
HCY	15.05 (13.77, 16.33)	15.228 (13.881, 16.575)	0.97014804
CRP	1.503 (1.2, 1.807)	1.911 (1.436, 2.387)	0.563837872

Table S4 The genera differentially abundant between SCAD patients and HCs.

Genus	Normal_mean	SCAD_mean	FC	p_value	FDR
Rothia	0.009939359	0.001727179	0.173771718	4.96E-09	7.94E-07
Clostridium	0.408369744	0.06456641	0.158107723	9.36E-07	7.48E-05
Actinomyces	0.055873718	0.016315385	0.292004635	1.76E-06	9.41E-05
Ralstonia	0.031191923	0.096620256	3.097604985	8.34E-05	0.003335741
Leuconostoc	0.000640256	6.92E-06	0.010812976	0.000436135	0.012447954
Paraprevotella	0.715396154	0.389663333	0.544681896	0.000490948	0.012447954
Clostridiales_Family_XIII_Incertae_Sedis_unclassified	0.001356282	0	0	0.000553718	0.012447954
Asaccharobacter	0.026270769	0.025449744	0.96874756	0.000686389	0.012447954
Anaeromassilibacillus	0.001659231	0.000253333	0.152681193	0.000700197	0.012447954
Barnesiella	0.621885897	0.239777692	0.385565412	0.001066393	0.017062292
Adlercreutzia	0.076323462	0.074883846	0.98113797	0.00136076	0.019792876
Phascolarctobacterium	0.58907	0.432380769	0.734005754	0.001747526	0.021739528
Peptoniphilus	0.001596667	0	0	0.001766337	0.021739528
Peptococcus	0.000531923	0	0	0.002699796	0.026997961
Varibaculum	6.23E-05	0	0	0.002699796	0.026997961
Tissierellia_unclassified	0.000333462	0	0	0.002699796	0.026997961
Holdemanella	0.027072436	0.002506923	0.092600573	0.002919021	0.027473138
Cloacibacillus	0.004181026	3.08E-05	0.007359254	0.003983112	0.033873519
Faecalibacterium	2.860007436	1.907406923	0.666923764	0.00402248	0.033873519
Anaerococcus	0.001712308	2.54E-05	0.014824798	0.004473649	0.035397356
Enterococcus	0.000174872	0.007477179	42.75806452	0.004645903	0.035397356
Lachnospira	1.050570897	0.327135385	0.311388204	0.00543029	0.038331391
Methanosphaera	0.00139	0	0	0.005749709	0.038331391
Sanguibacteroides	0.002127308	0	0	0.005749709	0.038331391

Megasphaera	0.009825513	0.524394615	53.37071204	0.007351906	0.047052196
Pseudoflavonifractor	0.001350641	0	0	0.008150972	0.048302054
Desulfovibrionaceae_unclassified	0.008865769	0	0	0.008150972	0.048302054

Table S5 The species differentially abundant between SCAD patients and HCs.

Species	Normal_mean	SCAD_mean	FC	p_value	FDR
Actinomyces_odontolyticus	0.005934615	0.001166923	0.196629942	2.50E-07	2.36E-05
Actinomyces_sp_HMSC035G02	0.001993205	0.000204103	0.102399177	2.31E-05	0.001091146
Actinomyces_sp_ICM47	0.037781282	0.007194615	0.190428037	9.17E-06	0.000577762
Actinomyces_sp_S6_Spd3	0.000756667	6.95E-05	0.091833277	0.00555138	0.031026186
Bifidobacterium_dentium	0.030114872	0.487255128	16.17988386	0.004882681	0.03076089
Rothia_mucilaginosa	0.009822308	0.001370513	0.139530634	2.52E-09	4.76E-07
Bacteroides_clarus	0.011053718	0.225216667	20.37474339	0.00110657	0.010457086
Bacteroides_massiliensis	0.330961026	0.109498205	0.330849244	9.70E-05	0.001833716
Bacteroides_nordii	0.000222564	0.002775641	12.47119816	0.000492585	0.006999948
Barnesiella_intestinihominis	0.621885897	0.239777692	0.385565412	0.001066393	0.010457086
Paraprevotella_xylaniphila	0.616380385	0.337727692	0.547920896	0.000189832	0.003261667
Prevotella_timonensis	0.017463462	0.006530256	0.373938259	0.000231731	0.003649765
Parabacteroides_merdae	0.966547179	0.354800769	0.367080652	2.89E-05	0.001094267
Gemella_sanguinis	0.001779231	0.000198462	0.11154345	0.000518515	0.006999948
Lactobacillus_acetotolerans	0.00489141	0	0	3.68E-05	0.001158724
Streptococcus_mitis	0.004259872	9.41E-05	0.022090469	7.84E-05	0.001751264
Streptococcus_oralis	0.001336795	0	0	0.002106265	0.017308002
Clostridium_sp_CAG_242	0.022316538	0.001890256	0.084702043	0.00731266	0.039488366
Clostridium_sp_CAG_58	0.126487179	0.03132359	0.247642408	0.000786546	0.008258737
Eubacterium_ramulus	0.045735513	0.021014359	0.459475748	0.000616375	0.00776633
Eubacterium_ventriosum	0.001425128	0.009101795	6.386649874	0.001409272	0.01210693
Ruminococcus_torques	0.09703141	0.036453333	0.375685907	7.99E-05	0.001751264
Lachnospira_pectinoschiza	1.050570897	0.327135385	0.311388204	0.00543029	0.031026186

Roseburia_hominis	0.141373333	0.449818718	3.181779105	0.003324635	0.024167542
Anaeromassilibacillus_sp_An250	0.001659231	0.000253333	0.152681193	0.000700197	0.007784548
Holdemanella_biformis	0.027072436	0.002506923	0.092600573	0.002919021	0.02269672
Firmicutes_bacterium_CAG_83	0.038350256	0.271390769	7.076635064	0.005442185	0.031026186
Phascolarctobacterium_faecium	0.473231282	0.280537436	0.592812535	0.00558143	0.031026186
Megasphaera_elsdenii	0.00945359	0.520682564	55.07776181	0.004744444	0.03076089
Ralstonia_pickettii	0.031191923	0.096620256	3.097604985	8.34E-05	0.001751264

Table S6 The differential KOs of gut microbes between SCAD patients and HCs.

KO	Normal_mean	SCAD_mean	FC	p_value	FDR
K01610	0.004679962	0.002573922	0.549987805	1.81E-09	1.96E-06
K05808	0.000167167	0.000833879	4.988298054	1.97E-09	1.96E-06
K00789	0.000413906	0.001116522	2.69752442	6.02E-09	3.99E-06
K12573	0.002225046	0.000726909	0.326693768	1.23E-08	6.12E-06
K01890	0.000342993	0.001029091	3.000325217	2.51E-08	9.99E-06
K00174	0.002421949	0.000606131	0.250265986	3.57E-08	1.18E-05
K03060	0.000423878	0.001233571	2.910205872	9.53E-08	2.60E-05
K00951	0.001718506	0.000596422	0.347058068	1.04E-07	2.60E-05
K00820	0.00042584	0.001393546	3.272466823	1.21E-07	2.68E-05
K03524	0.000171569	0.000642347	3.743952838	2.03E-07	3.66E-05
K00610	0.001116096	0.000243344	0.218031105	2.30E-07	3.66E-05
K02952	0.004430218	0.002272277	0.512904035	2.31E-07	3.66E-05
K00997	5.28E-05	0.000428287	8.113700941	2.39E-07	3.66E-05
K01935	1.80E-05	0.000350234	19.44307935	3.93E-07	5.58E-05
K03979	0.003876106	0.002093715	0.540159392	5.64E-07	6.49E-05
K03585	0.001209958	0.000510704	0.422083946	5.72E-07	6.49E-05
K03215	0.000166029	0.000607222	3.657318403	5.85E-07	6.49E-05
K01710	0.001030075	0.000347051	0.336918303	5.87E-07	6.49E-05
K00525	0.000115121	0.000787486	6.840523557	6.38E-07	6.69E-05
K00873	0.001659959	0.000506198	0.304946057	6.74E-07	6.71E-05
K01866	0.003285313	0.001935476	0.589129781	7.48E-07	6.89E-05
K02012	8.22E-05	0.0005812	7.068971025	7.62E-07	6.89E-05
K07126	0.000139757	0.001130935	8.09216932	8.17E-07	6.91E-05
K18332	0.000946917	0.000235905	0.249129655	8.33E-07	6.91E-05

K01745	7.93E-05	0.000611971	7.719134279	1.07E-06	8.50E-05
K02016	0.000352295	0.000885035	2.512198083	1.20E-06	9.15E-05
K04759	0.000337106	0.000735927	2.18307215	1.32E-06	9.15E-05
K00239	0.000236628	0.000907738	3.836132312	1.41E-06	9.15E-05
K06901	0.00023488	0.000643294	2.738824585	1.43E-06	9.15E-05
K03499	0.002615031	0.001207184	0.461632752	1.50E-06	9.15E-05
K01889	0.002726072	0.001380955	0.506573294	1.52E-06	9.15E-05
K18968	5.75E-05	0.000583088	10.13257468	1.53E-06	9.15E-05
K00826	0.001887899	0.001042702	0.552308008	1.54E-06	9.15E-05
K02005	0.003815066	0.000331388	0.086862987	1.57E-06	9.15E-05
K00919	3.17E-05	0.000337498	10.65575287	1.63E-06	9.15E-05
K01611	7.54E-05	0.000520828	6.908801491	1.66E-06	9.15E-05
K00962	0.00384042	0.002275258	0.592450391	1.76E-06	9.47E-05
K03070	0.004142592	0.002134724	0.515311169	1.90E-06	9.93E-05
K03527	0.003975518	0.001474048	0.37078122	2.01E-06	0.000102346
K03595	0.001252809	0.000528666	0.421984628	2.17E-06	0.000107889
K02911	0.007763418	0.004741498	0.6107488	2.31E-06	0.000111903
K02050	0.000265319	0.000874582	3.296346895	2.57E-06	0.000116969
K01809	0.00090827	0.000293927	0.323611778	2.58E-06	0.000116969
K08296	6.62E-05	0.000519428	7.851004909	2.64E-06	0.000116969
K02042	0.000177921	0.000571599	3.212662549	2.65E-06	0.000116969
K03183	0.002698478	0.000891794	0.330480231	2.79E-06	0.000120325
K03294	0.001110695	0.000343473	0.30924117	2.84E-06	0.000120325
K00912	2.28E-05	0.000380078	16.63523345	2.98E-06	0.000121877
K06871	0.000691218	0.000189355	0.273944025	3.00E-06	0.000121877
K00342	0.001158844	0.000294878	0.254459147	3.32E-06	0.000131968

K01091	0.000580028	0.001207689	2.082120334	3.50E-06	0.000136663
K02954	0.005620878	0.003123875	0.555762856	4.23E-06	0.000158843
K00566	0.00300933	0.001707846	0.567516822	4.23E-06	0.000158843
K02342	7.14E-05	0.000516946	7.235875267	4.34E-06	0.000159888
K07727	0.001457192	0.000317895	0.218156049	4.53E-06	0.000163608
K02238	7.70E-05	0.000361332	4.693745666	4.62E-06	0.000163608
K11645	0.001072572	0.000303879	0.283317885	4.69E-06	0.000163608
K06168	0.003131221	0.001804044	0.576146923	4.82E-06	0.000165528
K01997	0.000329461	0.000770023	2.337221629	5.32E-06	0.000171837
K02564	0.003384704	0.001883847	0.556576497	5.36E-06	0.000171837
K08325	0.001005967	0.000274653	0.273023344	5.43E-06	0.000171837
K06942	0.00109388	0.000314224	0.287256166	5.43E-06	0.000171837
K16899	0.000247604	0.000654251	2.642328352	5.44E-06	0.000171837
K19286	0.002181321	0.000357027	0.163674513	5.67E-06	0.000174441
K00346	0.000662513	0.00018994	0.286696495	5.70E-06	0.000174441
K00347	0.001926415	0.000848308	0.440355641	5.80E-06	0.000175018
K01649	0.000149319	0.000623654	4.17666551	6.29E-06	0.00018338
K01754	0.00070331	0.001244269	1.769160506	6.33E-06	0.00018338
K03076	0.001227118	0.000425267	0.346557413	6.41E-06	0.00018338
K03702	0.003032181	0.001660594	0.547656721	6.48E-06	0.00018338
K07258	0.000161788	0.000870397	5.379854966	6.54E-06	0.00018338
K09457	0.00011394	0.00072884	6.396702863	6.67E-06	0.00018338
K16785	0.000787793	0.002612522	3.316252918	6.79E-06	0.00018338
K03719	0.000154474	0.000760272	4.921676	6.82E-06	0.00018338
K06213	0.001138758	0.000436554	0.383359925	7.25E-06	0.000192333
K01752	0.002213446	0.000814075	0.36778621	7.35E-06	0.000192333

K09761	0.000414312	0.000996772	2.40584724	7.74E-06	0.000199962
K03430	0.001028114	0.00030269	0.294412604	7.99E-06	0.000203184
K02028	0.000508813	0.002202605	4.328911482	8.07E-06	0.000203184
K00761	0.00228226	0.00123334	0.540402699	8.38E-06	0.000208396
K09458	0.000805913	0.000230658	0.286207105	8.79E-06	0.000215736
K03615	0.000793219	0.000261701	0.329923546	8.89E-06	0.000215736
K01929	0.000365024	0.000827095	2.265862857	9.04E-06	0.000216752
K01995	0.00021442	0.000632695	2.950724101	9.45E-06	0.000222999
K01012	0.000536573	0.001149521	2.14234062	9.53E-06	0.000222999
K05837	0.000717844	0.00022812	0.317784667	9.64E-06	0.000222999
K00339	0.001412186	0.000451127	0.319453061	1.05E-05	0.000240485
K00426	0.000613604	0.00020464	0.333505238	1.06E-05	0.000240485
K01883	0.002543562	0.001427476	0.561211619	1.08E-05	0.00024117
K03589	8.32E-05	0.000545412	6.556988643	1.10E-05	0.00024117
K19271	0.001286928	0.000400442	0.311160829	1.11E-05	0.00024117
K01719	0.001020923	0.00032401	0.317369342	1.11E-05	0.00024117
K03561	5.60E-05	0.000403575	7.208415182	1.14E-05	0.000243914
K13993	0.00015201	0.000763916	5.025437982	1.20E-05	0.000253497
K00817	0.000220393	0.000871935	3.956270702	1.23E-05	0.000255569
K01818	0.001132982	0.000373377	0.329552567	1.23E-05	0.000255569
K02393	2.83E-05	0.000222406	7.85791495	1.25E-05	0.000256156
K02387	7.33E-05	0.000311644	4.252048941	1.28E-05	0.000256384
K00341	0.001104697	0.000321343	0.290887767	1.28E-05	0.000256384
K02551	0.000776912	0.000261566	0.336674445	1.29E-05	0.000256384
K10947	0.000475135	0.001242801	2.615677465	1.31E-05	0.000258768
K00625	0.000824385	0.000269348	0.326726129	1.34E-05	0.000258768

K00768	5.78E-05	0.000373272	6.453291774	1.34E-05	0.000258768
K02437	0.001003266	0.000382413	0.38116856	1.44E-05	0.00027509
K03799	0.000161244	0.000527269	3.270011046	1.46E-05	0.00027509
K00765	0.002010421	0.001108186	0.551220867	1.47E-05	0.00027509
K00954	0.000232509	0.000557279	2.396809853	1.48E-05	0.00027509
K03210	0.000647029	0.000320135	0.494777747	1.53E-05	0.000281163
K01734	0.000237591	0.000792445	3.335332146	1.59E-05	0.00028726
K02622	0.000855789	0.000334035	0.390324386	1.60E-05	0.00028726
K03565	0.000169376	0.000570574	3.368673096	1.60E-05	0.00028726
K00760	0.000180023	0.000520005	2.888554899	1.66E-05	0.000294859
K03470	0.000165527	0.000430447	2.600466074	1.74E-05	0.000306087
K01464	0.000109527	0.000597424	5.454588495	1.76E-05	0.000306989
K00721	0.000614934	0.000181885	0.295780192	1.78E-05	0.000307886
K01425	0.000608686	0.00018859	0.309831795	1.86E-05	0.000319658
K13953	2.21E-05	0.000219817	9.964339695	1.90E-05	0.000321372
K15553	1.54E-05	0.000147219	9.578922247	1.92E-05	0.000321372
K02510	1.08E-05	0.000156244	14.50067495	1.92E-05	0.000321372
K03705	0.00015322	0.000413791	2.70063453	1.95E-05	0.000323069
K02493	6.55E-05	0.000428713	6.546750974	2.02E-05	0.000331604
K00548	8.38E-05	0.000415008	4.951106391	2.21E-05	0.000359762
K02398	0.000116809	0.000457328	3.915193801	2.27E-05	0.000364949
K02913	0.011157797	0.005612119	0.502977368	2.27E-05	0.000364949
K19954	1.73E-05	0.000169132	9.772241085	2.31E-05	0.000367454
K02407	0.000235779	0.000630484	2.674047259	2.35E-05	0.000370508
K07447	0.001343474	0.000695851	0.517949121	2.39E-05	0.000374281
K02338	0.000785741	0.000361323	0.459849555	2.45E-05	0.000380428

K22024	1.95E-05	0.000170202	8.741378607	2.47E-05	0.000380637
K07056	5.06E-05	0.000355406	7.029021416	2.50E-05	0.000382136
K01938	0.000711882	0.000363979	0.511290977	2.59E-05	0.000391079
K09936	0.000256226	9.35E-05	0.364973795	2.59E-05	0.000391079
K03523	0.000244246	0.000778247	3.186330824	2.68E-05	0.000401292
K01956	0.000148727	0.000401108	2.696951174	2.71E-05	0.000401292
K03771	0.000456164	0.000103965	0.227910747	2.72E-05	0.000401292
K02041	0.000204455	0.000612352	2.995044039	2.81E-05	0.00041101
K00177	0.000884312	0.000230788	0.260980643	2.86E-05	0.000413723
K01749	5.52E-05	0.000384183	6.95941392	2.87E-05	0.000413723
K00042	9.98E-05	0.000523222	5.24301191	3.03E-05	0.000433256
K00249	4.01E-05	0.00023339	5.817806613	3.09E-05	0.000436303
K02769	0.000220253	0.000804661	3.653358166	3.12E-05	0.000436303
K22025	1.88E-05	0.000213175	11.35834235	3.16E-05	0.000436303
K05577	0.000124562	0.000590445	4.740181854	3.18E-05	0.000436303
K03150	0.000857032	0.00030255	0.353020363	3.20E-05	0.000436303
K02879	0.002722753	0.001534488	0.563579775	3.23E-05	0.000436303
K02963	0.005317551	0.003579337	0.673117663	3.23E-05	0.000436303
K07659	1.77E-05	0.000213641	12.07390875	3.24E-05	0.000436303
K01998	0.000264002	0.000698395	2.645417078	3.24E-05	0.000436303
K03320	0.000157556	0.000640117	4.062789138	3.35E-05	0.000448047
K00930	0.003143571	0.00170917	0.54370335	3.45E-05	0.000456389
K00337	0.001209864	0.000438487	0.362426341	3.46E-05	0.000456389
K01999	0.000236952	0.000953675	4.024765473	3.57E-05	0.000467319
K06158	0.000217115	0.000673208	3.100693291	3.65E-05	0.000474785
K02838	0.002311754	0.001345723	0.582122245	3.68E-05	0.000475791

K14188	7.08E-05	0	0	3.76E-05	0.000482499
K01034	3.39E-05	0.000226722	6.695980909	3.83E-05	0.000488096
K02470	0.000285358	0.000135412	0.474536044	3.87E-05	0.000489489
K02469	0.000169027	0.000413191	2.444528116	3.89E-05	0.000489489
K07302	9.52E-06	0.000161281	16.94392206	3.95E-05	0.000492927
K13930	0	0.000127929	inf	3.96E-05	0.000492927
K01515	0.00021037	0.000724809	3.445398666	3.99E-05	0.00049371
K07028	3.83E-05	0.000214998	5.606369459	4.09E-05	0.000500346
K02990	0.002988716	0.001691579	0.565988691	4.10E-05	0.000500346
K07726	3.13E-05	0.001239069	39.64724578	4.12E-05	0.000500346
K01951	0.005121816	0.003038183	0.593184839	4.19E-05	0.000505506
K01491	0.003257933	0.001929555	0.592263452	4.28E-05	0.000513349
K03564	0.000515308	0.000199888	0.387899822	4.40E-05	0.000523814
K19784	5.99E-05	0.000448118	7.476491445	4.47E-05	0.000525071
K03149	0.000190801	0.000762626	3.996973099	4.49E-05	0.000525071
K14338	1.83E-05	0.0001714	9.385197778	4.51E-05	0.000525071
K02048	3.34E-05	0.000215286	6.454439809	4.51E-05	0.000525071
K08363	0.000133274	0.000737739	5.535495292	4.70E-05	0.000543843
K01687	0.003994663	0.002574865	0.644576181	4.77E-05	0.000548301
K00632	2.46E-05	0.000144943	5.885278919	4.84E-05	0.000553358
K01129	0.000173464	0.000413235	2.382243614	4.91E-05	0.000556182
K01297	8.91E-05	0.000482553	5.418488894	4.92E-05	0.000556182
K01270	0.000694201	0.000209128	0.301249388	4.98E-05	0.000557766
K16203	8.91E-06	0.000130891	14.68657017	4.99E-05	0.000557766
K03594	0.000523321	0.000154457	0.295148088	5.02E-05	0.000557766
K03666	1.07E-05	0.000161611	15.11329127	5.08E-05	0.000561721

K15268	1.50E-05	0.000167634	11.16600199	5.15E-05	0.00056377
K02575	2.03E-05	0.000129243	6.367721071	5.16E-05	0.00056377
K01567	0.000154594	0.000413944	2.677621783	5.28E-05	0.000571646
K06989	0.000202448	0.000564323	2.787489523	5.30E-05	0.000571646
K01770	0.001208596	0.00065299	0.54028816	5.31E-05	0.000571646
K03500	7.58E-05	0.000379491	5.009451921	5.38E-05	0.000575273
K11529	2.92E-05	0.000178208	6.10802868	5.48E-05	0.0005836
K18220	0.005169184	0.003082427	0.596308204	5.53E-05	0.000585603
K03981	1.85E-05	0.00014067	7.589416627	5.56E-05	0.000585875
K13789	0.000341335	0.00086706	2.540204581	5.76E-05	0.000602392
K03654	7.55E-05	0.00034786	4.605808091	5.78E-05	0.000602392
K00626	0.000104395	0.000373959	3.582150636	5.93E-05	0.000614529
K02227	0.000174427	0.000482932	2.768686007	5.98E-05	0.000615728
K06162	2.32E-05	0.000136608	5.89507193	6.00E-05	0.000615728
K01295	2.80E-05	0.000155772	5.56775686	6.03E-05	0.000615728
K03091	0.000487201	0.001018668	2.090855611	6.10E-05	0.000616025
K02114	0.000554984	0.001369574	2.467773152	6.10E-05	0.000616025
K00946	0.001114325	0.00053588	0.480901278	6.17E-05	0.00062061
K02057	0.000670968	0.001656019	2.468105229	6.26E-05	0.000626263
K00645	0.001318433	0.000279115	0.211701768	6.38E-05	0.000634876
K12960	0.00018856	0.000567699	3.010705383	6.42E-05	0.0006352
K09939	2.71E-05	0.000191634	7.0643784	6.48E-05	0.000638047
K01262	0.000576524	0.000239002	0.414556564	6.53E-05	0.00063893
K03667	0.00010744	0.000481135	4.478188819	6.55E-05	0.00063893
K11069	0.000261016	0.000627017	2.402219114	6.60E-05	0.00064084
K02017	0.000128541	0.000526816	4.098430085	6.77E-05	0.000652343

K13542	0.000320122	8.49E-05	0.265141009	6.81E-05	0.000652343
K00241	0.00112584	0.000428529	0.38063072	6.85E-05	0.000652343
K08224	2.66E-05	0.000171092	6.44123486	6.85E-05	0.000652343
K00286	8.87E-05	0.000467112	5.264180467	6.88E-05	0.000652343
K06217	0.002606152	0.001072942	0.411695862	7.00E-05	0.00066015
K01879	0.000102695	0.000462761	4.506164632	7.04E-05	0.000661228
K09822	0.000130217	0.000595616	4.574023743	7.08E-05	0.000661913
K07396	1.07E-05	0.000123015	11.45798509	7.30E-05	0.000678884
K02400	4.31E-05	0.000227559	5.281046459	7.37E-05	0.000681803
K00013	0.001197622	0.000569477	0.475506673	7.45E-05	0.000686608
K01438	0.000622171	0.000222612	0.357798675	7.74E-05	0.0007102
K06134	2.61E-05	0.000173764	6.65821666	7.86E-05	0.000717687
K06145	2.93E-05	0.000200015	6.819857407	7.94E-05	0.000718598
K02010	3.75E-05	0.000241755	6.440097606	7.94E-05	0.000718598
K03464	1.96E-05	0.000196433	9.999322682	8.11E-05	0.000723751
K00135	2.05E-05	0.000133347	6.520608611	8.11E-05	0.000723751
K03270	3.32E-05	0.000196204	5.916176683	8.11E-05	0.000723751
K07001	6.92E-05	0.00038794	5.606635204	8.37E-05	0.000743163
K00029	0.000269983	6.88E-05	0.254910895	8.71E-05	0.000769951
K02008	0	0.000284534	inf	8.75E-05	0.000769951
K07397	3.10E-05	0.000225826	7.276428836	8.82E-05	0.000769951
K07004	1.01E-05	0.000103381	10.21581408	8.82E-05	0.000769951
K07491	0.000170919	0.000645689	3.77774315	9.18E-05	0.000794888
K07588	0.001144925	0.000440241	0.384515159	9.19E-05	0.000794888
K09011	0.000541962	0.00014351	0.264797477	9.27E-05	0.000797187
K02967	0.002881721	0.001887745	0.65507551	9.33E-05	0.000797187

K13292	0.000267737	0.000556562	2.078760447	9.33E-05	0.000797187
K01419	0.000120095	0.000490974	4.08820078	9.41E-05	0.000800297
K09788	4.04E-05	0.000236404	5.846011858	9.59E-05	0.000811868
K11754	0.000244128	0.000776553	3.180921028	9.69E-05	0.00081691
K10563	0.00010939	0.000455502	4.164003597	9.80E-05	0.00081985
K06001	2.08E-05	0.000348345	16.71858728	9.82E-05	0.00081985
K02347	4.13E-05	0.000228265	5.521112865	9.85E-05	0.00081985
K02874	0.004930023	0.003365627	0.682679753	9.92E-05	0.000822813
K07114	0.000976968	0.000504541	0.516435832	0.000101521	0.000838282
K02480	2.49E-05	0.000170316	6.839178588	0.000103163	0.000845486
K18682	0.002035031	0.001228044	0.603452376	0.000103277	0.000845486
K00457	4.24E-05	0.000180318	4.255142911	0.000103668	0.000845486
K03717	0.000140535	0.000575354	4.094035695	0.000105839	0.000859668
K17324	2.92E-05	0.000155715	5.327270742	0.000108533	0.000877969
K01056	0.000861657	0.000485825	0.563826163	0.000109881	0.000882119
K02906	0.004429433	0.003119444	0.704253589	0.000109932	0.000882119
K22468	4.06E-05	0.000199231	4.906648451	0.000110441	0.000882639
K05810	0.000294477	0.000565749	1.921196749	0.000112424	0.000893702
K01479	1.45E-05	8.76E-05	6.041863563	0.000112723	0.000893702
K03458	4.89E-05	0.000345654	7.072613529	0.000114094	0.000900983
K10907	0.000290889	0.001049099	3.606531629	0.000115587	0.000909162
K00287	0.00021777	0.000592752	2.721915276	0.000116366	0.000911685
K03773	0.000401785	0.000154832	0.385361015	0.000119425	0.000931981
K02986	0.002921899	0.001847826	0.632405972	0.000121713	0.000946127
K07666	3.19E-05	0.000189612	5.946511649	0.000123782	0.000954154
K02420	8.68E-05	0.000302025	3.480907564	0.00012432	0.000954154

K00176	0.001014152	0.000367539	0.362410388	0.000125052	0.000954154
K13043	2.25E-05	0.00031972	14.23586379	0.000126046	0.000954154
K13774	2.22E-05	0.000169543	7.630462848	0.000126102	0.000954154
K03412	2.61E-05	0.000164214	6.296770745	0.000126102	0.000954154
K05539	1.86E-05	0.000141053	7.577689855	0.000126102	0.000954154
K01912	0.001346386	0.000627819	0.46629931	0.000130355	0.000980105
K07642	4.53E-05	0.00022107	4.885360033	0.000131009	0.000980105
K01574	4.64E-05	0.000238157	5.130131904	0.000131009	0.000980105
K08738	1.51E-05	0.000202471	13.37681096	0.00013235	0.000986431
K00278	0.000110191	0.000460823	4.182049284	0.000133105	0.000986585
K00097	0.000540933	0.000203689	0.376551267	0.000134143	0.000986585
K13873	1.52E-05	9.97E-05	6.538104641	0.000134446	0.000986585
K11717	0.000558583	0.000212613	0.380629445	0.000134629	0.000986585
K07234	2.56E-05	0.000169357	6.622843941	0.00013485	0.000986585
K04041	0.000151952	0.000393469	2.58942581	0.000137125	0.000999553
K03182	2.94E-05	0.000153288	5.213173351	0.000138606	0.001006666
K02110	0.000560494	0.001473341	2.628647232	0.000140473	0.001016511
K03168	0.000162606	0.000518011	3.185692774	0.000141408	0.001017529
K01142	0.000140575	0.000618914	4.402735779	0.000142184	0.001017529
K09461	4.97E-05	0.000240196	4.833587693	0.000142334	0.001017529
K10541	0.000110636	0.000298583	2.698783322	0.000142659	0.001017529
K01448	0.000115136	0.000601313	5.222634767	0.000145754	0.001031789
K11065	2.22E-05	0.00016718	7.51714559	0.000146332	0.001031789
K01255	1.11E-05	0.000112121	10.13333814	0.000146666	0.001031789
K01993	1.79E-05	0.000161154	8.983411407	0.000146732	0.001031789
K00285	7.50E-05	0.000320376	4.270504431	0.000149972	0.00105086

K19340	3.00E-05	0.000186092	6.205969209	0.000153918	0.001067898
K00651	0.002943666	0.002038739	0.692585218	0.000155016	0.001067898
K04043	0.003652286	0.002273918	0.622601275	0.000155023	0.001067898
K03273	2.49E-05	0.000162857	6.552473695	0.000156622	0.001067898
K02563	2.65E-05	0.000187345	7.076682158	0.000156622	0.001067898
K13932	4.34E-05	0.000250333	5.770809459	0.000156776	0.001067898
K01595	2.75E-05	0.000137403	4.998275405	0.000156776	0.001067898
K18369	4.28E-05	0.000267633	6.246105481	0.000156776	0.001067898
K00991	0.000270845	0.000471705	1.741606558	0.000157647	0.001067898
K01736	0.002720005	0.001727057	0.634946114	0.000158156	0.001067898
K00059	0.000495451	0.000197803	0.399238687	0.000158306	0.001067898
K00791	8.69E-05	0.000344173	3.962439311	0.000159344	0.001071267
K10763	2.93E-05	0.000189422	6.468519963	0.000162625	0.001089645
K03569	0.00017729	0.000628891	3.547254448	0.000164039	0.001092078
K03411	4.60E-05	0.000226131	4.912852056	0.000164086	0.001092078
K00759	0.001766285	0.000854361	0.483704893	0.000166196	0.001102433
K01733	0.00017572	0.000417163	2.374021616	0.000167126	0.001104919
K02081	3.98E-05	1.83E-06	0.04603793	0.000175346	0.001149469
K01465	0.000226829	0.000531669	2.343918836	0.000175551	0.001149469
K00179	3.14E-05	0.000404498	12.89762757	0.000175597	0.001149469
K19745	2.76E-05	0.000156946	5.677568742	0.000180695	0.001176479
K21010	2.31E-05	0.00013099	5.674908049	0.000180906	0.001176479
K11755	3.52E-05	0.000381861	10.84525857	0.000182002	0.001179754
K03518	0.000117098	0.000517745	4.421466346	0.000187232	0.001202033
K04562	3.54E-05	0.00020379	5.750653917	0.000187335	0.001202033
K03924	0.000372454	0.00017285	0.464083299	0.000187829	0.001202033

K11209	5.28E-05	0.0002621	4.965727526	0.000187855	0.001202033
K02233	0.000242389	0.000484056	1.997024065	0.000188762	0.001203963
K00979	7.77E-06	9.92E-05	12.76679602	0.000192264	0.001215922
K03625	0.000903815	0.000536625	0.593733816	0.00019312	0.001215922
K06075	0	0.000281942	inf	0.000193702	0.001215922
K07149	0	9.72E-05	inf	0.000193702	0.001215922
K15726	4.82E-05	0.000182081	3.776831876	0.000194303	0.001215922
K01531	5.28E-05	0.000203947	3.863087947	0.000194303	0.001215922
K06394	0.000185745	0.000452237	2.434716723	0.000195216	0.001217808
K03535	0.000146332	0.000630912	4.311512446	0.000195886	0.001218163
K02036	0.000310614	0.000639315	2.05822871	0.000197021	0.001221409
K03406	0.000437366	0.001105177	2.526889025	0.000198226	0.001224086
K03710	0.000106567	0.00038928	3.652922207	0.000198824	0.001224086
K11103	8.10E-05	0.000294293	3.635360839	0.000199383	0.001224086
K03776	3.72E-05	0.000211998	5.69566013	0.000199914	0.001224086
K00681	2.42E-05	0.000312228	12.89750876	0.000201195	0.001225141
K00975	0.000430542	0.00095003	2.20658888	0.000201317	0.001225141
K12262	0.000122762	0.000508186	4.139603044	0.000204534	0.001235654
K06919	0.000339851	0.000842167	2.478045701	0.000204576	0.001235654
K07645	3.82E-05	0.000158659	4.158309891	0.000204907	0.001235654
K00133	0.001320728	0.000792803	0.600277705	0.000208758	0.001251317
K02968	0.003648376	0.002576335	0.70615931	0.000208762	0.001251317
K06317	0.000199336	0.000701161	3.51748819	0.000210898	0.001260323
K03554	4.09E-05	0.000190205	4.651994081	0.000211654	0.001261052
K02072	2.97E-05	0.000162169	5.458664963	0.00021323	0.001266647
K02567	3.38E-05	0.000150611	4.452654362	0.00021651	0.001281389

K16787	0.000447357	0.001170165	2.615727804	0.000216999	0.001281389
K06142	6.22E-05	0.000284695	4.576408399	0.000221863	0.001302837
K00248	6.80E-05	0.000188169	2.766454376	0.000221941	0.001302837
K03563	0.000201918	0.000484996	2.401950373	0.000223502	0.001308141
K01251	4.65E-05	0.00024106	5.182839836	0.000224658	0.001311057
K13875	2.59E-05	0.000136707	5.27051232	0.000227431	0.001323356
K00455	1.90E-05	0.00014132	7.428744721	0.000228195	0.001323929
K02770	0.000282193	0.000807392	2.861139457	0.000229285	0.001326386
K03772	1.80E-05	0.000158646	8.797718648	0.000235362	0.001357598
K04091	9.84E-05	0.000363555	3.694896966	0.000238347	0.001366969
K07649	5.04E-05	0.000252447	5.008656981	0.000238361	0.001366969
K03040	0.00210728	0.001494032	0.708986045	0.000239162	0.001367622
K03644	0.000725668	0.000349945	0.482237532	0.000242143	0.001380699
K03340	1.98E-05	0.000281946	14.26067137	0.000243623	0.001384314
K01845	3.60E-05	0.000203784	5.668139495	0.000244385	0.001384314
K00845	0.001272695	0.000428567	0.336739359	0.000244872	0.001384314
K09975	1.38E-05	0.000140035	10.12275625	0.000245559	0.001384314
K13928	6.29E-05	0.000232373	3.691650042	0.000250738	0.001409516
K21420	2.70E-05	0.000146591	5.435366166	0.000257043	0.00143573
K07105	0.000112495	0.000300255	2.669053478	0.000257505	0.00143573
K02217	0.001495953	0.000259309	0.173340326	0.000257566	0.00143573
K02863	0.002738097	0.001844496	0.673641734	0.000258809	0.001438631
K13483	8.70E-06	9.68E-05	11.12396462	0.000259658	0.001439332
K09903	0.001737196	0.001005657	0.578896915	0.000263854	0.001454431
K02160	2.79E-05	0.000155128	5.559988138	0.000264487	0.001454431
K00937	0.000256023	0.000501462	1.958659821	0.000264575	0.001454431

K05275	5.82E-05	0.0002213	3.805040984	0.000269377	0.001472694
K00281	3.74E-05	0.000161701	4.32875654	0.000269377	0.001472694
K00558	0.000484062	0.001711506	3.535712304	0.000274399	0.00149604
K03809	2.86E-05	0.000148821	5.211556325	0.000275546	0.001498185
K01118	8.93E-05	0.000416991	4.670226941	0.000284368	0.001537808
K11068	3.82E-05	0.000172986	4.532250677	0.000284379	0.001537808
K02026	0.000360586	0.000895385	2.48313782	0.000286608	0.001545665
K01007	6.06E-05	0.000224591	3.703789163	0.000291632	0.001562875
K15727	5.45E-05	0.000190458	3.495628004	0.000291632	0.001562875
K01805	2.20E-05	0.000238893	10.86493201	0.000293404	0.001562875
K03630	0.000277513	0.000643797	2.31988004	0.000294233	0.001562875
K00574	2.62E-05	0.000143112	5.456004639	0.000295132	0.001562875
K01599	2.61E-05	0.000139358	5.349013493	0.000295297	0.001562875
K08994	3.48E-05	0.000187917	5.392830589	0.000295297	0.001562875
K02113	0.000431253	0.000803105	1.86225973	0.000297422	0.001569944
K02283	4.80E-05	0.000175853	3.663550023	0.000298866	0.001573397
K07574	0.000223797	0.000682694	3.050508034	0.000300339	0.001576976
K06136	5.67E-05	0.00024124	4.251485144	0.000301403	0.001578401
K03521	6.58E-05	0.000184147	2.799315207	0.000311125	0.001622563
K05816	1.45E-05	0.000103051	7.103759535	0.000311467	0.001622563
K11071	0.000192796	0.00056926	2.952654823	0.000312373	0.001623035
K04516	0.000977485	0.000304094	0.311098459	0.000314381	0.001625343
K07088	0.000698019	0.001823323	2.612140428	0.000314826	0.001625343
K01706	2.34E-05	0.000118356	5.066069039	0.000316098	0.001625343
K01061	8.00E-05	0.000296165	3.703743176	0.000316725	0.001625343
K05595	0.000937316	0.000242458	0.258673073	0.000317165	0.001625343

K07130	6.02E-06	8.14E-05	13.53132023	0.000317718	0.001625343
K00928	0.000406372	0.000108714	0.26752323	0.000318989	0.001627661
K10001	0.00011222	0.000429152	3.824211713	0.00032228	0.001635931
K03670	0.000105793	0.000392845	3.713350751	0.00032228	0.001635931
K06041	0.001934468	0.000493033	0.254867497	0.000323076	0.001635931
K12527	2.95E-05	0.00031478	10.68558376	0.000324592	0.001639438
K00748	1.72E-05	0.000105144	6.098730161	0.000328741	0.001656189
K03694	3.42E-05	0.000171698	5.023084116	0.000333154	0.001662475
K00128	2.60E-05	0.00014295	5.506011219	0.000333916	0.001662475
K19166	2.85E-05	0.000152024	5.339992075	0.000333916	0.001662475
K00766	0.001077838	0.000553876	0.513877069	0.000334016	0.001662475
K08678	5.66E-05	0.000246137	4.351696551	0.000334166	0.001662475
K01926	0.000810059	0.000462966	0.571521118	0.000337655	0.001675643
K01684	3.66E-05	0.000150303	4.109349038	0.000338834	0.001677311
K10670	0.000105331	1.10E-05	0.104833683	0.000340436	0.001681063
K02313	0.001406773	0.000910608	0.647302522	0.000345317	0.001700943
K00891	0.000303273	0.00060878	2.007369202	0.000346293	0.001701538
K02609	4.28E-05	0.000212654	4.974346592	0.00035471	0.00173286
K02278	7.62E-05	0.000266592	3.49729256	0.000355923	0.00173286
K09014	3.94E-05	0.000404297	10.260861	0.000356786	0.00173286
K02203	0.000123979	2.74E-05	0.22094556	0.000356819	0.00173286
K01682	4.26E-05	0.00019446	4.562170341	0.000358592	0.00173286
K03576	4.54E-05	0.000197954	4.360358503	0.000358592	0.00173286
K07156	3.17E-05	0.000195225	6.154067834	0.000359679	0.00173286
K07345	3.96E-05	0.00018194	4.594230952	0.000359914	0.00173286
K01720	2.38E-05	0.000162494	6.816757335	0.000360505	0.00173286

K14287	3.03E-05	0.000129003	4.26373228	0.000362789	0.001739638
K01433	2.91E-05	0.000318182	10.93763318	0.00036449	0.001743593
K01869	0.002751323	0.001817576	0.66061906	0.000365617	0.00174479
K00989	1.96E-05	0.00012042	6.148693685	0.000369415	0.001758701
K03577	3.01E-05	0.000140611	4.671602982	0.000373018	0.001771611
K00852	2.43E-05	0.000136927	5.64600759	0.000387671	0.001833475
K09779	0.000108682	0.000338268	3.112465674	0.000387886	0.001833475
K07267	4.98E-05	0.000207475	4.166677527	0.000392033	0.001848688
K05833	0.000151986	0.000339333	2.232652485	0.000394711	0.001856914
K04096	2.11E-05	0.000129119	6.130694626	0.000399609	0.001868513
K02052	2.00E-05	0.000167135	8.336774222	0.000399852	0.001868513
K21053	3.65E-05	0.000158553	4.349562303	0.000401702	0.001868513
K07091	3.99E-05	0.000167003	4.181366829	0.000401792	0.001868513
K02834	0.001576327	0.000953464	0.604864348	0.000401871	0.001868513
K19577	1.58E-05	0.000127179	8.058024492	0.000404627	0.001873309
K13643	2.19E-05	0.000124478	5.687080659	0.000404785	0.001873309
K01887	0.003144507	0.002226949	0.70820282	0.000409563	0.001889387
K03782	1.68E-05	0.000113607	6.751327836	0.000410158	0.001889387
K00496	6.24E-05	0.000235039	3.766661708	0.000413317	0.001895165
K10545	5.51E-05	0.000215524	3.911675917	0.000413317	0.001895165
K02757	0.00012716	0.000294754	2.317987729	0.000417501	0.001901206
K02755	0.00012716	0.000294754	2.317987729	0.000417501	0.001901206
K02756	0.00012716	0.000294754	2.317987729	0.000417501	0.001901206
K00652	0.000479791	0.000210548	0.438833274	0.000419344	0.001905238
K19883	0.000166918	0	0	0.00042436	0.001920215
K01627	0.000126503	0.000475547	3.759163869	0.000424599	0.001920215

K06941	0.000245761	0.000510688	2.077987483	0.000426379	0.001920215
K01113	2.14E-05	0.000119366	5.578683224	0.00042737	0.001920215
K01428	3.31E-05	0.000150901	4.56322241	0.000427465	0.001920215
K06938	3.66E-05	0.000182253	4.980447233	0.000435677	0.001952695
K09990	2.63E-05	0.000138426	5.256295233	0.000439409	0.001964996
K03300	3.01E-05	0.000170476	5.668284438	0.000449719	0.002006513
K01477	4.46E-05	0.000181012	4.058932948	0.000450709	0.002006513
K04075	0.000250598	0.000669241	2.67057435	0.000467771	0.00207782
K01963	0.000696522	0.000231281	0.332050771	0.000475224	0.002099187
K03117	3.34E-05	0.0001719	5.139699996	0.000475349	0.002099187
K00833	2.96E-05	0.000128826	4.355460968	0.000475745	0.002099187
K03816	0.00093309	0.000478868	0.51320684	0.000481113	0.002116971
K01920	3.65E-05	0.000186338	5.106303054	0.000483362	0.002116971
K21277	3.33E-05	0.000152268	4.568895789	0.000483879	0.002116971
K03885	2.88E-05	0.000140453	4.869955152	0.000484031	0.002116971
K03313	2.18E-05	0.000368136	16.91523092	0.000490735	0.00214003
K03474	0.001006603	0.000477883	0.474748444	0.000491454	0.00214003
K07636	4.53E-06	0.000210653	46.49228721	0.00049626	0.002156239
K00344	3.73E-05	0.000138899	3.727758139	0.00050088	0.002168985
K01914	0.001893104	0.00109349	0.577617825	0.000502739	0.002168985
K19955	0.000103603	1.57E-06	0.015184885	0.000503189	0.002168985
K07462	0.000232039	0.000632391	2.725371735	0.000504202	0.002168985
K00368	5.25E-05	0.000202586	3.857596871	0.000504643	0.002168985
K01139	3.01E-05	0.000152066	5.047567396	0.000508057	0.002174265
K07735	2.66E-05	0.000137313	5.168044454	0.000508057	0.002174265
K01707	3.35E-05	0.000170733	5.103798972	0.000513969	0.002188399

K02109	0.000281012	0.000596899	2.124102705	0.000516627	0.002188399
K07138	0.000256228	0.00061445	2.398059678	0.000518668	0.002188399
K01187	0.000125607	0.000562823	4.480810037	0.000518952	0.002188399
K03439	0.000129709	0.000533597	4.113789983	0.00052017	0.002188399
K00275	2.62E-05	0.000166683	6.358157823	0.000520611	0.002188399
K01429	1.27E-05	0.000176884	13.96478053	0.000520626	0.002188399
K04062	2.34E-05	0.000291335	12.43328546	0.000520626	0.002188399
K17227	5.06E-05	0.000205364	4.05623787	0.000522278	0.002188399
K01573	0.000558888	0.000214635	0.384038965	0.000522356	0.002188399
K01026	0.000119799	2.46E-05	0.205043116	0.000531326	0.002214739
K06153	0.001868046	0.001266518	0.677990656	0.000531982	0.002214739
K03088	0.008935134	0.013505392	1.511492918	0.000531982	0.002214739
K01265	0.000376458	0.000217759	0.578442458	0.000536961	0.002229171
K01991	3.98E-05	0.000278613	7.003549653	0.000537689	0.002229171
K01613	0.000767919	0.000404468	0.526706313	0.000540667	0.002236856
K07336	3.83E-05	0.000169251	4.419287849	0.000542994	0.002237754
K18778	3.30E-05	0.000160307	4.853962351	0.000543133	0.002237754
K05916	4.61E-05	0.000174924	3.796236924	0.000546256	0.002245969
K00648	0.001848169	0.001009817	0.546388026	0.000549613	0.00225087
K02224	0.00025507	0.000556561	2.181990678	0.00054971	0.00225087
K01897	0.000246779	0.000682386	2.765174248	0.000559035	0.002269591
K19342	3.32E-05	0.000175683	5.295091401	0.000559928	0.002269591
K11070	0.000603131	0.000295051	0.489199601	0.000559966	0.002269591
K01893	0.001723161	0.001051655	0.6103057	0.000561821	0.002269591
K01487	0.000115856	0.000446164	3.851021125	0.000563624	0.002269591
K07347	3.14E-05	0.000126487	4.022905653	0.000564444	0.002269591

K02014	9.34E-05	0.000369322	3.953516696	0.000564547	0.002269591
K15977	6.92E-05	0.00027118	3.916047711	0.000564547	0.002269591
K00824	4.79E-05	0.000226619	4.728872982	0.000564547	0.002269591
K02121	0.000777417	0.000411565	0.529400062	0.000574923	0.002306647
K00261	3.14E-05	0.000151163	4.816941823	0.0005803	0.002318895
K09694	2.62E-05	0.000130021	4.95651521	0.000580306	0.002318895
K03596	0.00285466	0.002146019	0.751759931	0.000583416	0.0023246
K00860	0.000476136	0.000232742	0.488814026	0.00058407	0.0023246
K07082	0.000136891	0.000481743	3.519169932	0.00059338	0.00235557
K00616	0.001491736	0.000826763	0.554228632	0.000594219	0.00235557
K00453	3.17E-05	0.00014227	4.483180411	0.000596922	0.00236158
K02045	0.000107601	0.000379879	3.530429943	0.000612131	0.002416945
K09695	3.33E-05	0.00015117	4.54076536	0.000619681	0.002437087
K02298	5.22E-05	0.000235188	4.504355311	0.000619681	0.002437087
K02066	0.000523197	0.000217382	0.415489163	0.000624488	0.002451147
K00451	5.73E-05	0.000208217	3.636852579	0.000626752	0.002455188
K01679	0.000166729	0.000504022	3.023006708	0.000629066	0.002459412
K01961	4.18E-05	0.000166841	3.991207788	0.000631019	0.002462211
K02528	0.000498275	0.00078773	1.580914954	0.000645122	0.002512314
K01759	0.000199264	0.000582507	2.923287318	0.00065044	0.00252342
K02619	3.92E-05	0.000213994	5.459042809	0.00065051	0.00252342
K00575	6.34E-05	0.000201556	3.181252133	0.0006544	0.002533572
K01486	3.81E-05	0.000278236	7.299184546	0.000658388	0.002538522
K19405	3.17E-05	0.000256343	8.077489519	0.000658388	0.002538522
K01894	1.04E-05	0.000125668	12.0818671	0.000659505	0.002538522
K21405	3.34E-05	0.000146737	4.389351141	0.000661697	0.002542041

K00895	0.001588171	0.000419854	0.264363318	0.000669441	0.002561167
K03709	1.59E-05	0	0	0.000670702	0.002561167
K09940	1.40E-05	0	0	0.000670702	0.002561167
K00331	0.002797683	0.001560636	0.557831487	0.000671824	0.002561167
K09013	0.000855347	0.000388078	0.453707587	0.00067385	0.00256398
K02460	1.53E-05	0.00010762	7.043633577	0.000692465	0.00262978
K01259	7.34E-05	0.000305357	4.160203802	0.00069524	0.00263529
K00573	2.05E-05	0.000144177	7.048511251	0.000700561	0.002644408
K01011	1.55E-05	0.000101888	6.552689373	0.000700561	0.002644408
K03616	0.000174683	0.000421316	2.411882704	0.000701632	0.002644408
K01737	0.000800689	0.000399025	0.498351946	0.000705012	0.002647543
K00210	0.001154653	0.000448288	0.388244301	0.000705124	0.002647543
K00380	3.75E-05	0.000164459	4.38532476	0.000706523	0.002647799
K07152	2.88E-05	0.000158574	5.515466945	0.000710704	0.00265519
K03073	0.000254955	0.000945031	3.706654293	0.000711164	0.00265519
K22318	4.01E-05	0.000178516	4.455253574	0.000737981	0.002750154
K00356	2.17E-05	0.000128231	5.899676395	0.000746513	0.002767221
K00329	2.17E-05	0.000128231	5.899676395	0.000746513	0.002767221
K04756	3.52E-05	0.000167104	4.740910236	0.000746733	0.002767221
K02379	3.68E-05	0.000295724	8.027388426	0.00075917	0.002808081
K00812	0.000350672	0.000103802	0.296008934	0.00077673	0.002867703
K03438	0.000728316	0.001142357	1.568491803	0.000782959	0.002880725
K07506	5.41E-05	0.000221076	4.089384887	0.000783152	0.002880725
K03787	0.000112187	0.000502821	4.481987584	0.000786044	0.002886028
K04044	3.20E-05	0.000138186	4.32217212	0.00080144	0.002926303
K00956	7.40E-05	0.000283386	3.83155195	0.000803006	0.002926303

K11159	3.56E-05	0.000158466	4.445358059	0.000804171	0.002926303
K17713	3.13E-05	0.00014368	4.583176937	0.000804171	0.002926303
K02199	3.34E-05	0.000147317	4.415302493	0.000804366	0.002926303
K02768	0.000201714	0.000714369	3.541499911	0.000810063	0.00294165
K04517	7.02E-05	0.000348903	4.96813152	0.000813332	0.002948144
K03803	3.24E-05	0.000253417	7.824990905	0.000815523	0.002950712
K18120	4.62E-05	0.00034595	7.4822255	0.000824902	0.002979227
K00878	4.20E-06	0.000202652	48.28232054	0.000831709	0.002998372
K01468	1.67E-05	0.000134536	8.033406856	0.000834283	0.003002212
K01008	0.000929813	0.000373143	0.40131029	0.000842933	0.003027863
K02118	0.001821032	0.001196461	0.657023459	0.000857534	0.003074762
K07552	1.61E-05	0.000111024	6.901294399	0.000867329	0.003104288
K02071	0.000155514	0.00045237	2.908870986	0.000873885	0.003120895
K19622	4.88E-05	0.000165454	3.39280976	0.000876673	0.003120895
K07246	5.38E-05	0.000180184	3.349312692	0.000876673	0.003120895
K07238	4.36E-05	0.000317434	7.284497009	0.000882402	0.003124521
K02598	4.39E-05	0.000271286	6.176190341	0.000882402	0.003124521
K00033	5.52E-05	0.000292301	5.2966568	0.000882402	0.003124521
K12524	3.65E-05	0.000310766	8.514507954	0.000885342	0.003129362
K00763	0.000187296	0.000521396	2.783802219	0.000889379	0.003138057
K08998	0.005014364	0.002526013	0.503755347	0.000901151	0.003173967
K02674	3.27E-05	0.000120881	3.698491159	0.000914659	0.003215851
K03752	0.000203488	0.00046074	2.264209172	0.000920738	0.003231515
K03664	0.002207484	0.00140075	0.634546153	0.000933893	0.003261997
K02672	3.10E-05	0.00011178	3.611016968	0.000934341	0.003261997
K22186	3.46E-05	0.00015033	4.346958037	0.000934341	0.003261997

K16923	2.61E-05	0	0	0.000945135	0.003293902
K07037	0.000404895	0.000184649	0.456042334	0.000948221	0.00329888
K03768	0.000187224	0.000465523	2.486450762	0.000953798	0.003312491
K03978	4.76E-05	0.000182889	3.845311511	0.000958472	0.003322925
K07079	0	0.000178338	inf	0.000964623	0.003325933
K21613	0	0.000146673	inf	0.000964623	0.003325933
K06889	0	4.54E-05	inf	0.000964623	0.003325933
K00943	2.87E-05	0.000183314	6.389549817	0.000966025	0.003325933
K02275	4.47E-05	0.000152525	3.414851373	0.000976547	0.003356354
K01079	0.000434284	0.000233079	0.536697708	0.000984175	0.003376739
K00854	0.000335201	0.000647123	1.93055274	0.000994923	0.00340774
K18672	0.000129071	0.000318725	2.469381516	0.001000536	0.003421077
K00821	0.001699758	0.000818204	0.481364958	0.001009991	0.003446987
K08715	1.13E-05	9.53E-05	8.406391177	0.001011578	0.003446987
K01756	7.68E-05	4.98E-05	0.648710588	0.001014803	0.003452065
K03778	0.000887552	0.000512791	0.577758607	0.001017973	0.003456939
K01588	0.000120349	6.79E-05	0.564420676	0.001024551	0.003473349
K07323	2.58E-05	0.000128157	4.967202604	0.001038588	0.003509797
K06138	6.31E-05	0.000223805	3.54437003	0.001038829	0.003509797
K01534	0.000110407	0.000271443	2.458558342	0.001046581	0.003511689
K00982	2.05E-05	9.51E-05	4.644067301	0.001047857	0.003511689
K06189	3.21E-05	0.000139895	4.357189728	0.001047857	0.003511689
K15738	2.38E-05	0.000103713	4.363869601	0.001047857	0.003511689
K03342	3.09E-05	0.000134155	4.336584805	0.001048213	0.003511689
K01069	0.000185082	0.000497052	2.68557099	0.001069888	0.003578281
K07277	0.000904345	0.000299004	0.330630631	0.001071921	0.003579065

K01881	0.00386699	0.002849076	0.736768417	0.001075992	0.003586641
K01585	0.001836335	0.00122983	0.669719835	0.001104327	0.003674933
K01066	0.000114076	0.000285458	2.502345132	0.001116935	0.003710687
K03841	2.51E-05	0.000104378	4.159696342	0.001131435	0.003746501
K04078	0.002680785	0.001833479	0.683933614	0.001134196	0.003746501
K19621	3.17E-05	0.000130088	4.104340553	0.001136816	0.003746501
K15552	2.86E-05	0.000136151	4.76529252	0.001137129	0.003746501
K06204	5.48E-05	0.000211228	3.853976582	0.001137129	0.003746501
K21575	3.29E-05	0.000298043	9.070733913	0.001154356	0.003796974
K04066	3.28E-05	0.000221021	6.737314102	0.001171068	0.003818517
K06215	7.25E-05	7.68E-05	1.058904918	0.001171767	0.003818517
K00375	1.66E-05	0.000100853	6.067947506	0.001173965	0.003818517
K19416	2.00E-05	0.00010543	5.278070119	0.001173965	0.003818517
K07278	2.07E-05	0.000123289	5.942089734	0.001174448	0.003818517
K01423	1.94E-05	9.96E-05	5.139920739	0.001177236	0.003818517
K04751	3.87E-05	0.000228029	5.898249141	0.001177236	0.003818517
K09165	8.91E-05	0.000306517	3.441881673	0.001178176	0.003818517
K21012	3.57E-05	0.000139572	3.910917151	0.001178176	0.003818517
K09007	5.46E-05	0.000156137	2.858792654	0.001208624	0.003910831
K09159	3.52E-05	0.00016561	4.705812697	0.001217824	0.003927827
K00382	2.20E-05	8.27E-05	3.762689558	0.001217824	0.003927827
K00764	0.000339796	0.000150189	0.441997005	0.001222414	0.003932528
K06374	0.000168368	0.000361719	2.14837879	0.001223234	0.003932528
K02520	0.000612944	0.000205665	0.335536585	0.001228772	0.003943961
K01182	0.000136531	0.000160697	1.177001732	0.001245129	0.003987298
K10544	4.32E-05	0.000164644	3.811784882	0.001248285	0.003987298

K14393	3.40E-05	0.00012039	3.540589931	0.001248285	0.003987298
K06867	3.39E-05	0.000121881	3.597620473	0.001254291	0.004000064
K03655	0.004247532	0.002512373	0.591490117	0.001259563	0.004010449
K02517	9.52E-05	0.000464897	4.884233598	0.00127962	0.004067801
K01777	4.97E-05	0.000439952	8.845604027	0.001291544	0.004091985
K01715	5.38E-05	0.000330914	6.151382978	0.001291544	0.004091985
K00024	0.001962185	0.001370057	0.698230495	0.001297094	0.004091985
K03695	0.00121914	0.000899367	0.73770588	0.001297211	0.004091985
K04763	0.000194726	0.000470907	2.418303147	0.001297509	0.004091985
K07304	3.56E-05	0.000105739	2.968300712	0.001303784	0.004100771
K00690	0.00063554	0.000307272	0.483481747	0.001304416	0.004100771
K02190	5.43E-05	0.000322383	5.937615545	0.001313754	0.004103227
K00857	3.08E-05	0.000275467	8.95721285	0.001315535	0.004103227
K03116	5.54E-05	0.000253675	4.578381153	0.001316063	0.004103227
K03543	2.70E-05	0.000111534	4.128600345	0.001316494	0.004103227
K03417	4.01E-05	0.000146814	3.659464977	0.001316494	0.004103227
K00849	0.000605175	0.00036781	0.607774354	0.001321032	0.004103227
K14162	5.08E-05	0.000166509	3.277117455	0.001321693	0.004103227
K13590	4.86E-05	0.000185509	3.817332148	0.001321693	0.004103227
K02356	0.002584489	0.001887653	0.730377736	0.001326728	0.004112443
K16370	4.02E-05	0.000174354	4.331814455	0.001334925	0.004131417
K05836	3.14E-05	0.000152421	4.859991697	0.001389495	0.004293625
K03798	0.000186218	0.000390548	2.097263316	0.001408259	0.004302969
K11752	0.000377463	0.000152733	0.404630807	0.001411845	0.004302969
K20534	0.000145727	0.000656661	4.506087557	0.001413477	0.004302969
K03071	4.24E-05	0.000218368	5.148959485	0.001419149	0.004302969

K09941	1.48E-05	8.85E-05	5.977252052	0.001421024	0.004302969
K02007	5.38E-05	0.000296465	5.51035108	0.001421885	0.004302969
K06940	6.01E-05	0.000292585	4.868426193	0.001424087	0.004302969
K03784	1.21E-05	0	0	0.001424953	0.004302969
K09698	1.86E-05	0	0	0.001424953	0.004302969
K00158	2.38E-05	0	0	0.001424953	0.004302969
K07146	4.03E-05	0	0	0.001424953	0.004302969
K12574	2.69E-05	0	0	0.001424953	0.004302969
K00105	1.57E-05	0	0	0.001424953	0.004302969
K07586	1.83E-05	0	0	0.001424953	0.004302969
K04094	1.50E-05	0	0	0.001424953	0.004302969
K03101	0.000130869	0.000457607	3.496688236	0.001445006	0.004356912
K02355	0.003927756	0.002907843	0.740332039	0.001471682	0.00443063
K12511	5.31E-05	0.000177664	3.343029229	0.001480597	0.004450738
K02665	5.18E-05	0.000192297	3.713532173	0.001494322	0.004478466
K02494	2.91E-05	0.000122269	4.203917714	0.001494322	0.004478466
K00971	1.54E-05	0.000180172	11.67382976	0.001497262	0.004479375
K03118	3.28E-05	0.00022609	6.887448962	0.001499127	0.004479375
K02257	3.44E-05	0.000140351	4.078909391	0.001511051	0.004494757
K16080	3.26E-05	0.000122253	3.745517847	0.001511051	0.004494757
K15725	4.89E-05	0.000191394	3.915242846	0.001511051	0.004494757
K00788	0.000258906	0.000612179	2.364483755	0.001527605	0.004530806
K02013	0.00039426	0.000686642	1.741597502	0.001527724	0.004530806
K11085	0.001097097	0.00043268	0.394386121	0.001538633	0.004556369
K19055	2.12E-05	0.000114426	5.40936816	0.001559253	0.00461057
K02535	5.11E-05	0.000154702	3.027467815	0.001566486	0.004621621

K02340	0.000234636	0.000111421	0.474865552	0.001568294	0.004621621
K02568	5.54E-05	0.000167791	3.03092556	0.001569958	0.004621621
K06187	0.000722064	0.000475603	0.658671632	0.001575049	0.004625966
K00384	0.001354103	0.001022664	0.755233053	0.001576083	0.004625966
K20712	3.82E-05	0.000136998	3.58643993	0.0015985	0.004677964
K06192	2.87E-05	0.000104511	3.641782131	0.0015985	0.004677964
K07460	0.000227044	0.000489743	2.157043412	0.001626882	0.004754031
K00351	0.000461133	0.000262788	0.569876029	0.001637125	0.004776947
K01609	3.03E-05	0.000143673	4.749117608	0.001645899	0.004788506
K06194	1.98E-05	8.92E-05	4.495276377	0.001645899	0.004788506
K13747	0.000742475	0.000334371	0.450346226	0.0016637	0.00483323
K03574	0.000182703	0.000496363	2.71677916	0.001674459	0.004857397
K03555	0.00262886	0.001977155	0.752096035	0.00168735	0.004887667
K01524	3.78E-05	0.000126828	3.353855238	0.001707628	0.004939216
K01589	1.72E-05	0	0	0.001732917	0.005002122
K01778	0.001897451	0.001447662	0.76295088	0.001734404	0.005002122
K01885	0.000711245	0.001273481	1.79049651	0.001746406	0.005029447
K02492	0.000124036	0.000422424	3.405650435	0.001749316	0.005030546
K09786	2.90E-05	0.000120287	4.150272459	0.001772479	0.005089803
K00684	2.17E-05	0.000121112	5.592149367	0.001779088	0.005101419
K06385	0.000443723	0.00018898	0.425896003	0.001785694	0.005112994
K02314	0.001792348	0.000418577	0.233535577	0.001795003	0.005122145
K00844	9.06E-05	0.000547299	6.039059762	0.001797427	0.005122145
K05796	8.07E-05	0.0004557	5.648545183	0.001797427	0.005122145
K06411	0.000177101	0.000396729	2.240130708	0.001801724	0.005122145
K03192	2.45E-05	0.000112634	4.597004823	0.00180176	0.005122145

K06048	4.20E-05	0.000149743	3.567781182	0.001815078	0.005152646
K05596	2.39E-05	0.000118138	4.940536986	0.001824909	0.005173176
K16345	3.98E-05	0.000321872	8.095930651	0.001832803	0.005188163
K00941	0.00010336	0.000197439	1.910197593	0.001841714	0.005205981
K03413	7.04E-05	0.000230222	3.26885004	0.001852433	0.005228854
K06207	0.000942076	0.000411004	0.436274507	0.00186732	0.005263408
K11189	0.000364275	0.000871094	2.391308849	0.001887068	0.005311551
K02304	2.98E-05	0.000201638	6.772736427	0.001897691	0.005333904
K15724	2.64E-05	0.000155723	5.903012144	0.001904981	0.005345646
K14761	0.000102832	0.000278441	2.707724165	0.001907241	0.005345646
K03307	0.000242492	0.001090377	4.496545014	0.001913923	0.005356832
K00338	4.41E-05	0.000170296	3.860846936	0.001927898	0.005386795
K00228	2.22E-05	0.000102182	4.611258918	0.001930043	0.005386795
K09157	0.000606984	0.000322601	0.531481979	0.001938039	0.005401537
K18955	4.26E-05	3.94E-05	0.926556761	0.001943718	0.005409789
K00906	5.09E-05	0.000154698	3.041008077	0.001957929	0.005441731
K00058	0.00030655	0.000756509	2.467814524	0.00197071	0.005469613
K00266	0.002240028	0.0013369	0.596822741	0.00203058	0.005614797
K02357	0.001899676	0.001263113	0.66490956	0.002031288	0.005614797
K01586	0.000368161	0.000692992	1.88230585	0.002031484	0.005614797
K00349	0.000936613	0.000576518	0.615534973	0.002072668	0.00572068
K01886	0.000178563	0.000463742	2.597076135	0.002088147	0.00575542
K02039	0.000970608	0.00031555	0.325105039	0.002110056	0.00580449
K01902	1.42E-05	9.13E-05	6.407136174	0.002111784	0.00580449
K03652	5.61E-06	0.000230625	41.14605838	0.002122211	0.005825102
K04564	0.000705367	0.000360239	0.510711684	0.002144294	0.005847465

K14540	0.000154511	0.000331594	2.146084429	0.002155877	0.005847465
K02948	0.006909484	0.005853781	0.847209606	0.002171952	0.005847465
K03519	0	4.62E-05	inf	0.002174429	0.005847465
K02044	0	0.000126256	inf	0.002174429	0.005847465
K16651	0	0.000148152	inf	0.002174429	0.005847465
K01042	0	0.000202555	inf	0.002174429	0.005847465
K01154	0	9.41E-05	inf	0.002174429	0.005847465
K13818	0	0.000211023	inf	0.002174429	0.005847465
K03092	0	0.000278326	inf	0.002174429	0.005847465
K09800	0	0.000170433	inf	0.002174429	0.005847465
K06934	0	0.000110084	inf	0.002174429	0.005847465
K07218	5.61E-05	0.000176961	3.152291054	0.002174434	0.005847465
K02419	5.61E-05	0.000173684	3.095884041	0.002174434	0.005847465
K05874	3.13E-05	0.000113008	3.613786865	0.002174434	0.005847465
K07137	0.000314642	0.001040937	3.308317868	0.002193627	0.005891116
K03546	3.59E-05	0.000250061	6.974481371	0.002203318	0.005909169
K03457	3.36E-05	0.000117054	3.484178202	0.00222699	0.005957273
K02428	0.000429822	0.000328484	0.764232627	0.002227242	0.005957273
K09862	1.47E-05	0.000287885	19.64333708	0.002231222	0.005959909
K08591	0.000161866	0.000559361	3.455701403	0.002245301	0.005989475
K09705	0.000108752	0.00027984	2.573192023	0.002256185	0.006010454
K03481	4.68E-05	0.000350535	7.496083635	0.002262267	0.006011328
K09790	7.54E-05	0.000207918	2.756570064	0.002262555	0.006011328
K03188	1.91E-05	7.98E-05	4.171364891	0.002267797	0.006017221
K00169	0.00014427	0.000328545	2.277294646	0.002291136	0.006071053
K00297	0.000749547	0.000428068	0.571102445	0.002296019	0.006075902

K01698	0.000102425	0.000408521	3.988507803	0.002303809	0.006088419
K01729	3.70E-05	0.000131035	3.537998335	0.002307545	0.006090204
K03976	0.000168019	0.000350343	2.085138845	0.002312954	0.006096394
K07713	0.000449612	3.60E-05	0.080177671	0.002334134	0.006144084
K14155	0.000136168	0.000529529	3.888790545	0.002346187	0.006167651
K18118	3.94E-05	0.000121843	3.091927322	0.002377128	0.006240746
K02886	0.002322437	0.001874603	0.807170607	0.002399235	0.006290483
K02058	0.000148958	0.000306811	2.059721122	0.002405617	0.006298918
K03801	4.91E-05	0.000175676	3.574494104	0.002436997	0.006371008
K00936	4.96E-05	0.000267344	5.388109634	0.002440177	0.006371008
K06891	1.73E-05	9.05E-05	5.236808954	0.002442753	0.006371008
K02032	0.000132247	0.000135659	1.02579996	0.002489794	0.006485196
K01874	0.002799057	0.002162162	0.772460842	0.002520781	0.006554482
K07720	0.000190032	0.000381867	2.009484403	0.002522982	0.006554482
K01775	5.16E-05	0.000129121	2.500753446	0.002535439	0.006563848
K09762	5.22E-05	4.90E-05	0.938018499	0.002535712	0.006563848
K01505	3.35E-05	0.000124833	3.726323779	0.002536482	0.006563848
K03802	2.19E-05	8.75E-05	3.992105221	0.002546508	0.006581234
K01304	6.67E-05	0.000226167	3.388417849	0.002560666	0.006609243
K09163	5.51E-05	0.000239581	4.347214463	0.002579386	0.006648934
K03308	0.000540854	0.000254099	0.469809472	0.002585555	0.006656215
K09955	0.000146284	0.000341432	2.334030246	0.00262814	0.006746744
K00832	6.30E-05	0.000336184	5.338394824	0.002630807	0.006746744
K02421	6.77E-05	0.000165788	2.450475068	0.002630891	0.006746744
K06387	0.000176706	0.000104806	0.59310789	0.002643877	0.006771318
K00507	2.95E-05	0.000115568	3.919322448	0.002658929	0.006801118

K05807	2.46E-05	0.000102755	4.183373402	0.002683118	0.006854177
K03566	6.51E-05	0.000257084	3.946088223	0.00270555	0.006875009
K06179	3.97E-05	0.000153091	3.854928556	0.00270555	0.006875009
K00163	3.50E-05	0.000125906	3.595018521	0.00270555	0.006875009
K05799	5.03E-05	0.000133486	2.655091128	0.002712279	0.006875009
K01633	7.96E-05	0.000278052	3.493286968	0.002715008	0.006875009
K02500	0.001724882	0.00116988	0.678237628	0.00271527	0.006875009
K10026	0.000127822	0.000460621	3.603597854	0.002715456	0.006875009
K07275	3.78E-05	0.000126866	3.358614643	0.002754533	0.006957697
K00114	3.52E-05	0.000114299	3.249631622	0.002755108	0.006957697
K02435	0.000235509	0.000632752	2.686746184	0.002764929	0.006973649
K10206	0.002537763	0.001521838	0.599676721	0.002780721	0.0070046
K03281	0.000588134	0.00026708	0.454114808	0.002788813	0.007016104
K01892	0.000727478	0.000458285	0.629963329	0.002829599	0.00701688
K04762	2.98E-05	0.000226737	7.614076056	0.002832787	0.00701688
K02527	8.69E-05	0.000398677	4.589774606	0.002834961	0.00701688
K06923	6.57E-05	0.00031158	4.744701014	0.002834961	0.00701688
K04744	6.37E-05	0.000331093	5.199932171	0.002834961	0.00701688
K08963	5.73E-05	0.000289356	5.045915935	0.002834961	0.00701688
K12506	8.18E-05	0.000338596	4.139711089	0.002834961	0.00701688
K02454	6.37E-05	0.000293985	4.614722442	0.002834961	0.00701688
K02501	0.000110862	0.000498077	4.492755225	0.002834961	0.00701688
K00872	9.48E-05	0.000366002	3.86175941	0.002834961	0.00701688
K03293	8.85E-05	0.000336477	3.802477874	0.002834961	0.00701688
K16950	6.06E-05	0.00029569	4.879889956	0.002834961	0.00701688
K22927	6.51E-05	0.000358602	5.510884609	0.002834961	0.00701688

K07042	0.000401358	0.000680585	1.695705781	0.002864909	0.007082197
K15778	3.38E-05	0.000110901	3.278240791	0.002920884	0.007211611
K06969	0.000293548	0.000658449	2.243069052	0.002933337	0.007233384
K07015	0.000315112	0.000145708	0.462400108	0.00296314	0.007297832
K00969	0.000108615	0.000431537	3.97307695	0.002968251	0.007301384
K15555	4.67E-05	0.000134834	2.8888103	0.003022205	0.007424924
K20344	0.000413802	0.000163603	0.395364889	0.003032194	0.00744028
K00001	6.84E-05	0.000369169	5.398780339	0.003053483	0.007464903
K01077	8.05E-05	0.000395914	4.917877527	0.003053483	0.007464903
K00756	7.73E-05	0.000337217	4.362540691	0.003053483	0.007464903
K03584	0.000265538	0.000551238	2.075927096	0.00306653	0.0074876
K01695	3.84E-05	0.00012098	3.149123756	0.003073531	0.007488053
K02427	3.47E-05	0.000125002	3.602688882	0.003074241	0.007488053
K02051	0.00017249	0.000343293	1.990216678	0.003078427	0.007489083
K03217	0.000920249	0.000603247	0.655526171	0.003108848	0.007540262
K09771	5.41E-05	0.000205477	3.801068194	0.003109755	0.007540262
K00481	2.66E-05	0.000103479	3.887725117	0.003110832	0.007540262
K00373	2.81E-05	0.000116673	4.151517219	0.003148431	0.007608698
K16264	4.18E-05	0.000158411	3.78618668	0.003148431	0.007608698
K00703	0.000115752	6.48E-05	0.560145253	0.003150536	0.007608698
K03048	1.15E-05	0	0	0.003161222	0.007625251
K03415	0.000112686	0.000264367	2.346048093	0.003195631	0.007698917
K03367	6.68E-06	0	0	0.003206738	0.007700828
K03366	7.55E-06	0	0	0.003206738	0.007700828
K06180	0.000283622	0.000857961	3.025016288	0.003208034	0.007700828
K07164	0.000632728	0.000382243	0.604117877	0.00322061	0.007721703

K16898	0.000137316	0.000440044	3.204610859	0.003270385	0.007831607
K03324	0.001183081	0.000788161	0.66619396	0.003320193	0.007941326
K09158	4.30E-05	0.000158228	3.682050963	0.00333415	0.007965135
K05800	3.36E-05	0.000129658	3.858307724	0.003363257	0.008025037
K01104	0.000106432	0.000594749	5.588083654	0.003462483	0.008228329
K01761	6.16E-05	0.000263376	4.272775177	0.003467802	0.008228329
K03673	5.55E-05	0.000182187	3.283751971	0.003473657	0.008228329
K17323	5.17E-05	0.000150793	2.916939607	0.003474308	0.008228329
K02394	1.96E-05	0.000104474	5.32990183	0.003486287	0.008228329
K05921	1.97E-05	0.000107822	5.476935898	0.003486287	0.008228329
K17236	8.61E-05	0.000210939	2.450015406	0.003486776	0.008228329
K01895	0.00157717	0.000546223	0.346331073	0.003489009	0.008228329
K14682	3.53E-05	0.00010449	2.961002102	0.003489018	0.008228329
K03549	3.01E-05	0.000104928	3.490473862	0.003489804	0.008228329
K02837	6.35E-05	0.000155058	2.441953036	0.003498015	0.008237928
K03609	6.80E-05	1.81E-05	0.266680647	0.003507573	0.008250374
K00595	8.76E-05	0.00022183	2.531121329	0.003513578	0.008250374
K19117	4.34E-05	0.000151451	3.490119617	0.003515737	0.008250374
K01308	0.000108405	0.00026724	2.465201313	0.003530271	0.008274722
K09774	7.78E-05	0.000397413	5.111299673	0.003537242	0.008281307
K00333	4.54E-05	0.000134093	2.954801401	0.003541883	0.00828243
K05832	0.000710939	0.000273634	0.384890383	0.003557202	0.008308488
K07718	0.00016015	0.000330151	2.061515736	0.00357143	0.00833194
K06381	4.79E-05	0.000249801	5.217229796	0.003594565	0.008376094
K04047	1.53E-05	0	0	0.003603793	0.008377977
K01582	1.89E-05	0	0	0.003603793	0.008377977

K01669	3.04E-05	0.00013316	4.386986932	0.00361896	0.008403418
K00945	0.000152311	0.000535298	3.514498988	0.003656456	0.00848059
K03769	5.20E-05	0.000140743	2.706962665	0.003678994	0.008522931
K04488	0.000507459	0.000730868	1.440251934	0.003742639	0.008660293
K02069	0.000236606	0.000121641	0.514108549	0.003753238	0.008674731
K00978	0.000154016	8.27E-05	0.536658448	0.003776499	0.008718368
K08161	0.00013039	0.000424519	3.255773652	0.003804396	0.008742204
K03699	6.54E-05	0.00036479	5.577744257	0.003804396	0.008742204
K09807	6.96E-05	0.000232924	3.346760825	0.003804396	0.008742204
K08223	6.21E-05	0.000295244	4.751570547	0.003804396	0.008742204
K03789	0.000163451	0.000411434	2.517171937	0.00388606	0.008919561
K19624	4.30E-05	0.000133683	3.106224354	0.003894585	0.00892883
K02040	0.000646525	0.000316394	0.489375678	0.003914691	0.008964597
K00413	3.72E-05	0.00013059	3.507729639	0.003920892	0.008968478
K02825	6.53E-05	0.000197603	3.025537097	0.003932187	0.008983986
K07478	7.28E-05	0.000247426	3.396826482	0.003951717	0.009007041
K06879	3.85E-05	0.000140633	3.654875161	0.003954119	0.009007041
K02231	5.71E-05	0.000164367	2.878599891	0.003955856	0.009007041
K14645	4.27E-05	0.00014064	3.297297434	0.003969428	0.009027613
K00240	0.001100503	0.000683823	0.621373743	0.003990739	0.009065721
K02006	8.03E-05	0.000324117	4.036041167	0.004089761	0.009258959
K15580	9.48E-05	0.000362049	3.818925636	0.004089761	0.009258959
K07459	7.14E-05	0.000302116	4.2327503	0.004089761	0.009258959
K06137	4.61E-05	0.000182816	3.967322023	0.004096289	0.009263199
K00449	3.28E-05	0.000111669	3.399760697	0.004120845	0.009308152
K06518	0.002855102	0.000708383	0.248111139	0.004137966	0.009336226

K17234	9.59E-05	0.000238271	2.484100761	0.004180189	0.009401696
K05989	0.000124783	0.000293607	2.35293742	0.00418093	0.009401696
K02068	8.30E-05	0.000230835	2.782779197	0.004181156	0.009401696
K03785	4.72E-05	0.000272411	5.770355192	0.004198971	0.009424593
K07456	0.000710236	0.000307036	0.432300561	0.004200811	0.009424593
K01772	3.67E-05	0.0001336	3.638057962	0.004207181	0.009428254
K07005	0.000133488	0.000297436	2.228178251	0.004234822	0.009479522
K01878	1.54E-05	0.00010142	6.569959628	0.004251212	0.009505518
K07052	0.000458853	0.0002022	0.440664991	0.004259491	0.009513341
K02047	0.000532313	0.000231497	0.434889114	0.004304566	0.009603236
K02115	0.000428126	0.00073357	1.713444966	0.004358102	0.009707645
K01658	2.82E-06	0.000153182	54.30735672	0.004361123	0.009707645
K06997	0.000215909	0.000111739	0.517526181	0.004385471	0.009750936
K00366	8.45E-05	0.000313024	3.706278404	0.00439442	0.009759928
K00332	9.96E-06	4.84E-05	4.854632645	0.00441558	0.009795992
K14731	3.27E-05	0.000114919	3.515117125	0.004445352	0.009851059
K00568	4.02E-05	0.000125434	3.123911947	0.004475806	0.009907513
K03536	0.000130017	0.000342918	2.637489167	0.004505338	0.009961802
K18122	2.76E-05	0.000134116	4.851759635	0.004568548	0.010090355
K01372	0.000735414	0.000508233	0.691084998	0.004598014	0.010144178
K05550	3.72E-05	0.00012589	3.38592339	0.004604502	0.010147242
K00688	0.000453944	0.000233167	0.513645632	0.004622515	0.010175669
K07112	0.000191213	0.000402862	2.106876684	0.004672518	0.010274376
K06023	0.000446395	0.000614414	1.376388869	0.004700644	0.010324814
K01624	0.000243312	0.000168663	0.693195418	0.004709264	0.010332344
K19302	0.000126641	0.000553263	4.36873	0.004728224	0.010362517

K02037	0.000413138	0.000647791	1.567978842	0.004757335	0.010404662
K01903	3.77E-05	0.000122503	3.248288527	0.004757911	0.010404662
K09685	1.78E-05	0	0	0.004808563	0.010503886
K12510	0.000407344	0.000212622	0.521972699	0.004889577	0.010639956
K01819	1.46E-05	0	0	0.004892241	0.010639956
K01916	1.17E-05	0	0	0.004892241	0.010639956
K15986	1.76E-05	0	0	0.004892241	0.010639956
K00963	5.04E-05	0.000130927	2.595819085	0.004905911	0.010658038
K07006	4.51E-05	0.000170423	3.778667137	0.00491661	0.010664743
K00370	3.18E-05	9.64E-05	3.035626723	0.004919716	0.010664743
K01696	0.001858397	0.001345029	0.723757643	0.004947708	0.010668981
K07341	0	8.44E-05	inf	0.004948477	0.010668981
K01223	0	0.000146155	inf	0.004948477	0.010668981
K02483	0	2.80E-05	inf	0.004948477	0.010668981
K21449	0	0.000122776	inf	0.004948477	0.010668981
K08317	3.67E-05	0	0	0.005000169	0.010722345
K09952	3.76E-05	0	0	0.005000169	0.010722345
K01760	4.97E-05	0	0	0.005000169	0.010722345
K07075	1.97E-05	0	0	0.005000169	0.010722345
K03598	7.97E-05	0	0	0.005000169	0.010722345
K01488	0.000752018	0.000379169	0.504202671	0.00500901	0.010729742
K00957	0.002493206	0.001836907	0.736764852	0.005025559	0.010744626
K03526	0.001083191	0.000754412	0.696471652	0.005026757	0.010744626
K05592	6.85E-05	0.000298268	4.351264661	0.005047464	0.010777309
K21929	0.000107489	0.000415397	3.864563578	0.005066213	0.01080575
K03743	0.000763464	0.000486803	0.637623368	0.00508957	0.010843945

K03814	4.65E-05	0.000142806	3.069003455	0.005153736	0.010968913
K03665	0.001642781	0.000543169	0.330639698	0.005192614	0.011039852
K03498	0.000138033	0.000548175	3.971326087	0.005238574	0.011125681
K00561	0.003358198	0.00377002	1.122631844	0.005289725	0.011222338
K02804	0.000642957	0.000339141	0.527470707	0.005324263	0.011271577
K02803	0.000642957	0.000339141	0.527470707	0.005324263	0.011271577
K02750	9.64E-05	0.000424041	4.39998542	0.005363006	0.011329492
K02749	9.64E-05	0.000424041	4.39998542	0.005363006	0.011329492
K03327	4.28E-05	0.000126091	2.942875921	0.005372969	0.011338503
K07078	8.94E-05	0.000325018	3.63643278	0.005435784	0.011441946
K22305	0.000130645	0.000458239	3.507506185	0.005435784	0.011441946
K10537	3.38E-05	0.000114473	3.391752909	0.005439237	0.011441946
K17103	4.15E-05	0.000122816	2.960146897	0.005451865	0.011456401
K19299	0.000238501	4.83E-05	0.202368866	0.005473593	0.011489926
K06133	0.000270621	0.000146125	0.539960566	0.005499631	0.011532419
K01628	0.000203483	0.000130277	0.640237864	0.005530148	0.011584205
K00598	1.13E-05	6.41E-05	5.677636581	0.00555138	0.011604251
K02509	1.10E-05	5.46E-05	4.948698754	0.00555138	0.011604251
K03977	0.00206417	0.001453563	0.704187391	0.005574259	0.011639849
K01783	0.000147626	0.000456794	3.094274676	0.005659211	0.011804853
K09019	4.43E-05	0.000163463	3.694061681	0.00566594	0.011806513
K11927	1.35E-05	5.46E-05	4.034456139	0.005679375	0.01182213
K00005	6.91E-05	0.000332683	4.812490399	0.005744621	0.01194545
K08311	5.92E-05	0.000152986	2.583802585	0.005754332	0.011953154
K05946	0.000213896	0.000558827	2.612608882	0.005833398	0.012104758
K01629	0.000200709	0.000357667	1.782019705	0.005880109	0.012188976

K03106	0.000177541	0.000126651	0.713358474	0.005919062	0.012256954
K09129	4.09E-05	0.000112578	2.75434465	0.006014992	0.012442655
K07813	0.00057043	0.001392471	2.441089932	0.006069186	0.012541724
K02197	4.46E-05	0.000130994	2.937791602	0.006139542	0.012656585
K01451	3.38E-05	0.00011306	3.348517073	0.006141391	0.012656585
K02001	0.000186882	0.000123389	0.660249801	0.00614385	0.012656585
K07127	3.10E-05	0.000117912	3.801556832	0.00615504	0.012657624
K07173	3.96E-05	7.59E-06	0.191645342	0.00617754	0.012657624
K02588	0.00010259	4.30E-05	0.419252187	0.00618329	0.012657624
K07035	0.000870161	0.000425968	0.489527809	0.006187635	0.012657624
K20117	0.000189528	0.000505329	2.6662568	0.006188879	0.012657624
K20118	0.000189528	0.000505329	2.6662568	0.006188879	0.012657624
K20116	0.000189528	0.000505329	2.6662568	0.006188879	0.012657624
K06139	3.49E-05	0.000110068	3.155954416	0.006210514	0.012688833
K08289	0.002149587	0.001370476	0.637552949	0.006249221	0.01275482
K06131	1.94E-05	7.10E-05	3.65514154	0.006282525	0.012809657
K22010	0.000127274	0.00032045	2.517796026	0.006308981	0.012850432
K09001	7.54E-05	0.000186869	2.479258492	0.006519736	0.013266129
K01870	0.000263313	0.000580915	2.206178153	0.006562894	0.013340305
K14652	0.001686555	0.000853169	0.505865044	0.006575344	0.013351974
K01028	4.22E-05	0.000126745	3.006651574	0.00660366	0.013369749
K01724	5.68E-05	0.000222821	3.923665304	0.00660366	0.013369749
K08084	5.58E-05	0.000180096	3.226892473	0.006605617	0.013369749
K00096	0.000630209	0.000286657	0.454860514	0.006617895	0.013369749
K01092	3.72E-05	0.000121878	3.274239578	0.006629537	0.013369749
K18979	2.69E-05	9.74E-05	3.617115208	0.006629537	0.013369749

K02195	3.51E-05	0.000104663	2.983088676	0.006631127	0.013369749
K01915	0.002943176	0.00191275	0.649893274	0.006668789	0.013432075
K01151	9.43E-05	0.00033435	3.547379503	0.006695015	0.013457656
K09963	9.12E-05	0.000359216	3.938404917	0.006695015	0.013457656
K02557	8.19E-05	0.000184826	2.256608214	0.006764559	0.013583727
K02935	0.003055653	0.002209855	0.723202237	0.00678863	0.013604607
K02965	0.006782138	0.005298252	0.781206759	0.00678863	0.013604607
K15583	7.83E-05	0.000354118	4.524708196	0.006812318	0.013638342
K02189	0.00015722	0.000347419	2.209762999	0.006871478	0.013730809
K01596	5.83E-05	0.00014137	2.424290017	0.006872304	0.013730809
K15770	0.000108406	0.000242841	2.240106746	0.006927034	0.013823927
K06921	2.32E-05	0.000270233	11.62735474	0.006932804	0.013823927
K02343	0.000120384	0.000427969	3.555026501	0.007053	0.014046081
K03826	1.93E-05	2.57E-06	0.133531942	0.007058332	0.014046081
K03530	0.001764819	0.001042065	0.590465528	0.007067929	0.014051128
K02188	0.000171812	0.000451926	2.630355701	0.007078421	0.014057943
K03154	7.36E-05	0.000213674	2.902064699	0.00709909	0.014075007
K03274	2.67E-05	8.04E-05	3.014517278	0.007101159	0.014075007
K01243	0.000111638	0.000285079	2.553602466	0.007119353	0.014097026
K07002	2.89E-05	9.71E-05	3.36655725	0.007141304	0.014121838
K07448	9.67E-05	4.32E-05	0.446662577	0.007146076	0.014121838
K04654	0.000140014	0.000138307	0.987814712	0.007167604	0.014150329
K02836	7.81E-05	7.61E-05	0.973948875	0.007218016	0.014235731
K06975	0.000124547	0.000315937	2.536686672	0.007299953	0.014383076
K02950	0.005245626	0.007036991	1.341496778	0.00731539	0.014399235
K08301	0.000150591	0.000405179	2.690583915	0.00733167	0.014417018

K03466	0.000221817	0.000591701	2.667518812	0.007367696	0.014432543
K16509	1.83E-05	0	0	0.007368575	0.014432543
K01635	1.43E-05	0	0	0.007368575	0.014432543
K05823	1.75E-05	0	0	0.007368575	0.014432543
K05589	5.46E-05	0.000157853	2.888973259	0.007398039	0.014476005
K07443	0.000145536	0.000417103	2.865974629	0.007452594	0.014568431
K01267	0.000264826	0.000510662	1.928291149	0.007582383	0.014807598
K03545	0.000703372	0.000465299	0.661525998	0.007600714	0.014828845
K00677	0.000522634	0.000329384	0.630238385	0.007627815	0.014867141
K01436	0.000103782	0.00044036	4.243142394	0.007659301	0.014913903
K03272	0.000110952	0.000345221	3.111453542	0.007674334	0.014928567
K01258	3.53E-05	3.31E-06	0.093543985	0.00770106	0.014965927
K02123	0.000370793	0.000255904	0.690152974	0.007721683	0.014991366
K00111	0.000193289	0.000363924	1.882798365	0.007737878	0.015008164
K03547	0.000155257	0.000342046	2.203100314	0.007777093	0.015069539
K03529	0.000158715	0.000426636	2.688058641	0.007788516	0.015076991
K01653	0.000117108	5.67E-05	0.484307042	0.007818033	0.015119422
K03634	2.38E-05	8.57E-05	3.59894148	0.007829981	0.015127827
K01858	7.65E-05	5.51E-05	0.72089973	0.007842711	0.015137726
K06310	0.000161996	0.000319287	1.970956685	0.007868203	0.015172213
K01876	0.002638525	0.001873839	0.710184176	0.007878535	0.01517743
K00549	7.64E-05	7.18E-05	0.939623897	0.00789364	0.015191821
K03650	0.000108592	0.000464197	4.274697232	0.008079341	0.015534192
K03427	0.000163109	0.000367772	2.254763965	0.008088088	0.015535998
K02656	2.81E-05	9.67E-05	3.444059514	0.00809842	0.015540846
K02652	3.67E-05	0.000112481	3.066842385	0.008131745	0.015589763

K08217	0.00016469	8.57E-05	0.520249138	0.008145745	0.015598461
K06966	3.98E-05	0.000170782	4.295405852	0.008151959	0.015598461
K01615	0.000117707	5.66E-05	0.480624697	0.008161156	0.015601058
K10831	3.35E-05	9.45E-05	2.823365176	0.008193504	0.015647863
K02405	0.000110168	0.0002347	2.130380306	0.008258683	0.015757219
K01657	0.001216946	0.000871899	0.716464385	0.008360879	0.015924407
K07089	5.92E-06	0	0	0.008386321	0.015924407
K01512	1.35E-05	0	0	0.008386321	0.015924407
K03095	1.21E-05	0	0	0.008386321	0.015924407
K02761	8.85E-06	0	0	0.008386321	0.015924407
K07010	4.75E-05	5.31E-05	1.117831749	0.008403369	0.015941568
K03394	0.00011597	4.93E-05	0.424924092	0.008514293	0.016128457
K03311	7.41E-05	0.000293724	3.962844396	0.008518095	0.016128457
K12257	0.001478148	0.000745301	0.504212972	0.008535102	0.016145297
K03786	5.38E-05	0.000152628	2.836361693	0.008579481	0.016213834
K07584	0.000163312	5.90E-05	0.361019165	0.008599907	0.016237017
K06909	0.000118967	3.46E-05	0.291199694	0.008624498	0.01626801
K07009	1.95E-05	5.30E-05	2.709404787	0.008635818	0.016273937
K05541	3.41E-05	0.000101922	2.993245485	0.0086657	0.016314799
K06397	0.000129116	0.000295171	2.286095367	0.008721434	0.01640421
K20265	0.000563869	0.00026069	0.462322884	0.008735475	0.016415103
K03410	9.36E-05	0.000215029	2.296360184	0.008754344	0.016435042
K05601	0.000540176	0.000358111	0.662951681	0.008772372	0.016444242
K07012	4.37E-05	0.000178328	4.076136191	0.008775771	0.016444242
K00374	4.75E-05	0.000128871	2.711229229	0.008791727	0.016458642
K02668	1.15E-05	6.23E-05	5.422357768	0.008827615	0.016476697

K09858	1.50E-05	4.67E-05	3.124828885	0.008827615	0.016476697
K03424	0.000184911	0.000565851	3.060129767	0.008829444	0.016476697
K00340	2.41E-05	0.000126822	5.263470438	0.00883449	0.016476697
K02654	0.000604467	0.002214386	3.663371825	0.009034058	0.016833123
K01996	0.000301824	0.000472842	1.566616454	0.00905003	0.016842407
K09768	0.000114147	0.000199228	1.745362383	0.009055968	0.016842407
K15012	4.62E-05	0.00013868	3.002297684	0.009142946	0.016988293
K00604	0.000607094	0.000863974	1.423129996	0.009217444	0.017110741
K03686	0.001258384	0.000722457	0.574114487	0.00923211	0.017121994
K00948	0.000320301	0.000589382	1.840087819	0.009281577	0.017197708
K00666	0.0001088	0.00018373	1.688692219	0.009359335	0.017325654
K05805	4.01E-05	0.000134721	3.363478289	0.009375602	0.017339635
K03601	8.23E-05	0.000326661	3.971218072	0.009384749	0.017340437
K01918	0.000751153	0.001094498	1.457091536	0.009400674	0.017353748
K05350	0.000153712	0.000323969	2.107632315	0.009480211	0.017484357
K02744	5.42E-05	2.55E-06	0.047032977	0.009514344	0.017531061
K00016	6.66E-05	7.19E-05	1.079254346	0.009577354	0.017630837
K07386	0.00010225	0.000356434	3.485906627	0.009607578	0.01767013
K02408	0.000121414	0.000275347	2.267827387	0.009637935	0.017709595
K03614	0.000650842	0.000395896	0.608283009	0.009689097	0.01778718
K05813	0.000128841	0.000264791	2.055167795	0.009742043	0.017867895
K03151	0.000562819	0.000277661	0.493340351	0.009772487	0.017907227
K00790	0.001093242	0.000783515	0.716689358	0.00982331	0.017983797
K02111	0.00404183	0.003268338	0.808628372	0.009950656	0.018200188
K03742	5.10E-05	0.000302849	5.943258094	0.010041709	0.018349863
K03706	0.00012985	0.000222687	1.714957794	0.010053106	0.018353836

K03189	3.20E-05	0.000101689	3.176718833	0.010140343	0.018496135
K07040	0.00015125	0.000450864	2.980928777	0.010211168	0.018608264
K01462	0.002305425	0.001677259	0.727527067	0.010240827	0.018645238
K03269	0.000449748	0.000291174	0.647416639	0.01039951	0.018916841
K09767	3.80E-05	0.000115604	3.043446791	0.010413891	0.018925702
K01923	0.000997514	0.000755932	0.757816382	0.010538269	0.019134265
K01704	0.000388052	0.000607055	1.564365025	0.010580201	0.01919289
K01155	1.79E-05	0.000112042	6.2702425	0.010627063	0.019260342
K21345	9.83E-07	5.25E-05	53.3914692	0.010654537	0.019275026
K18345	1.56E-06	3.15E-05	20.18845307	0.010654537	0.019275026
K03722	3.35E-05	9.74E-05	2.90945863	0.010724266	0.019383551
K00926	6.91E-05	2.73E-05	0.395169558	0.010771929	0.019452031
K07560	0.000201309	0.000332704	1.652699486	0.010813095	0.019508666
K13010	4.50E-05	0.000118914	2.639863215	0.01083906	0.019537799
K16692	3.15E-06	4.81E-05	15.2995192	0.010850652	0.019540993
K00859	0.000105709	0.000396852	3.754206316	0.010887413	0.019589469
K06191	8.93E-06	0	0	0.010909498	0.019593774
K02795	9.13E-06	0	0	0.010909498	0.019593774
K07085	0.002147248	0.001309954	0.610061731	0.010944518	0.019638945
K00528	0.000826464	0.000467648	0.565841657	0.011028136	0.019769674
K07027	0.000127145	0.000260687	2.050310608	0.011047314	0.019769674
K06972	0.000137824	0.000361505	2.622952441	0.011053345	0.019769674
K04651	9.95E-05	0.000273297	2.746082282	0.011057109	0.019769674
K00448	2.30E-05	6.66E-05	2.899438328	0.011154298	0.019925541
K00364	1.28E-05	0	0	0.011209733	0.019956021
K03078	8.18E-06	0	0	0.011209733	0.019956021

K00226	7.04E-06	0	0	0.011209733	0.019956021
K06173	0.001118163	0.000802674	0.717850762	0.011305233	0.019956021
K03385	0	4.53E-05	inf	0.011412036	0.019956021
K19130	0	8.38E-05	inf	0.011412036	0.019956021
K19129	0	6.29E-05	inf	0.011412036	0.019956021
K03781	0	4.75E-05	inf	0.011412036	0.019956021
K01442	0	5.29E-05	inf	0.011412036	0.019956021
K19127	0	7.25E-05	inf	0.011412036	0.019956021
K19128	0	7.56E-05	inf	0.011412036	0.019956021
K14988	0	1.18E-05	inf	0.011412036	0.019956021
K03386	0	4.21E-05	inf	0.011412036	0.019956021
K01572	0	4.28E-05	inf	0.011412036	0.019956021
K06911	0	2.69E-05	inf	0.011412036	0.019956021
K20444	0	1.91E-05	inf	0.011412036	0.019956021
K00259	0	3.77E-05	inf	0.011412036	0.019956021
K15987	0	4.45E-05	inf	0.011412036	0.019956021
K07106	0	5.19E-05	inf	0.011412036	0.019956021
K18707	0	6.64E-05	inf	0.011412036	0.019956021
K06223	0	2.97E-05	inf	0.011412036	0.019956021
K18785	0	5.19E-05	inf	0.011412036	0.019956021
K01639	0	3.78E-05	inf	0.011412036	0.019956021
K01089	0	3.17E-05	inf	0.011412036	0.019956021
K09777	0.000105898	0.000294843	2.784220059	0.0114274	0.019965344
K07305	5.53E-05	0.000152717	2.76073658	0.011457581	0.020000514
K03442	0.000769338	0.000517051	0.672072715	0.011531468	0.02011185
K01644	3.80E-05	0.000106119	2.789277445	0.011714779	0.020413669

K00641	3.93E-05	0.000102545	2.610780962	0.011728554	0.020414139
K01215	3.35E-05	5.28E-06	0.157320327	0.011735565	0.020414139
K01814	0.000903021	0.000748649	0.829049582	0.011772348	0.020460237
K02926	0.001944886	0.001591685	0.81839488	0.011810031	0.02050782
K02914	0.041227684	0.02962996	0.718690868	0.011873396	0.020599875
K03637	0.000265708	0.000756043	2.845396783	0.011945996	0.02069991
K01711	0.002178857	0.001590378	0.72991409	0.011951858	0.02069991
K02601	0.000120215	8.63E-05	0.718249371	0.01198667	0.02074215
K01095	1.92E-05	3.26E-06	0.169488057	0.012005083	0.020755965
K02401	9.70E-05	0.000204363	2.107554139	0.012067328	0.020845471
K18889	0.000384797	0.000206518	0.536693554	0.012208857	0.021066896
K07301	0.00022747	0.000474	2.083786816	0.012218524	0.021066896
K01507	8.34E-05	0.000156499	1.875965055	0.012230687	0.021066896
K03559	0.00118071	0.000772795	0.654517385	0.012237855	0.021066896
K02346	0.000142372	0.000444201	3.119992634	0.012449573	0.021412835
K04487	0.000112926	0.000241298	2.136772882	0.012523573	0.021521511
K10117	0.000528267	0.000452134	0.855881264	0.012633043	0.021690903
K01867	0.001154845	0.000482713	0.417988892	0.012767327	0.021902569
K03408	8.15E-05	0.000192737	2.365418703	0.012803002	0.021944853
K19689	0.000103497	0.000326226	3.152020806	0.012821839	0.021958226
K06398	0.00030468	0.000192977	0.633378062	0.013025901	0.022288516
K00164	4.05E-05	9.33E-05	2.306685696	0.013254014	0.022659354
K05985	7.82E-05	0.000312757	4.000888998	0.013343997	0.022793609
K06952	0.000355851	0.000605545	1.701682234	0.013375245	0.022827391
K03648	0.000663574	0.000451388	0.680237755	0.013445078	0.022916034
K10823	0.000114824	0.000384398	3.347725534	0.013450215	0.022916034

K01962	0.000723805	0.000368135	0.508611001	0.013537214	0.02304453
K06403	0.000357394	0.000202396	0.566310637	0.013560411	0.023064289
K00067	6.28E-05	0.000173825	2.766230453	0.013987974	0.023771195
K15577	2.97E-05	9.53E-05	3.209403418	0.014000075	0.023771459
K00537	2.02E-05	0	0	0.014058605	0.023846956
K09806	5.17E-05	0.00015971	3.086610728	0.014076321	0.023846956
K00012	2.91E-05	0.000113501	3.906299167	0.014080489	0.023846956
K00702	8.69E-05	3.45E-05	0.397621116	0.014107074	0.023871664
K16137	4.73E-05	0.000117978	2.493168686	0.014214819	0.024033173
K09810	0.002086527	0.0007535	0.361126574	0.014226672	0.024033173
K03495	0.001249625	0.00089675	0.717615643	0.014413485	0.024328104
K01200	0.000298333	0.0001504	0.504133863	0.01449125	0.024438633
K07736	0.000276916	0.0004768	1.721820688	0.014723475	0.024809243
K07335	0.000116767	0.00026007	2.227264963	0.014749672	0.024832358
K18475	0.000119711	6.93E-05	0.579154311	0.014843612	0.024969389
K06857	3.11E-05	9.04E-05	2.907502088	0.014902279	0.025046904
K01153	6.80E-06	0.00017121	25.19642729	0.014949259	0.025104663
K02334	0.000107721	7.03E-05	0.652961236	0.015207762	0.02551724
K05896	8.90E-05	0.000345193	3.877918646	0.015297762	0.025646627
K10192	9.44E-05	0.000206293	2.18638838	0.01534629	0.025706327
K02112	0.003899671	0.003166952	0.812107332	0.015387164	0.025753118
K06406	0.000368763	0.000217688	0.590320245	0.015415086	0.02577817
K02919	0.000555079	0.000113041	0.20364868	0.01547161	0.025850968
K02030	0.001345915	0.001771369	1.316107293	0.015545078	0.025951934
K00940	2.99E-05	9.45E-05	3.161172593	0.015620427	0.026055868
K04655	0.000169873	7.90E-05	0.46488293	0.015751482	0.026252469

K01686	0.000811429	0.000378759	0.466779883	0.015782746	0.026282564
K11962	4.17E-05	0.000100427	2.4082551	0.015866417	0.026399807
K11928	7.36E-05	6.41E-05	0.871130521	0.015914106	0.026421569
K07503	7.10E-05	6.09E-05	0.857540628	0.015914106	0.026421569
K02388	0.000110137	0.000234465	2.128846729	0.015926774	0.026421569
K07699	0.000218707	0.000334395	1.528962819	0.01595524	0.026421569
K01821	3.79E-05	0.000125267	3.303819606	0.015970613	0.026421569
K01661	2.28E-05	0.000302881	13.27322535	0.015975928	0.026421569
K03310	0.002370609	0.001604231	0.676717074	0.015983629	0.026421569
K18929	1.03E-05	4.07E-05	3.943524444	0.01599899	0.026421569
K00254	4.03E-06	3.08E-05	7.660883096	0.01599899	0.026421569
K07090	0.000258798	0.000472704	1.826540457	0.016059784	0.026499975
K07979	0.000163589	0.000332602	2.033155032	0.016191282	0.026679436
K08744	2.51E-05	8.66E-05	3.456092367	0.016196817	0.026679436
K02895	0.002853707	0.002138725	0.749455237	0.016208763	0.026679436
K02498	2.40E-05	6.47E-05	2.698678413	0.016316609	0.026834754
K04095	2.18E-05	4.53E-05	2.078049522	0.016403577	0.026955507
K01697	0.000103433	7.02E-05	0.678655987	0.016494589	0.027060372
K01201	7.57E-05	6.49E-05	0.857639572	0.016494589	0.027060372
K03110	6.89E-05	6.72E-05	0.974746106	0.016531801	0.027076777
K10805	0.000112612	5.61E-05	0.498256099	0.016531801	0.027076777
K00014	0.000168087	0.000468229	2.785633674	0.016622945	0.027203668
K01802	3.52E-05	6.10E-05	1.734017018	0.016676366	0.027268668
K00547	0.000111954	0.000337835	3.017631175	0.016728174	0.027330925
K15771	0.000122281	0.000283829	2.321122406	0.016758226	0.027357564
K08681	0.000214619	0.0001902	0.886224473	0.016847149	0.027463537

K01224	7.83E-05	1.75E-05	0.223283186	0.016946716	0.027463537
K14983	6.62E-06	0	0	0.016947348	0.027463537
K09976	1.56E-05	0	0	0.016947348	0.027463537
K02075	1.18E-05	0	0	0.016947348	0.027463537
K01252	1.28E-05	0	0	0.016947348	0.027463537
K22336	1.57E-05	0	0	0.016947348	0.027463537
K01823	1.40E-05	0	0	0.016947348	0.027463537
K03212	1.37E-05	0	0	0.016947348	0.027463537
K09974	0.000439054	0.000255474	0.581873972	0.017014172	0.027549391
K01856	2.14E-05	7.60E-05	3.546233724	0.01717784	0.027791791
K00324	8.91E-05	0.000196604	2.207790213	0.017245298	0.027878264
K01626	0.000117471	0.000126035	1.072905359	0.017295636	0.027914288
K07862	3.53E-05	6.47E-05	1.829605858	0.017295636	0.027914288
K01607	2.86E-05	0.000130579	4.570222581	0.017434273	0.028106478
K07644	1.94E-05	6.88E-05	3.537080645	0.017457089	0.028106478
K01430	2.84E-05	0.000126248	4.440294821	0.017457089	0.028106478
K04077	0.0022302	0.001803325	0.808593091	0.017867733	0.028744373
K10008	8.55E-05	8.18E-05	0.95575924	0.01802223	0.028946116
K16148	5.40E-05	6.40E-05	1.184226724	0.01802223	0.028946116
K00868	0.000327072	0.000752943	2.302071661	0.018123132	0.029084704
K03284	0.00016636	0.000308715	1.855705049	0.01839528	0.029497669
K02054	0.000131441	0.000114209	0.868901704	0.018457784	0.029574066
K14058	5.42E-05	0.000129634	2.393868777	0.018490832	0.029603182
K14170	0.000161904	0.00048319	2.984425427	0.018773616	0.030031749
K02529	0.000764796	0.001158232	1.514433327	0.018857882	0.030098697
K09773	1.58E-05	7.10E-05	4.493755329	0.018860842	0.030098697

K22292	1.90E-05	6.97E-05	3.67818501	0.018860842	0.030098697
K02035	0.000796826	0.002172885	2.726925955	0.019101892	0.030438985
K02835	0.001780314	0.001556917	0.874518356	0.019106445	0.030438985
K01934	0.00019131	0.000458082	2.394453728	0.019125667	0.030438985
K11145	6.43E-05	0.000133099	2.069245253	0.019135261	0.030438985
K03612	9.32E-05	0.000197882	2.123843215	0.019151061	0.030439786
K09825	0.000226764	0.000158781	0.700201705	0.019471354	0.030924177
K00175	0.00094842	0.000617361	0.650935546	0.019607734	0.031115941
K00609	0.001840269	0.001439237	0.782079412	0.019642117	0.031145668
K01537	0.000240619	0.000343521	1.427658494	0.019942535	0.03159685
K17318	0.000247947	0.000446713	1.801649112	0.019975329	0.031623632
K00145	0.003748219	0.003265321	0.871165974	0.020151183	0.031876672
K03698	0.00016423	0.000156778	0.954621471	0.020222549	0.031964155
K06925	0.000226796	0.00045727	2.016222404	0.020386988	0.032198496
K00794	0.0025515	0.001947062	0.763105081	0.02041998	0.032225028
K18344	1.97E-06	3.33E-05	16.89939007	0.020543853	0.032394824
K01928	0.000937937	0.000799008	0.851878371	0.021013272	0.033108798
K03711	0.000648563	0.000915706	1.411900845	0.021171928	0.033332386
K00796	0.000111022	0.000247379	2.228190266	0.021434775	0.033719528
K02103	0.00012523	0.000246741	1.970295469	0.021632222	0.034003255
K11175	5.55E-05	0.000141156	2.545107087	0.021774423	0.034199764
K10006	6.83E-05	5.69E-05	0.832942563	0.021922307	0.034404883
K06334	0.000196786	0.000346553	1.761065203	0.021988744	0.034481955
K02888	0.002469585	0.001862658	0.754239219	0.022098746	0.034627169
K19506	1.08E-05	5.89E-05	5.455327409	0.022230405	0.034806063
K06896	7.87E-05	1.37E-05	0.174046173	0.022576419	0.035320026

K03497	0.001249141	0.002210768	1.769830353	0.02268525	0.035461485
K09015	3.88E-05	4.66E-05	1.199704967	0.022720298	0.035461485
K02503	5.90E-05	5.49E-05	0.929997257	0.022720298	0.035461485
K17686	0.000183603	0.000510655	2.781303613	0.022869568	0.03566649
K04750	4.94E-05	0.000135817	2.746856481	0.02290757	0.03569778
K21757	3.79E-05	8.71E-05	2.299099864	0.022947278	0.035731678
K01921	9.30E-05	0.000233487	2.511040498	0.023136838	0.035998676
K13052	7.97E-06	0	0	0.023544102	0.036546618
K07742	9.21E-06	0	0	0.023544102	0.036546618
K06198	4.63E-06	0	0	0.023544102	0.036546618
K02992	0.002546594	0.002013599	0.790703126	0.023588512	0.036570455
K00215	7.55E-05	0.000147392	1.951360381	0.023596213	0.036570455
K05970	8.96E-05	6.30E-05	0.702696298	0.024187219	0.03745725
K02899	0.003610916	0.0030265	0.838153007	0.024209355	0.037462377
K01693	0.00030588	0.000480158	1.569757404	0.024274453	0.037533925
K10974	5.31E-05	0.000168298	3.171633679	0.024337993	0.037602955
K03502	0.000350379	0.00068052	1.942237568	0.024478364	0.037789179
K21990	4.59E-05	0.00014091	3.067571134	0.024496503	0.037789179
K01799	3.70E-05	7.88E-05	2.132546974	0.024573008	0.037877836
K04042	7.75E-05	0.00018252	2.355460054	0.024655397	0.037975418
K00620	9.82E-05	0.000230797	2.351333732	0.025022131	0.038510472
K02982	0.002243882	0.001768883	0.788313793	0.025165796	0.03870165
K02396	0.000300362	0.000546956	1.82099076	0.025246643	0.038796
K05772	4.00E-05	0.000114537	2.863485415	0.025392839	0.038978784
K01905	4.01E-05	0.000205242	5.116523501	0.025412155	0.038978784
K10112	0.000270544	0.000275289	1.017535558	0.025424352	0.038978784

K06200	0.000297075	0.000920025	3.09694471	0.025746139	0.039441737
K02871	0.001139942	0.001384883	1.214871492	0.025820756	0.039451252
K09922	4.19E-05	0.000188365	4.492608656	0.025985479	0.039451252
K06994	9.80E-06	0	0	0.02601448	0.039451252
K05846	7.85E-06	0	0	0.02601448	0.039451252
K01654	1.17E-05	0	0	0.02601448	0.039451252
K09780	1.43E-05	0	0	0.02601448	0.039451252
K07067	9.61E-06	0	0	0.02601448	0.039451252
K00244	5.53E-07	0	0	0.02601448	0.039451252
K07451	3.32E-05	0	0	0.02601448	0.039451252
K07223	1.20E-05	0	0	0.02601448	0.039451252
K21885	1.41E-05	0	0	0.02601448	0.039451252
K01919	1.60E-05	0	0	0.02601448	0.039451252
K09819	1.10E-05	0	0	0.02601448	0.039451252
K03602	8.38E-05	0.000178107	2.124982468	0.026029896	0.039451252
K00656	0.000196111	0.000222184	1.132948895	0.026114815	0.039549834
K02416	8.43E-05	0.000192247	2.280306024	0.026221295	0.039680896
K03588	0.000119287	0.000223026	1.869654985	0.02656091	0.040164293
K02663	2.27E-05	5.45E-05	2.402332359	0.026721403	0.040214315
K00262	0.002688381	0.00206501	0.768123907	0.026832603	0.040214315
K09181	0	7.40E-06	inf	0.026856696	0.040214315
K20107	7.61E-06	0	0	0.026856696	0.040214315
K20108	7.61E-06	0	0	0.026856696	0.040214315
K03319	0	4.73E-05	inf	0.026856696	0.040214315
K03187	0	4.56E-06	inf	0.026856696	0.040214315
K19294	2.75E-05	0	0	0.026856696	0.040214315

K18471	0	2.95E-05	inf	0.026856696	0.040214315
K02777	7.61E-06	0	0	0.026856696	0.040214315
K15773	0	3.97E-05	inf	0.026856696	0.040214315
K17195	0	3.15E-05	inf	0.026856696	0.040214315
K13570	0	0.00010034	inf	0.026856696	0.040214315
K02810	0.000175727	0.000542286	3.085954426	0.02728476	0.040793893
K02809	0.000175727	0.000542286	3.085954426	0.02728476	0.040793893
K09770	0.000166535	0.000304439	1.828075705	0.027419677	0.040938655
K09766	0.000143677	0.000270478	1.882545026	0.027422727	0.040938655
K02034	0.000963265	0.002413606	2.505651142	0.027665318	0.041269852
K03282	0.000590783	0.000422433	0.715039031	0.02793382	0.041639178
K07107	0.000403625	0.000590722	1.463544153	0.028070287	0.04181128
K19221	0.000434035	0.000256772	0.591592258	0.028101715	0.041826786
K02073	0.00014588	0.000378381	2.593780873	0.028157462	0.041878438
K01273	0.000168261	0.000342382	2.034819936	0.028791103	0.042787555
K02124	0.000291282	0.000239029	0.820608179	0.02881172	0.042787555
K00077	0.000169561	0.00032852	1.93747241	0.028885444	0.042865052
K00567	6.89E-05	0.000216967	3.146772643	0.029548035	0.04381564
K06140	3.85E-05	0.000116072	3.012873303	0.029693147	0.04397211
K00600	0.002210534	0.001870374	0.846118695	0.029697746	0.04397211
K15342	4.07E-05	0.000227061	5.574354168	0.029723251	0.043977151
K06382	0.000943649	0.00054387	0.576348166	0.029768124	0.044010823
K09155	1.82E-05	0.000173357	9.549715894	0.030414416	0.044932954
K02687	0.00052839	0.000690317	1.306454403	0.031256399	0.046142606
K03734	0.000372976	0.000558638	1.497785626	0.031290922	0.046159328
K03701	0.001267877	0.000756883	0.596968721	0.031497331	0.046429399

K10118	0.000159639	0.000293149	1.836331807	0.031604106	0.046539763
K02988	0.002516959	0.002005936	0.796968352	0.031618974	0.046539763
K04769	0.000274575	0.000399775	1.45597849	0.03181233	0.046789753
K02049	6.13E-05	0.000192446	3.137952129	0.031862834	0.046829423
K18981	2.13E-05	6.80E-05	3.186445692	0.031984157	0.046973043
K13954	1.51E-05	6.38E-06	0.421542736	0.032193505	0.047245631
K00881	5.56E-05	5.59E-05	1.006635781	0.032238962	0.047277476
K03790	0.000813262	0.000455418	0.559989081	0.032280226	0.047303129
K05825	5.37E-05	0.000232142	4.320041567	0.032304681	0.047304133
K09913	4.94E-05	9.49E-05	1.919920615	0.032392371	0.04739766
K06872	0.000199219	0.000512296	2.571524068	0.032504585	0.04752691
K15633	0.002307091	0.001830627	0.793478492	0.032819149	0.04795162
K07053	2.12E-05	6.97E-05	3.285835224	0.033138434	0.0483826
K03087	2.22E-05	6.64E-05	2.983611359	0.033333543	0.048613518
K00784	0.000620894	0.000871792	1.404090513	0.033345453	0.048613518
K00330	0.00132459	0.000959086	0.724062631	0.033451555	0.048732499
K06902	0.000170127	0.000317409	1.865714447	0.03382207	0.049236224
K06346	0.000183882	0.000447899	2.435796803	0.033961521	0.04940309
K00243	0.000301027	0.000413207	1.372655414	0.034005193	0.049430486
K21471	0.000669547	0.00093973	1.40353216	0.034060402	0.049474598
K07003	0.000473107	0.000293727	0.620845951	0.034101473	0.049498126

Table S7 The differential KO-enriched metabolism pathways between SCAD patients and HCs.

ID	Description	GeneRatio	BgRatio	pvalue	p.adjust	qvalue
map01230	Biosynthesis of amino acids	80/552	237/5774	5.14E-26	5.86E-24	3.79E-24
map01240	Biosynthesis of cofactors	101/552	375/5774	5.32E-24	3.03E-22	1.96E-22
map00620	Pyruvate metabolism	34/552	109/5774	1.68E-10	6.37E-09	4.12E-09
map00010	Glycolysis / Gluconeogenesis	30/552	105/5774	2.04E-08	5.81E-07	3.76E-07
map00270	Cysteine and methionine metabolism	31/552	117/5774	8.16E-08	1.86E-06	1.20E-06
map00020	Citrate cycle (TCA cycle)	20/552	61/5774	4.25E-07	8.08E-06	5.22E-06
map00640	Propanoate metabolism	28/552	110/5774	8.56E-07	1.39E-05	9.01E-06
map01200	Carbon metabolism	61/552	356/5774	2.82E-06	4.02E-05	2.60E-05
map00720	Carbon fixation pathways in prokaryotes	26/552	106/5774	4.47E-06	5.23E-05	3.38E-05
map00450	Selenocompound metabolism	13/552	33/5774	4.59E-06	5.23E-05	3.38E-05
map00230	Purine metabolism	41/552	210/5774	5.38E-06	5.57E-05	3.60E-05
map00400	Phenylalanine, tyrosine and tryptophan biosynthesis	19/552	71/5774	2.35E-05	0.000222845	0.000144037
map00650	Butanoate metabolism	22/552	99/5774	0.000121096	0.001061916	0.000686372
map00260	Glycine, serine and threonine metabolism	22/552	100/5774	0.000141666	0.001153565	0.00074561
map00860	Porphyrin and chlorophyll metabolism	26/552	129/5774	0.000168733	0.001282368	0.000828862
map00910	Nitrogen metabolism	16/552	65/5774	0.000297133	0.001825163	0.001179699
map00500	Starch and sucrose metabolism	22/552	105/5774	0.000298151	0.001825163	0.001179699
map00220	Arginine biosynthesis	15/552	59/5774	0.000312856	0.001825163	0.001179699
map00300	Lysine biosynthesis	13/552	47/5774	0.000320204	0.001825163	0.001179699
map00340	Histidine metabolism	13/552	47/5774	0.000320204	0.001825163	0.001179699
map00240	Pyrimidine metabolism	21/552	100/5774	0.000393908	0.002138357	0.001382133
map00670	One carbon pool by folate	11/552	37/5774	0.000466904	0.002419414	0.001563795
map00540	Lipopolysaccharide biosynthesis	15/552	62/5774	0.000556893	0.002749545	0.001777176

map00790	Folate biosynthesis	18/552	82/5774	0.000578852	0.002749545	0.001777176
map00051	Fructose and mannose metabolism	22/552	111/5774	0.000671343	0.003028348	0.001957381
map00190	Oxidative phosphorylation	36/552	218/5774	0.000690676	0.003028348	0.001957381
map00250	Alanine, aspartate and glutamate metabolism	16/552	70/5774	0.000723873	0.003056352	0.001975481
map00750	Vitamin B6 metabolism	8/552	24/5774	0.001221053	0.004971428	0.003213296
map00290	Valine, leucine and isoleucine biosynthesis	7/552	19/5774	0.001271321	0.004997605	0.003230216
map01210	2-Oxocarboxylic acid metabolism	17/552	82/5774	0.001602338	0.006088884	0.003935567
map00521	Streptomycin biosynthesis	7/552	20/5774	0.001796886	0.006607903	0.004271036
map00770	Pantothenate and CoA biosynthesis	11/552	44/5774	0.002258232	0.008044952	0.005199877
map00520	Amino sugar and nucleotide sugar metabolism	25/552	147/5774	0.002942239	0.010164098	0.006569593
map00780	Biotin metabolism	7/552	23/5774	0.004410238	0.014787268	0.009557791
map00710	Carbon fixation in photosynthetic organisms	9/552	36/5774	0.005575057	0.017654346	0.011410935
map00730	Thiamine metabolism	9/552	36/5774	0.005575057	0.017654346	0.011410935
map00061	Fatty acid biosynthesis	9/552	39/5774	0.009695076	0.029871315	0.019307406

Table S8 The metabolites differentially abundant between SCAD patients and HCs.

Metabolites	Normal_mean	SCAD_mean	FC	p_value	FDR	Class	VIP
5_Dodecenoic acid	0.659600115	0.369674017	0.560451718	5.65E-06	0.001090222	Fatty acids	0.467364363
Docosahexaenoic acid DHA	0.745504562	0.391518046	0.525171898	5.27E-05	0.004160551	Fatty acids	0.324986424
Eicosapentaenoic acid EPA	0.866790053	0.376627906	0.4345088	0.000100469	0.004160551	Fatty acids	0.627204717
Heptadecanoic acid	0.525546899	0.337239639	0.641692758	0.000107786	0.004160551	Fatty acids	0.486887458
Docosapentaenoic acid DPA	0.670884232	0.364910178	0.543924213	0.00016339	0.005255727	Fatty acids	1.924938041
Glutarylcarnitine	0.398162971	0.534410726	1.342190927	0.000262132	0.007227357	Carnitines	1.395088845
Myristelaidic acid	0.573546485	0.347365788	0.605645395	0.000364379	0.008790643	Fatty acids	0.495478543
8,11,14_Eicosatrienoic acid	0.506334236	0.339022227	0.669562125	0.000471869	0.010118964	Fatty acids	0.523886287
Methionine sulfoxide	0.658158058	0.440471408	0.669248674	0.000608379	0.011741708	Amino acids	1.404272807
Docosapentaenoic acid 22n_6	0.515983055	0.333464555	0.64627036	0.000733979	0.012332201	Fatty acids	0.451655933
Acetylglycine	0.549980653	0.459774485	0.835983017	0.000780935	0.012332201	Amino acids	1.07525645
Dimethylglycine	0.365464856	0.514452106	1.407665053	0.000830666	0.012332201	Amino acids	0.640973058
Palmitoleic acid	0.594919917	0.348366331	0.585568445	0.001196179	0.016490185	Fatty acids	0.750536186
L_Alpha_aminobutyric acid	0.614508997	0.496638474	0.808187473	0.001608797	0.018264581	Amino acids	0.515554917
Glycodeoxycholic acid	0.285311538	0.780406869	2.735279743	0.001608797	0.018264581	Bile acids	1.299119543
Malonic acid	0.443335176	0.849864463	1.916979543	0.001608797	0.018264581	Organic acids	0.971504348
3_Phenylbutyric acid	0.408790472	0.745521582	1.823725436	0.001915583	0.018485377	Phenylpropanoic acids	0.760957198
Myristoylcarnitine	0.365085444	0.440744276	1.20723596	0.001915583	0.018485377	Carnitines	1.335094774
L_threonine	0.59272025	0.432739382	0.73009043	0.001915583	0.018485377	Amino acids	1.80627911
Acetic acid	0.707181109	0.523088644	0.739681303	0.002851193	0.026203822	Fatty acids	1.549680108
10Z_Heptadecenoic acid	0.535287435	0.333431435	0.622901667	0.004187689	0.035140177	Fatty acids	0.578426494
L_Pipecolic acid	0.578981776	0.475415876	0.821124076	0.004187689	0.035140177	Amino acids	0.928988081

L_Homocitrulline	0.426885515	0.584079565	1.368234678	0.005185475	0.041699858	Amino acids	1.237785572
Alpha_Linolenic acid	0.739710231	0.414136532	0.559863194	0.00546637	0.042200379	Fatty acids	0.534525288
Linoleic acid	0.521982124	0.398415283	0.763273807	0.006069726	0.042343399	Fatty acids	0.150044098
Lauroylcarnitine	0.289379521	0.424300476	1.466242236	0.006069726	0.042343399	Carnitines	1.129659555
Decanoic acid	0.516589304	0.381905068	0.739281796	0.006393354	0.042343399	Fatty acids	0.707018829
4_Hydroxybenzoic acid	0.580287288	0.39255068	0.676476443	0.006393354	0.042343399	Benzenoids	0.62490102
L_Cystine	0.579867688	0.444338599	0.766275839	0.006732427	0.042343399	Amino acids	1.046312572
Myristoleic acid	0.559759434	0.343897559	0.614366705	0.007087577	0.042343399	Fatty acids	1.322788515
D_Maltose and Alpha_Lactose	0.509312461	0.911961746	1.790574185	0.007459459	0.042343399	Carbohydrates	1.595506177
Oleic acid	0.501394454	0.362346126	0.722676774	0.007459459	0.042343399	Fatty acids	0.54910489
D_2_Hydroxyglutaric acid	0.430362833	0.884570996	2.055407503	0.009128108	0.048936799	Organic acids	0.710716792
3_Hydroxyisovaleric acid	0.587274525	0.479646658	0.816733295	0.009128108	0.048936799	Fatty acids	1.094992155
